# Author Correction: High-resolution Nanopore methylome-maps reveal random hyper-methylation at CpG-poor regions as driver of chemoresistance in leukemias

**DOI:** 10.1038/s42003-023-04842-x

**Published:** 2023-04-25

**Authors:** Alberto Magi, Gianluca Mattei, Alessandra Mingrino, Chiara Caprioli, Chiara Ronchini, Gianmaria Frigè, Roberto Semeraro, Davide Bolognini, Alessandro Rambaldi, Anna Candoni, Emanuela Colombo, Luca Mazzarella, Pier Giuseppe Pelicci

**Affiliations:** 1 Department of Information Engineering, University of Florence, Florence, Italy; 2 Institute for Biomedical Technologies, National Research Council, Segrate, Milano, Italy; 3 Department of Experimental and Clinical Medicine, University of Florence, Florence, Italy; 4 Department of Experimental Oncology, IEO European Institute of Oncology IRCCS, Milano, Italy; 5 Department of Oncology and Hemato-Oncology, University of Milan, Milan, Italy; 6 Azienda Socio-Sanitaria Territoriale Papa Giovanni XXIII, Bergamo, Italy; 7 Clinica Ematologica, Azienda Sanitaria Universitaria Integrata di Udine, Udine, Italy

**Keywords:** Cancer genomics, Epigenomics, Software

Correction to: *Communications Biology* 10.1038/s42003-023-04756-8, published online 08 April 2023.

In this Article the author name Gianmaria Frigè was incorrectly written as GianMaria Frigè and this author was incorrectly affiliated with the Department of Oncology and Hemato-Oncology, University of Milan, Milan, Italy. The original Article has been corrected.

