# Supplementary material for: High-resolution Nanopore methylome-maps reveal random hyper-methylation at CpG-poor regions as driver of chemoresistance in leukemias
Source: Commun Biol. 2023 Apr 8;6:382. doi: 10.1038/s42003-023-04756-8 (PMC10082806; doi:10.1038/s42003-023-04756-8)
Supplement: Supplementary file 2 — Supplemental Material [file 42003_2023_4756_MOESM2_ESM.pdf]

Supplemental Material to: "High-resolution Nanopore methylome-maps reveal random hyper-methylation at CpG-poor regions as driver of chemoresistance in leukemias."

Alberto Magi[1,2,+,*], Gianluca Mattei[1,+], Alessandra Mingrino[3], Chiara Caprioli[4,5], Chiara Ronchini[4], GianMaria Frigè[4,5], Roberto Semeraro[3], Davide Bolognini[3], Alessandro Rambaldi[5,6], Anna Candoni[7], Emanuela Colombo[4], Luca Mazzarella[4], Pier Giuseppe Pelicci[4,5].

[1]Department of Information Engineering, University of Florence, Florence, Italy, albertomagi@gmail.com. [2]Institute for Biomedical Technologies, National Research Council, Segrate, Milano, Italy. [3]Department of Experimental and Clinical Medicine, University of Florence, Florence, Italy. [4]Department of Experimental Oncology, IEO European Institute of Oncology IRCCS, Milano, Italy. [5]Department of Oncology and Hemato-Oncology, University of Milan, Milan, Italy. [6]Azienda Socio-Sanitaria Territoriale Papa Giovanni XXIII, Bergamo, Italy. [7]Clinica Ematologica, Azienda Sanitaria Universitaria Integrata di Udine, Udine, Italy. [*] **Correspondence**: Alberto Magi, Department of Information Engineering, University of Florence, 50100, Florence, Italy (albertomagi@gmail.com), Pier Giuseppe Pelicci, Department of Experimental Oncology, IEO European Institute of Oncology IRCCS, Milano, Italy (piergiuseppe.pelicci@ieo.it) . [+] These authors contributed equally to this work.

Nanopore library preparation and sequencing

DNA was qualitatively and quantitatively analyzed using Nanodrop and Qubit respectively. The sequencing was performed on the GridION 5X platform (Oxford Nanopore Technologies, Oxford, UK). Library preparation was performed according to the Oxford Nanopore Technologies (ONT) manufacturer's protocol for genomic DNA, the Ligation Sequencing Kit 1D SQK-LSK109. For each library 1 ug of DNA as starting material was used. The input DNA for all nanopore libraries was unshared. Initially DNA was mixed with 3,5 ul of NEBNext FFPE DNA Repair Buffer and 2 ul of NEBNext FFPE DNA Repair Mix (M6630, NEB, Ipswich, Massachusetts) in order to repair possible nicks, and then end-prepped adding 3,5 ul of Ultra II End-prep reaction buffer and 3 ul of Ultra II End-prep enzyme mix (E7546, NEB). The reaction was incubated at 20°C for 5 minutes and 65°C for 5 minutes, followed by a purification step with 60 ul of 1X AMPure XP beads, by a washing and an elution. The end-repaired DNA was ligated with 5 ul Adapter Mix (AMX, ONT) using 10 ul NEBNext Quick T4 DNA Ligase (E6056, NEB) for 10 min at room temperature. The adapted-ligated DNA was cleaned up by adding 40 ul of 1X AMPure XP beads and incubated at room temperature for 5 min. The beads were pelleted on a magnetic rack and pellet was washed twice by resuspending in 250 ul Long Fragment Buffer (LFB, ONT) in order to enrich for long molecules. Then, the reaction was eluted in 15 ul of Eluition Buffer (EB, ONT) at room temperature for 10 minutes. The prepared library was quantified by Qubit and then, prior to loading onto the flow cell, was mixed with 37,5 ul of Sequencing Buffer (SB, ONT) and 25,5 ul of Loading Beads (LB, ONT). According to the ONT's protocol before loading, each R9.4.1 SpotON flow cell (FLO-MIN106D) was primed mixing the Flush Tether (FLT, ONT) directly to the tube of Flush Buffer (FB, ONT) and loading this mix via the priming port. Libraries were sequenced on a GridION 5x device and, in order to maximize the total throughput, sequencing was carried out for 72 h.

Shifting Level Models with truncated gaussian

Classical SLM are a class of models (Magi *et al.*, 2010) in which sequential observations $x = (x_1, \ldots, x_i, \ldots, x_N)$ are considered to be realizations of the sum of two independent stochastic processes

$$x_i = m_i + \epsilon_i,$$

$$m_i = (1 - z_{i-1}) \cdot m_{i-1} + z_{i-1} \cdot (\mu + \delta_i)$$

where m_i is the unobserved mean level and ϵ_i is normally distributed white noise. The process m_i changes its value independently of m_{i-1} and is controlled by the process z_i: when $z_{i-1} = 0$, m_i is the same as m_{i-1} and when $z_{i-1} = 1$, m_i is incremented by the normal random variable δ_i ($\delta_i \sim N(0, \sigma_\mu^2)$). $z_1, z_2, ...$ are independent and identically distributed random variables taking the values 0,1 with probabilities $\eta = Pr(z_i = 1)$, $1 - \eta = Pr(z_i = 0)$.

To take into account the sparse distribution of CpGs along the genome, as in (Magi *et al.*, 2013) we extended the classical SLM to an heterogeneous form, where the probability $Pr(z_i = 1)$ depends on this distance between consecutive CpGs (d_i) with the following formula:

$\Pr(\mathrm{z}_i = 1) = \eta(d_i) = \frac{1}{2} \cdot \theta + ((\frac{1}{2} - \theta) \cdot exp\left[\frac{log(\theta)}{\frac{d_i}{d_{Norm}}}\right]) \ (1)$

where $\eta(d_i)$ is the probability of random variables z_i to be equal to 1, θ is a constant parameter , d_i is the distance between the $i - th$ and $i_1 - th$ CpG and d_{Norm} is the distance normalization parameter. Equation 1 defines the dependence between the probability $Pr(z_i = 1)$ and the genomic distance between adjacent CpGs d_i: the larger is d_i and the larger is $Pr(z_i = 1)$ and consequently the larger is the probability to jump between two mean levels m_i. The constant parameter θ can be seen as the baseline probability of random variables z_i to take value 1 while the d_{Norm} parameter modulates the genomic distance at which the probability $Pr(z_i = 1)$ begins to grow: for distances smaller than d_{Norm} the probability $Pr(z_i = 1) = (\theta - \theta^2) \sim \theta$, while when d_i is larger than d_{Norm} the probability $Pr(z_i = 1)$ grows until reaching the value 1.

The expected value of x_i is equal to μ and, since the two stochastic processes are independent, the variance of x_i is the sum of the variances of the two processes:

$$E[x_i] = \mu, \tag{2}$$

$$var[x_i] = \sigma_\mu^2 + \sigma_\epsilon^2. \tag{3}$$

In this way, SLM allows one to break up the total variance of the genomic profile in two parts: the biological variance (σ_μ^2) and the experimental variance (σ_ϵ^2). Using (3) we can introduce a different parametrization of the SLM by defining the parameter $\omega = \sigma_\mu^2/\sigma^2$ (with $\sigma^2 = var[x_i]$) such that σ_μ^2 by $\omega \cdot \sigma^2$ and σ_ϵ^2 by $(1 - \omega)\sigma^2$.

With this new parametrization the joint probability distribution of the observations and latent variables $p(x, m, z|\Theta)$, given the parameters, can be explicated in the following:

$$\begin{aligned} p(x, m, z|\theta) &= p(x|m, \sigma^2, \omega) \cdot p(m|z, \mu, \sigma^2, \omega) \cdot p(z|\eta(d_i)) = \\ &= \prod_{i=1}^{N} p(x_i|m_i, \sigma^2, \omega) \cdot p(m_0) \times \prod_{i=0}^{N} p(m_{i+1}|m_i, z_i, \mu, \sigma^2, \omega) \cdot p(z_i|\eta(d_i)), \end{aligned} \tag{4}$$

Equation 4 defines an Heterogeneous Hidden Markov Model (HHMM) of order one, in which a single state variable, $q_i = (m_i, z_i)$, summarizes all the relevant past information of the underlying process. In the model defined by (4) the elements of the HHMM are the following:

- the state transition probability distribution is:
  $p(q_{i+1}|q_i, \theta) = p(m_{i+1}|m_i, z_i, \mu, \sigma^2, \omega) \cdot p(z_i|\eta(d_i))$
- the emission probability distribution is:
  $p(x_i|q_i, \theta) = p(x_i|m_i, \sigma^2, \omega)$
- the initial state probability distribution is:
  $p(q_0|\theta) = p(m_0|\mu, \sigma^2, \omega)$

Since $\Delta\beta$ takes values in the range [-1,1], we modeled ϵ_i with a truncated gaussian distributions with upper and lower bound 1 and -1 respectively.

The fact that SLM is an HMM allows us to make use of the several algorithms developed for these kinds of models. To handle the infinite dimensionality of SLM, we use the same approach we previously developed in (Magi *et al.*, 2010).
We introduce a Markovian stochastic process $s_1, s_2, ..., s_k$ taking values in $S = \{1, 2, ..., K\}$. From equation 4, we know that the probability distribution of x_i, given the parameters, has the following form:

$$p(x_i|m_i, \sigma^2, \omega) = N(x_i|m_i, (1-\omega) \cdot \sigma^2), \tag{5}$$

Since $\Delta\beta$ takes values in the range [-1,1], we assume that the conditional probability of x_i, given $s_i = k$ is a truncated gaussian distributions with upper and lower bound 1 and -1 respectively, $N_{-1}^{1}(\mu_k, \sigma_\epsilon^2)$.

The parameter $\mu_k (k = 1, 2, ..., K)$ is associated to each state of the Markovian stochastic process and represents an approximation of the m_i latent variables of the SLM.
The emission function of the HMM is defined as follows:

$$f_k(x) = \frac{1}{\sqrt{2\pi}\sigma_\epsilon} \exp\left[-\frac{1}{2}\left(\frac{x-\mu_k}{\sigma_\epsilon}\right)^2\right] \cdot \frac{1}{\Phi(\frac{1-\mu_k}{\sigma_\epsilon}) - \Phi(\frac{-1-\mu_k}{\sigma_\epsilon})}. \tag{6}$$

where Φ represent the cumulative distribution functions of a non-truncated Gaussian of parameter μ_k and σ_ϵ.

To complete the description of the model, it remains to specify the state transition matrix P. From (4) the state transition probability has the following form:

$$\begin{aligned} p(m_{i+1}|m_i, z_i, \mu, \sigma^2, \omega) \cdot p(z_i|\eta(d_i)) &= [(1-z_i) \cdot \delta(m_{i+1} - m_i) + z_i \cdot N(m_{i+1}|\mu, \omega \cdot \sigma^2)] \cdot \\ &\quad \cdot [\eta(d_i) \cdot \delta(z_i - 1) + (1 - \eta(d_i)) \cdot \delta(z_i)], \end{aligned} \tag{7}$$

hence the state transition matrix is:

$$P_{jk} = \begin{cases} (1 - \eta(d_i)) + \eta(d_i) \cdot g_{jk} & j = k \\ \eta(d_i) \cdot g_{jk} & j \neq k \end{cases} \tag{8}$$

where

$$\begin{aligned} g_{jk} &= c_j \cdot e^{-\frac{(\mu_k - \mu)^2}{2\sigma_\mu^2}}, \\ c_j &= \left(\sum_{k=1}^{K} e^{-\frac{(\mu_k - \mu)^2}{2\sigma_\mu^2}} \right)^{-1}. \end{aligned} \tag{9}$$

To estimate the parameters of the Heterogeneous truncated-gaussian Shifting Level Model (HT-GSLM), we develop a two-step algorithm that follows our previous idea in Magi *et al.* (2013). Since the$\Delta\beta$ can take values in a well-defined range, we used a large number of states K0 and we choose μ_k in order to densely and homogeneously cover the range [-1,1] instead of estimating the μ_k parameters by using the Baum and Welch algorithm (Magi *et al.*, 2010). Moreover, since we expect that the great majority of CpG have no differential methylation between samples ($\Delta\beta \sim 0$), we initialize $\mu = 0$. This simple solution drastically improves the computational performance of our algorithm without affecting its accuracy in the detection of signal shifts.

In the first step of the algorithm we initialize the mean μ and the variances σ^2, σ_μ^2 and σ_ϵ^2 with the following formulas:

$$\begin{aligned} &\mu = 0, \\ &\sigma = \sqrt{\frac{\sum_{i=1}^{N} (x_i - \mu)^2}{(N-1)}}, \\ &\sigma_\mu^2 = \omega \cdot \sigma^2, \\ &\sigma_\epsilon^2 = (1 - \omega) \cdot \sigma^2. \end{aligned} \tag{10}$$

In the second step we apply the Viterbi algorithm to find the best state sequence $s^{(j)}$ and estimate the points of mean shift z_i. After Viterbi algorithm we calculate the median of the $\Delta\beta$ values that belong to each segment.

The inputs to the algorithm are the $\Delta\beta$ values $\Delta\beta = (\Delta\beta_1, .., \Delta\beta_i, .., \Delta\beta_N)$ to be segmented, the distance between adjacent CpGs $d = (d_1, ..., d_i, ..., d_N)$, the number of states $K^{(0)}$, the parameter ω, the parameter θ, and the distance normalization parameter d_{Norm}.

The meaning of the parameters θ and ω is the same we previously stated in Magi *et al.* (2011). The

parameter ω modulates the proportionality between the white noise stochastic process (σ_ϵ^2) and the means jump stochastic process (σ_μ^2). For large values of ω (small σ_ϵ and large σ_μ) the algorithm 'sees' the signal to have small white noise and takes as level shift also slight variations of the genomic profile. On the contrary when ω is small a large fraction of the total variance σ^2 is assigned to the σ_ϵ (σ_ϵ is large and σ_μ is small) and only sizeable variations of the signal are taken as level shift. The parameter θ corresponds to the baseline probability that a transition to a new mean level occurs at any position i of the sequential process and is able to control only specificity and has weak effect on sensitivity. The parameter $K^{(0)}$ regulates the density of the μ_k in the range [-1,1]: the larger is $K^{(0)}$ and the larger is the μ_k density in the interval [-h,h]. The use of predefined μ_k values in the range [-1,1] does not affect the results of the segmentation procedure. This is due to the fact that the Viterbi algorithm is able to correctly identify all the mean shifts (z_i) also when the μ_k value does not perfectly match with the real value of the segments. In fact, when a state m_i does not have its associated μ_k, the Viterbi algorithm associates m_i to the most lilkely μ_k, allowing for the identification of all the mean shifts z_i. Finally, the distance normalization parameter d_{Norm} modulates the ability of the segmentation algorithm to detect both small and highly isolated CpG of the genome and large and highly CpG-covered epi-genomic alterations. After segmentation, each segment is tested for differential methylation by using the Wilcoxon rank-sum test.

Algorithm comparison

To test the ability of our segmentation algorithm to detect DMRs with different noise and degrees of methylation difference, we used the synthetic dataset generated in (Juhling *et al.*, 2016). In (Juhling *et al.*, 2016) the authors simulated DMRs on the human chromosome 10 (hg19) by exploiting the ENCODE chromatin state segmentation of the GM12878 cell line and using a Beta-Binomial approach to simulate both biological methylation and the sequencing step. By using a very complex recipe they simulated two different noise levels (low noise, background 1 and high noise, background 2) and four different degrees of methylation (with decreasing difference from class 1 to class 4) resulting in eight data sets with different levels of complexity, from easily (class 1 DMRs – background 1) to difficultly (class 4 DMR on background 2) distinguishable. For each of the height different level of complexity they simulated 20 samples, split in into two groups of 10 samples, and they introduced an equal number of hypermethylated DMRs (500) and hypomethylated DMRs (500).

To find the best parameter setting of our algorithm we applied it on the synthetic dataset of (Juhling *et al.*, 2016) by using $\omega = [0.1, 0.2, 0.3, 0.4, 0.5, 0.6, 0.7]$, $\theta = [10^-3, 10^-4, 10^-5, 10^-6, 10^-7]$ and $d_{Norm} = [10^4, 10^5, 10^6, 10^7]$. To use it on the analysis of multiple samples, for each CpG, we calculated the mean difference signal (MDS) with the following formula:

$$MDS_{CpG} = \frac{\sum_i \beta^i_{CpG}}{N} - \frac{\sum_j \beta^j_{CpG}}{M}. \tag{11}$$

where, for each CpG, $beta^i_{CpG}$ are the β values of the N test samples and $beta^j_{CpG}$ are the β values of the M test samples.

To evaluate the performance of our method we calculated precision as the ratio between the number of true positive (TP) DMRs and the total number of called DMRs and recall as the ratio between the number of TP and the total number of simulated DMRs: a detected segment is considered a TP if there is at least a 50% overlap with the simulated DMRs. The results reported in Supplemental Figure 1-6 show that for low complexity datasets (low noise and high methylation differences) our method obtains the best results with $\omega = [0.2, 0.3]$ and $\eta = [10^-4, 10^-3]$, while for high complexity dataset (high noise and low methylation differences) the best F-score is obtained with $\omega = [0.5, 0.6]$ and $\eta = [10^-7, 10^-6]$. Concerning the d_{Norm} parameter, it has no effect on the global performance

As a further step we compared the performance of our method with those of two previously published algorithms: Metilene (Juhling *et al.*, 2016) and BSmooth (Hansen *et al.*, 2012). We downloaded Metilene version 0.2-8 from `https://www.bioinf.uni-leipzig.de/Software/metilene/Downloads/` and the bsseq R package (that contains the BSmooth algorithm) from Bioconductor and we ran them with default parameters settings. The results of Supplemental Figure 8 demonstrate that our segmentation method outperforms the other two state-of-the-art methods for all the class of data complexity (high and low noise) especially in the analysis of a small number of samples (Supplemental Figure 8.e-f).

Visual inspection

Visual inspection of aligned reads is a fundamental step in the identification of genomic variants since the advent of SGS technologies. The discovery of putative variants is performed by automated computational pipelines, but visual review remains critical to the verification and interpretation of these variants (Robinson *et al.*, 2017), especially for SVs with long read data that are inferred by using a very complex combination of alignment signatures (gapped alignment and split read alignment or read-depth).

Although NanomonSV combines gapped alignment and split read alignment signatures, small deletions and insertions ($<$ 5 kb) are generally inferred by using gapped alignment signatures, while inversions, duplication, translocations and large insertions and deletions (larger than 5 kb) are detected by exploiting split read signature.

For these reasons, small deletions and insertions were visually validated by using the Integrative Genomics Viewer (IGV version 2.9.4) (Robinson *et al.*, 2011) while duplications, inversions and large insertions and deletions by using Samplot version 1.3.0 (Belyeu *et al.*, 2021).

IGV was one of the first tools to provide SGS data visualization and recently has been updated with a suite of specialized features to support third-generation long-read sequencing technologies. Samplot is a command line tool that allows to produce high-quality images that highlight any split-alignment and depth signals that support SV.

The results of visual inspection of the SVs identified by NanomonSV in the three AML pairs are reported in Figure 43-55 .

References

Juhling F, Kretzmer H, Bernhart SH, Otto C, Stadler PF, Hoffmann S. metilene: fast and sensitive calling of differentially methylated regions from bisulfite sequencing data. Genome Res. 2016 Feb;26(2):256-62.

Magi A, Benelli M, Marseglia G, Nannetti G, Scordo MR, Torricelli F. A shifting level model algorithm that identifies aberrations in array-CGH data. Biostatistics. 2010 Apr;11(2):265-80.

Magi A, Benelli M, Yoon S, Roviello F, Torricelli F. Detecting common copy number variants in high-throughput sequencing data by using JointSLM algorithm. Nucleic Acids Res. 2011 May;39(10):e65.

Magi A, Tattini L, Cifola I, D'Aurizio R, Benelli M, Mangano E, Battaglia C, Bonora E, Kurg A, Seri M, Magini P, Giusti B, Romeo G, Pippucci T, De Bellis G, Abbate R, Gensini GF. EXCAVATOR: detecting copy number variants from whole-exome sequencing data. Genome Biol. 2013;14(10):R120.

Robinson JT, Thorvaldsdóttir H, Wenger AM, Zehir A, Mesirov JP. Variant Review with the Integrative Genomics Viewer. Cancer Res. 2017 Nov 1;77(21):e31-e34.

Juhling F, Kretzmer H, Bernhart SH, Otto C, Stadler PF, Hoffmann S. metilene: fast and sensitive calling of differentially methylated regions from bisulfite sequencing data. Genome Res. 2016 Feb;26(2):256-62.

Hansen KD, Langmead B, Irizarry RA. BSmooth: from whole genome bisulfite sequencing reads to differentially methylated regions. Genome Biol. 2012 Oct 3;13(10):R83.

Robinson JT, Thorvaldsdóttir H, Winckler W, Guttman M, Lander ES, Getz G, Mesirov JP. Integrative genomics viewer. Nat Biotechnol. 2011 Jan;29(1):24-6.

Belyeu JR, Chowdhury M, Brown J, Pedersen BS, Cormier MJ, Quinlan AR, Layer RM. Samplot: a platform for structural variant visual validation and automated filtering. Genome Biol. 2021 May 25;22(1):161.

Table 1: AML samples characteristics.

Sample	Sex	Age	FAB	Karyotype	Molecular profile	1st line treatment (induction, maintenance)
AML2	F	20	M2	XX,46	STAG2	Idarubicin + HiDAC, AraC
UD5	M	67	myelodysplastic changes	XY,46	NPM1	FLAI, Idarubicin + AraC, HiDAC + autoHSCT
UD10	F	22	M4	inv(16)	NA	FLAI, Idarubicin + AraC, HiDAC

The table reports the main features at diagnosis of the three AML patients: Sex, Age, French-American-British (FAB) classification, the Karyotype, the Molecular profile and the 1st line treatment. Molecular profile was obtained through Illumina targeted sequencing of bone marrow aspirate performed as per clinical routine. NA, not available; HiDAC, high-dose cytarabine; AraC, standard-dose cytarabine; FLAI, fludarabine, cytarabine, idarubicin; autoSCT, autologous hematopoietic stem cell transplantation.

Table 2: CpG groups inferred by Nanopolish.

Sample	CpG Groups	1 CpG site	2 CpG sites	3 CpG sites	4 CpG sites	≥5 CpG sites	Total CpG sites
UD5T	22687510	19191168 (84.5%)	2461372 (10.9%)	635351 (2.8%)	217004 (1%)	182615 (0.8%)	28181699
UD5R	22689245	19192734 (84.5%)	2461413 (10.9%)	635404 (2.8%)	217043 (1%)	182651 (0.8%)	28183843
UD10T	22578261	19093348 (84.5%)	2452640 (10.9%)	633692 (2.8%)	216224 (1%)	182357 (0.8%)	28056760
UD10R	22574289	19089697 (84.5%)	2452425 (10.9%)	633637 (2.8%)	216184 (1%)	182346 (0.8%)	28052309
AML2T	22531536	19058637 (84.5%)	2445007 (10.9%)	631044 (2.8%)	215123 (1%)	181725 (0.8%)	27990455
AML2R	22529573	19057120 (84.5%)	2444698 (10.9%)	630949 (2.8%)	215116 (1%)	181690 (0.8%)	27987801

The table reports the total number CpG groups inferred by Nanopolish in each sample (CpG Groups). Columns from 3 to 7 report the number CpG groups containing 1 (1 CpG site), 2 (2 CpG sites), 3 (3 CpG sites), 4 (4 CpG sites) and more than five (≥5 CpG sites) CpG sites. Last column reports the cumulative number of CpG sites in all CpG groups.

Table 3: DMRs annotation statistics

Sample	# DMRs	# DHS	# TFBS	Enhancers	# Genes	# CGIs	# NoCGIs	# Hyper-CGIs	# Hyper-NoCGIs	# Hypo-CGIs	Hypo-NoCGIs
UD5	1269	1206 (95%)	1052 (83%)	199 (16%)	919 (72%)	325 (26%)	325 (74%)	944 (96%)	312 (4%)	13 (54%)	510 (46%)
UD10	1149	1111 (97%)	1017 (89%)	118 (10%)	822 (72%)	744 (65%)	744 (35%)	405 (97%)	718 (3%)	26 (63%)	254 (37%)
AML2	1314	1257 (96%)	1155 (88%)	207 (16%)	1014 (77%)	596 (45%)	596 (55%)	718 (98%)	586 (2%)	10 (86%)	620 (14%)

The table reports statistics about DMRs annotation. For each sample (UD5, UD10 and AML2) are reported the total number of DMRs (# DMRs), the number of DMRs overlapping DHS (# DHS), TFBS (# TFBS), annotated genes (# Genes), CpG islands (# CGIs), and sparse CpGs (# NoCGIs, DMRs that do not overlap any CGIs). The last four columns report the number of number of hyper- and hypo-methylated DMRs overlapping a not overlapping CGIs (# Hyper-CGIs, # Hyper-NoCGIs, # Hypo-CGIs, # Hypo-NoCGIs). Percentages in brackets show proportion with respect to total DMRs. For the last four columns percentages report proportion with respect to total hyper- (# Hyper-CGIs, # Hyper-NoCGIs) and hypo-methylated (# Hypo-CGIs, # Hypo-NoCGIs) DMRs.

Table 4: Differentially expressed Genes in the three AML patients.

Sample	DEGs	Over-expressed Genes	Under-Expressed Genes
UD5	4,677	2,044	1,953
UD10	3,997	2,890	1,787
AML2	1,759	495	1,264

The table reports the total number of differentially expressed genes (DEGs), over-expressed and under-expressed genes identified by DESeq2 (adjusted p-value<0.05 and absolute $log_2FC > 0.5$) on the three AML samples.

Table 5: Differentially methylated and Differentially expressed Genes in the three AML patients.

Sample	Hyper-Over	Hypo-Over	Hyper-Under	Hypo-Under
UD5	97 (5%)	40 (2%)	100 (5%)	39 (2%)
UD10	33 (1%)	22 (1%)	51 (3%)	17 (1%)
AML2	19 (4%)	5 (1%)	75 (6%)	5 (0%)

The table reports the total number of Differentially methylated and Differentially expressed Genes (DM-DEGs) for the three AML samples. Statistics are reported for the four methylation-expression combination: hyper-methylation and over-expression (Hyper-Over), hypo-methylation and over-expression (Hypo-Over), hyper-methylation and under-expression (Hyper-Under), hypo-methylation and under-expression (Hypo-Under). For each category, percentages in brackets report the proportions of DM-DEGs with respect to total number of over- or under-expressed genes.

Table 6: Table of direct TF–DNA interactions between under-expressed DM-DE-TFs hyper-methylated at sparse CpGs of gene-bodies and DE-TGs.

Sample	DM-DE-TF	TF–DNA	DE-TGs
UD5	TFDP1	6	6
UD5	CUX1	101	234
UD5	ARID3A	X	3
UD5	ETV6	5	6
UD5	NCOR2	X	20
UD5	RERE	X	6
UD10	RARA	39	44
UD10	BRF1	X	5
AML2	ZBTB16	X	15
AML2	RREB1	X	48
AML2	CUX1	60	110
AML2	GTF2I	X	4
AML2	NCOR2	X	12

Columns report the Sample name (Sample), the gene symbol of the DM-DE-TFs (Gene), the number of DE-TGs with direct TF–DNA interactions in UniBind (TF–DNA), the number of all the DE-TGs regulated by the DM-DE-TF (DE-TGs). X means that the DM-DE-TF is not present in UniBind.

Table 7: Relapse-specific SVs detected by NanomonSV in the three AML pairs.

Chr	Start	End	Size	Type	RS	TS	Sample	Gene	Visual Inspection	Tool	Figure
3	116259740	116486402	235795	Del	7/19 (0.36)	0/77	UD5	TUSC7 (All)	Relapse-specific	Samplot	1
3	76182548	76182814	266	Del	4/32 (0.12)	0/28	UD5	Yes ROBO2 (Intron)	Relapse-specific	IGV	2
3	116604969	116631956	26987	Dup	9/63 (0.14)	0/72	UD5		Relapse-specific	Samplot	3
7	115992315	115994264	1949	Del	9/40 (0.22)	0/38	UD5	CAV2 (Exon)	Relapse-specific	IGV	4
16	50350527	50352587	2060	Del	9/34 (0.26)	0/26	UD5	ADCY7 (Exon)	Relapse-specific	IGV	5
6	32454684	32478662	23978	Del	5/25 (0.2)	0/36	UD10		Not Relapse-specific	IGV	6
13	35684164	35684406	242	Del	4/25 (0.16)	0/28	UD10	NBEA (Intron)	Relapse-specific	IGV	7
17	20707099	20707205	106	Del	3/26 (0.11)	0/24	UD10		Relapse-specific	IGV	8
19	43718061	43718371	310	Del	4/13 (0.3)	0/10	UD10	PSG9 (Intron)	Relapse-specific	IGV	9
4	140244927	143675814	3430887	Del	7/34 (0.2)	0/51	AML2	SETD7, MGST2, MAML3, SCOC, ELMOD2, UCP1, ZNF330, IL15, INPP4B (All)	Relapse-specific	Samplot	10
14	106215508	106215781	273	Del	3/21 (0.14)	0/21	AML2	IGHM (Intron)	Relapse-specific	IGV	11
19	23647827	23648154	327	Del	4/19 (0.21)	0/19	AML2		Relapse-specific	IGV	12
19	23655167	23655537	370	Del	3/15 (0.2)	0/16	AML2		Relapse-specific	IGV	13

Columns report the genomic coordinates (Chr, Start and End, with respect to hg19), the size (in bp), the type of SV (Del, Dup), the number of signatures supporting the SV and the total number of reads with reference allele in the relapsed sample (RS, signature number are separated by /, between brackets is reported cellular fraction), the number of signatures supporting the SV and the total number of reads with reference allele in the tumor sample (TS, signature number are separated by /), the sample name, the results obtained by NanomonSV in the identification of the SV ,the result of visual inspection (Relapse-specific if SV signatures are present only in relapse sample, Not Relapse-specific if SV signatures are present in both relapsed and tumor samples), the visualization tool used for visual inspection (Tool, Samplot or IGV) and the figure number in which are reported the results of visual inspection in supplemental file 4.

Table 8: Gene Expression inferred by DESeq2 for all statistically significant DEGs affected by SVs.

Sample	Gene	Chr	Start	End	$\text{Log}_2 FC$	FDR
UD5	ADCY7	chr16	50350527	50352587	0.35	$1 \cdot 10^{-2}$
AML2	ZNF330	chr4	140244927	143675816	-0.75	$1 \cdot 10^{-13}$
AML2	MGST2	chr4	140244927	143675816	-0.46	$3 \cdot 10^{-12}$
AML2	MAML3	chr4	140244927	143675816	-1.13	$5 \cdot 10^{-33}$
AML2	SCOC	chr4	140244927	143675816	-0.84	$1 \cdot 10^{-16}$
AML2	NAA15	chr4	140244927	143675816	-1.32	$4 \cdot 10^{-73}$
AML2	INPP4B	chr4	140244927	143675816	-1.35	$2 \cdot 10^{-81}$

Columns report the Sample name (Sample), the gene symbol (Gene), the coordinates of the SV (Chr, Start, End), the log2 fold change inferred by DESeq2 ($Log_2 FC$) and the adjusted p-value (FDR).

Table 9: Variants identified by WES in the three AML samples.

Sample	Disease Phase	Gene	Chr	Start	End	Ref	Alt	Variant Type	T_{VAF}	R_{VAF}
UD5	T	MYPOP	19	46394248	46394248	C	T	nonsynonymous SNV	12.07	-
UD5	R/T	BRINP2	1	177250213	177250213	G	A	nonsynonymous SNV	44.63	45.38
UD5	R/T	ZC3HC1	7	129679315	129679315	C	A	nonsynonymous SNV	38.85	45.40
UD5	R/T	FILIP1L	3	99648839	99648839	T	C	nonsynonymous SNV	46.46	49.00
UD5	R/T	VN1R2	19	53762305	53762305	T	G	nonsynonymous SNV	43.14	42.52
UD5	R/T	CEBPA	19	33792821	33792821	-	C	frameshift insertion	11.11	45.83
UD5	R/T	DSPP	4	88536863	88536880	AATAGTAGTGACAGCAGC	-	nonframeshift deletion	70.49	89.47
UD5	R/T	NMNAT1	1	10035794	10035794	-	T	frameshift insertion	39.07	43.31
UD5	R/T	NPM1	5	170837543	170837543	-	TCTG	frameshift insertion	47.06	45.45
UD5	R/T	PRR21	2	240982109	240982136	GAAGGGCCGTGGGTGAAGAGGCATGGAT	-	frameshift deletion	43.48	42.86
UD5	R	SCIN	7	12691477	12691477	G	A	stopgain	-	10.00
UD5	R	SLC44A1	9	108118642	108118642	G	A	nonsynonymous SNV	-	11.11
UD5	R	KRT23	17	39081765	39081765	T	A	nonsynonymous SNV	-	20.00
UD5	R	SLC2A3	12	8084037	8084037	C	A	nonsynonymous SNV	-	21.25
UD5	R	ZNF34	8	145998937	145998937	T	C	nonsynonymous SNV	-	25.00
UD5	R	GPR123	10	134942360	134942360	G	C	nonsynonymous SNV	-	30.60
UD5	R	ENDOD1	11	94861922	94861922	C	A	nonsynonymous SNV	-	30.88
UD5	R	CTNNA2	2	80136765	80136765	C	T	nonsynonymous SNV	-	44.83
UD5	R	OR8B8	11	124310617	124310617	C	T	nonsynonymous SNV	-	18.62
UD5	R	TTN	2	179584137	179584137	C	T	nonsynonymous SNV	-	15.15
UD5	R	FLT3	13	28608268	28608268	-	TCTCTGAAATCAACGTAGAAGTACTCATTATCTGAGGAGCCGGTCACCTGTACCATCTGA	nonframeshift insertion	-	13.30
UD10	R/T	NRAS	1	115256530	115256530	G	T	nonsynonymous SNV	41.90	40.00
UD10	R/T	YLPM1	14	75248804	75248804	T	A	nonsynonymous SNV	40.00	45.10
UD10	R/T	KRT8	12	53298675	53298675	A	C	nonsynonymous SNV	13.04	11.76
UD10	R/T	CTNNA3	10	68526128	68526128	G	A	nonsynonymous SNV	42.00	44.78
UD10	R/T	ISLR	15	74468241	74468241	G	A	nonsynonymous SNV	42.96	41.03
UD10	R/T	SYT10	12	33559867	33559867	G	A	stopgain	48.33	46.67
UD10	R	TMEM132D	12	129566312	129566312	T	G	nonsynonymous SNV	2.38	10.00
UD10	R	IVL	1	152883532	152883532	T	G	nonsynonymous SNV	3.85	19.51
UD10	R	KDR	4	55970945	55970945	A	G	nonsynonymous SNV	-	30.97
UD10	R	TRPC4	13	38320085	38320085	G	A	nonsynonymous SNV	-	34.90
UD10	R	DMKN	19	36002421	36002421	-	CTGCTGCTG	nonframeshift insertion	-	57.14
UD10	R	DSPP	4	88537205	88537213	GACAGCAGT	-	nonframeshift deletion	-	78.95
AML2	T	GOLGA6L9	chr15	82434465	82434465	C	T	stopgain	11.50	-
AML2	T	PDPR	chr16	70144498	70144498	A	T	nonsynonymous SNV	35.70	-
AML2	R/T	TCERG1L	chr10	131260421	131260421	C	A	nonsynonymous SNV	59.00	55.40
AML2	R/T	NUAK1	chr12	106066930	106066930	G	T	nonsynonymous SNV	48.30	44.70
AML2	R/T	PCDH8	chr13	52847542	52847542	G	A	nonsynonymous SNV	47.40	49.50
AML2	R/T	KIAA1755	chr20	38217300	38217300	G	A	nonsynonymous SNV	45.20	45.80
AML2	R/T	TBXAS1	chr7	139953419	139953419	C	T	nonsynonymous SNV	44.90	48.50
AML2	R/T	RNF20	chr9	101554106	101554106	G	A	splice$_{s}ite$	46.60	44.20
AML2	R/T	STAG2	chrX	124045321	124045321	T	A	stopgain	48.20	45.70
AML2	R	POTEI	chr2	130463831	130463831	G	C	nonsynonymous SNV	-	47.90
AML2	R	ANAPC4	chr4	25418249	25418259	GTAATAAGCCT	-	frameshift deletion	-	32.80

Columns report the sample name (Sample), the Disease Phase in which the variant has been detected (T for primary tumor, R for relapse, R/T for common to tumor and relapse), the gene affected by the variant (Gene), the genomic coordinates (Chr, Start and End, with respect to hg19), the reference and alternative alleles that describe the variant (Ref and Alt), the functional effect (Variant Type) and the variant allele fraction in primary tumor (T_{VAF}) and in relapsed samples (R_{VAF}).

Table 10: Differential Expression inferred by DESeq2 for all the components of the methylation machinery.

Gene	$\text{Log}_2 FC$	FDR	Sample
DNMT1	-0.45	$8{\cdot}10^{-5}$	UD5
DNMT1	-0.51	$2{\cdot}10^{-7}$	UD10
DNMT1	-0.54	$2{\cdot}10^{-40}$	AML2
TET1	2.86	$4{\cdot}10^{-18}$	UD5
TET1	2.11	$3{\cdot}10^{-19}$	UD10
TET1	-0.47	0.003	AML2
TET2	0.17	0.48	UD5
TET2	-1.03	$1{\cdot}10^{-8}$	UD10
TET2	-1.27	$4{\cdot}10^{-78}$	AML2
TET3	-0.62	$2{\cdot}10^{-5}$	UD5
TET3	-0.68	$1{\cdot}10^{-7}$	UD10
TET3	-0.89	$3{\cdot}10^{-17}$	AML2
DNMT3A	0.016	0.92	UD5
DNMT3A	0.88	$3{\cdot}10^{-16}$	UD10
DNMT3A	-0.42	$4{\cdot}10^{-10}$	AML2
DNMT3B	1.52	$1{\cdot}10^{-30}$	UD5
DNMT3B	2.59	$2{\cdot}10^{-49}$	UD10
DNMT3B	0.37	0.1	AML2

Columns report the gene name, the log2 fold change inferred by DESeq2 (Log_2FC), the adjusted p-value (FDR) and the sample name (Sample) .

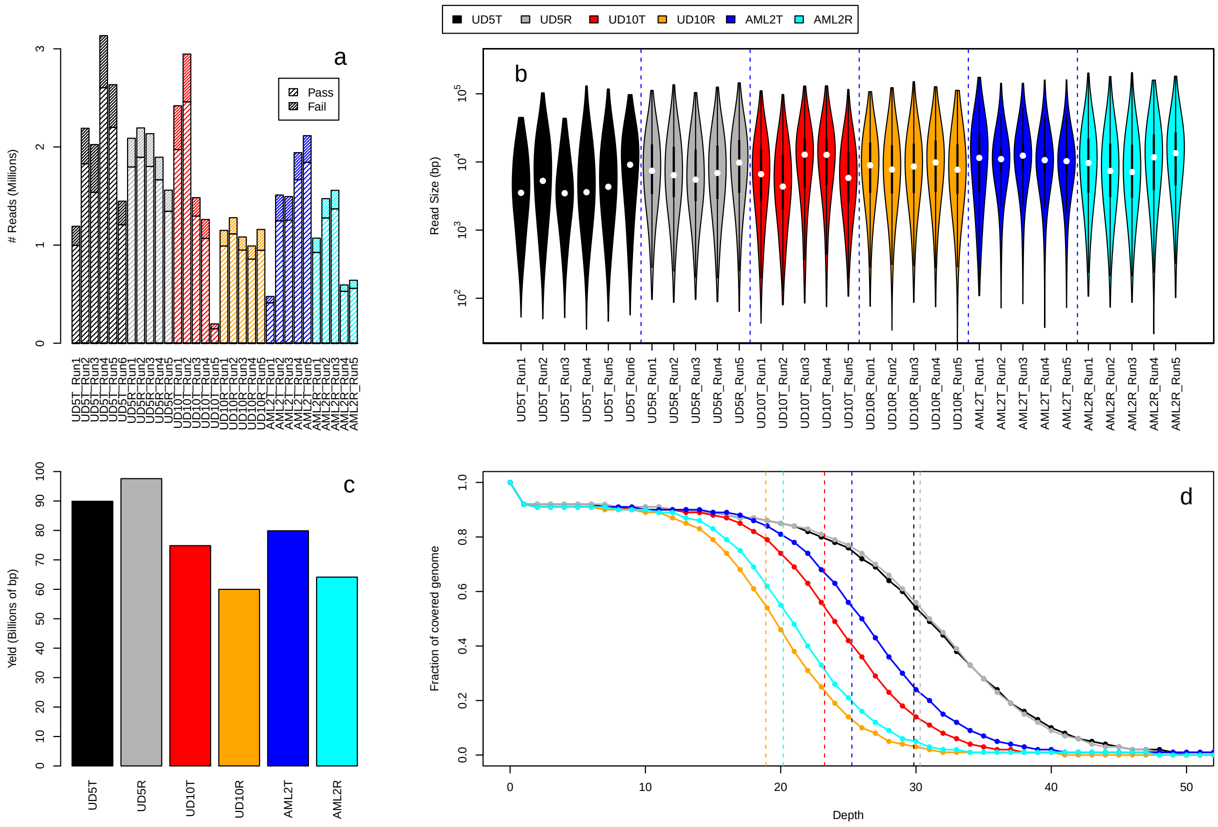

Figure 1: Sequencing Statistics and Structural variants. Panel (a) shows the total number of reads (Pass and Fail) generated by the 31 sequencing run performed on the six AML samples. Each sequencing run generated between 250000 and 3.2 millions total reads, with a fraction of low-quality reads (fail) around 15-25% that were discarded by the base-caller. The violin plots of panel (b) report the sequence size distribution of pass reads for each sequencing run. Pass read size distributions were consistent across all the runs with a mean read length that ranges between a minimum of 5857 bp (UD5T_Run3) to a maximum of 17584 bp (AML2R_Run5) For each sample, panel (c) shows the total sequencing throughput of pass reads. Panel (d) reports the fraction of genome as a function of sequencing coverage obtained by minimap2. Vertical dotted lines indicate the average coverage for each sequenced sample: 30x (UD5T), 30x (UD5R), 24x (UD10T), 19x (UD10R), 25x (AML2T) and 20x (AML2R).

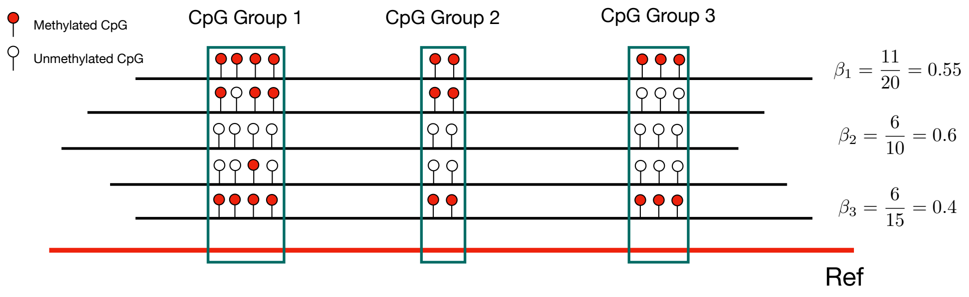

Figure 2: β values calculation. Nanopolish is based on a hidden Markov model that assigns a log-likelihood ratio to a group of nearby CpG sites that share the same methylation level in each site within the group. To estimate the methylation level (β), for each CpG group identified by Nanopolish, we calculated the ratio between the total number of CpG sites predicted as methylated divided by the total number of CpG sites in the group. In figure are reported β calculation of three CpG groups inferred by Nanopolish. Red line indicates the reference genome, black lines indicate the reads, red (white) dots indicate methylated (unmethylated) CpGs inferred by Nanopolish.

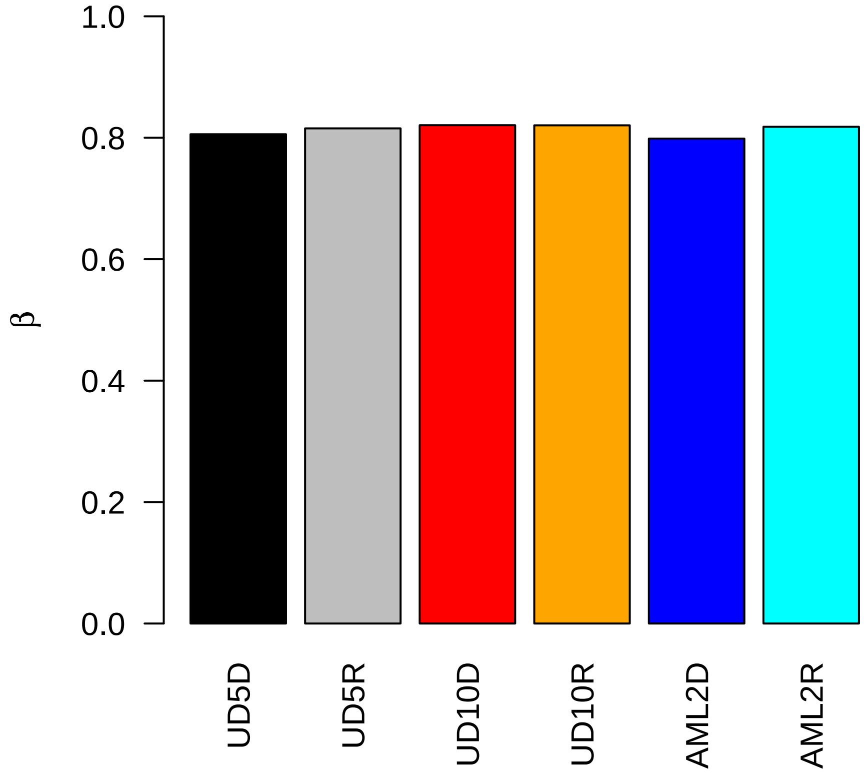

Figure 3: Barplot reports the average β values of all CpG sites for each sample.

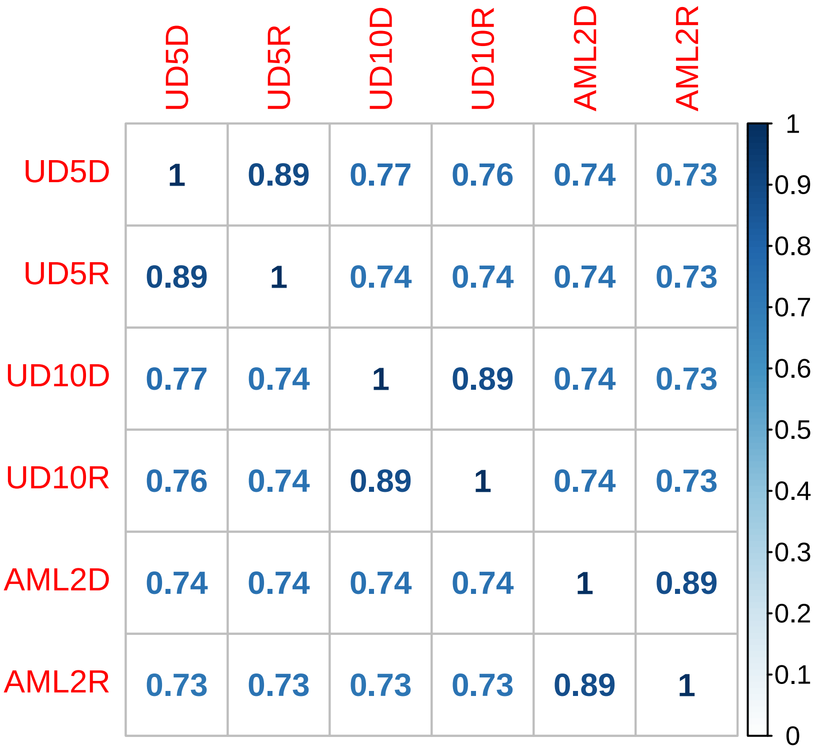

Figure 4: Corrplot reports the correlation of β values of all CpG sites between pair of samples.

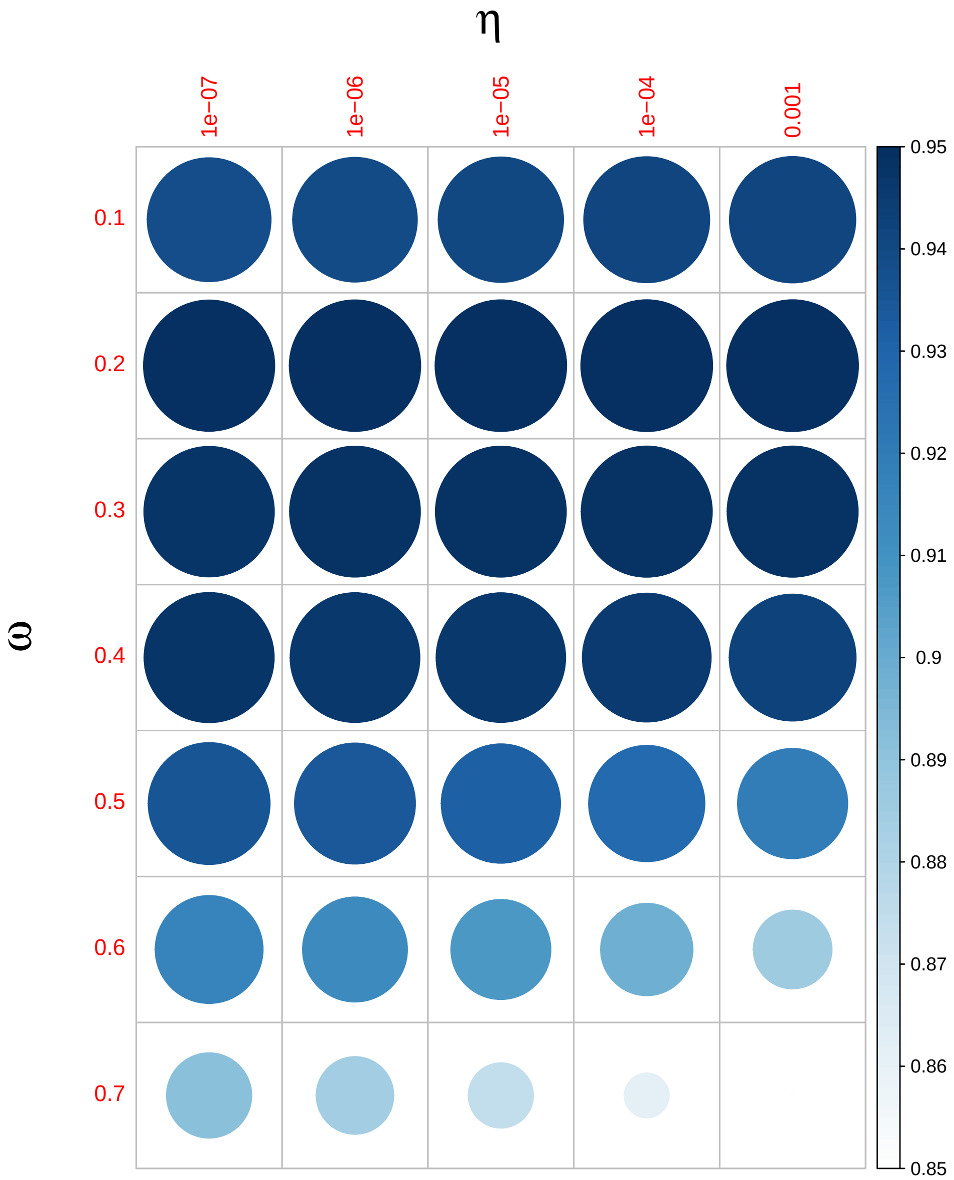

Figure 5: PoreMeth and parameter settings on synthetic data. The dotplot shows the F-score (harmonic mean of the precision and recall) obtained by PoreMeth on the analysis of synthetic data with background 1, for different combinations of ω and θ.

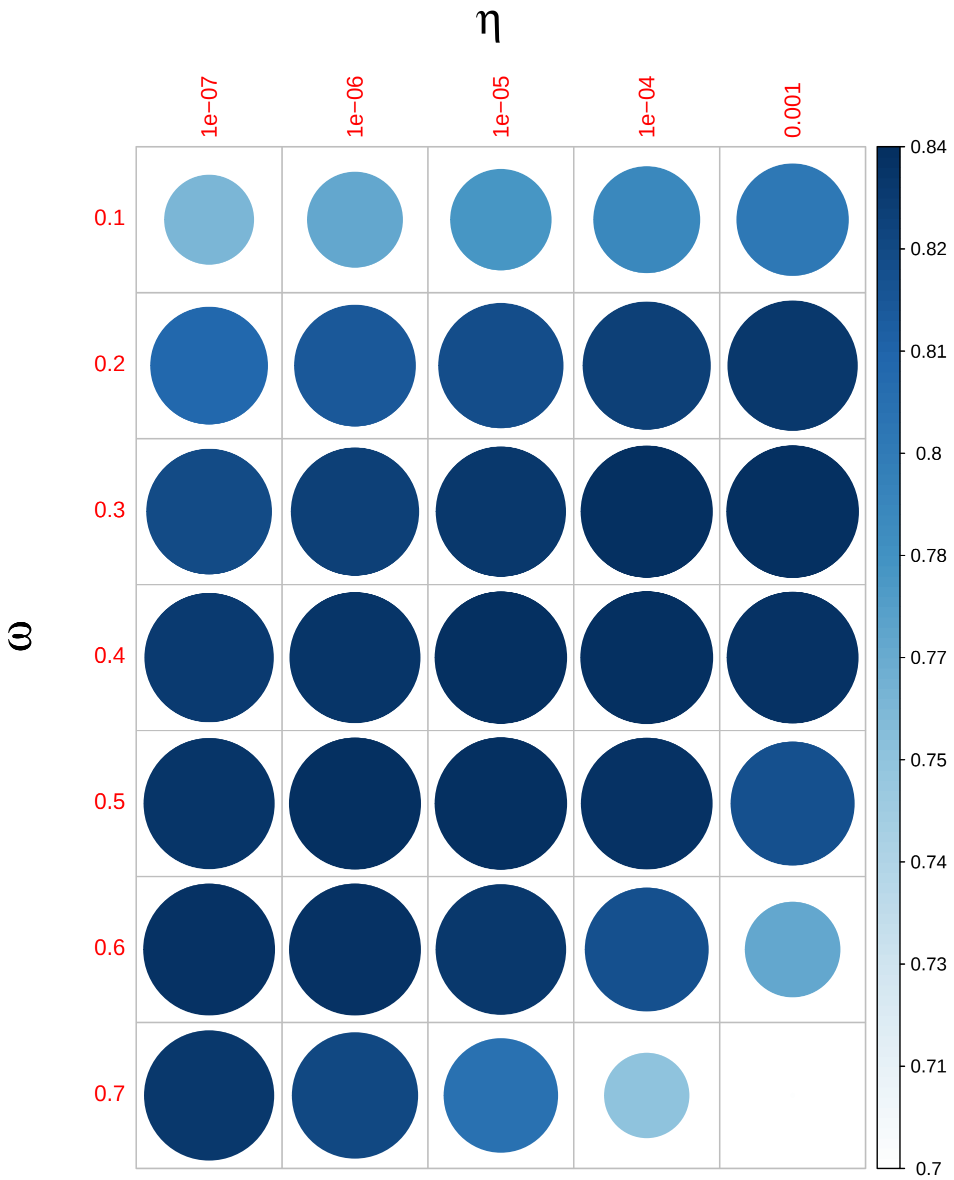

Figure 6: PoreMeth and parameter settings on synthetic data. The dotplot shows the F-score (harmonic mean of the precision and recall) obtained by PoreMeth on the analysis of synthetic data with background 2, for different combinations of ω and θ.

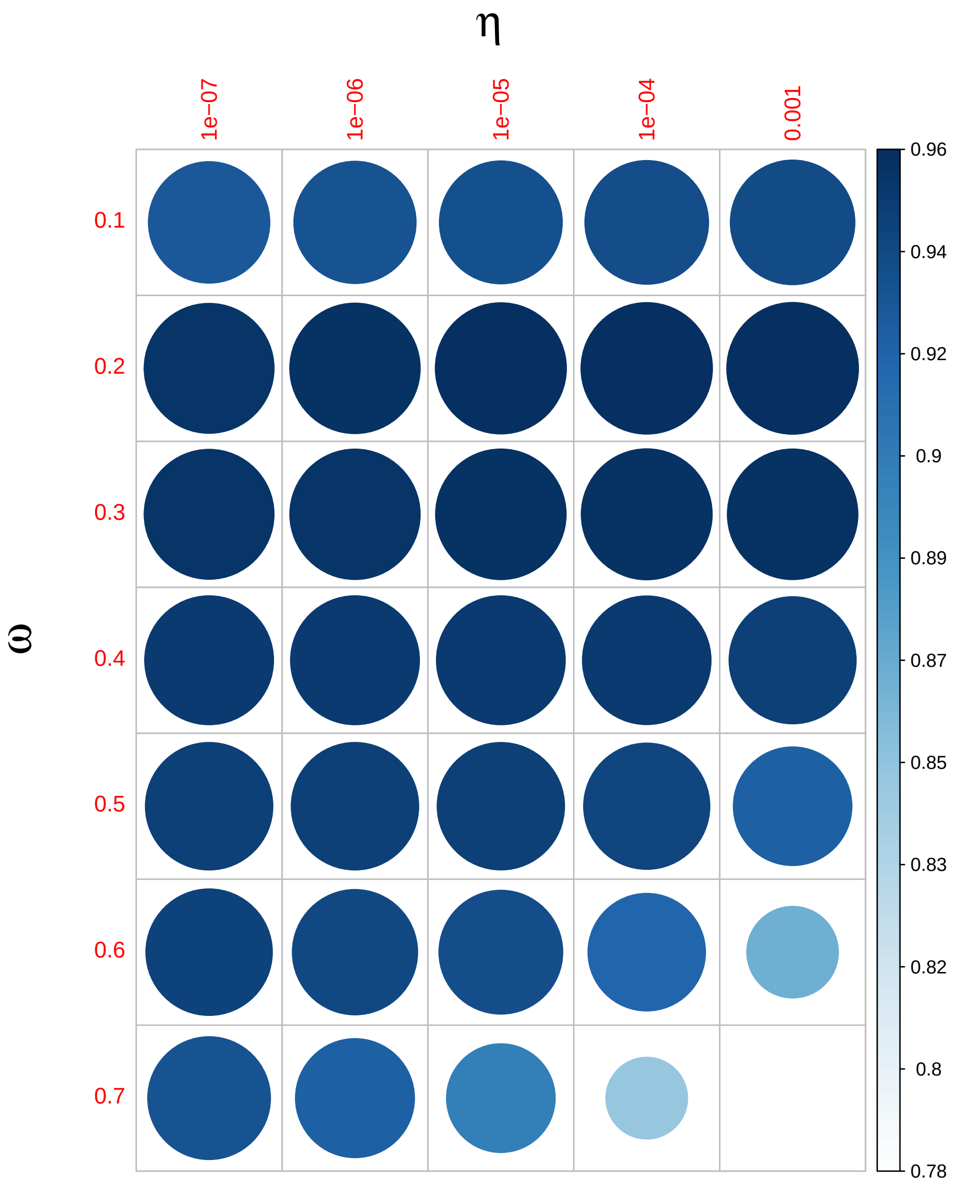

Figure 7: PoreMeth and parameter settings on synthetic data. The dotplot shows the F-score (harmonic mean of the precision and recall) obtained by PoreMeth on the analysis of synthetic data with degrees of methylation of class 1, for different combinations of ω and θ.

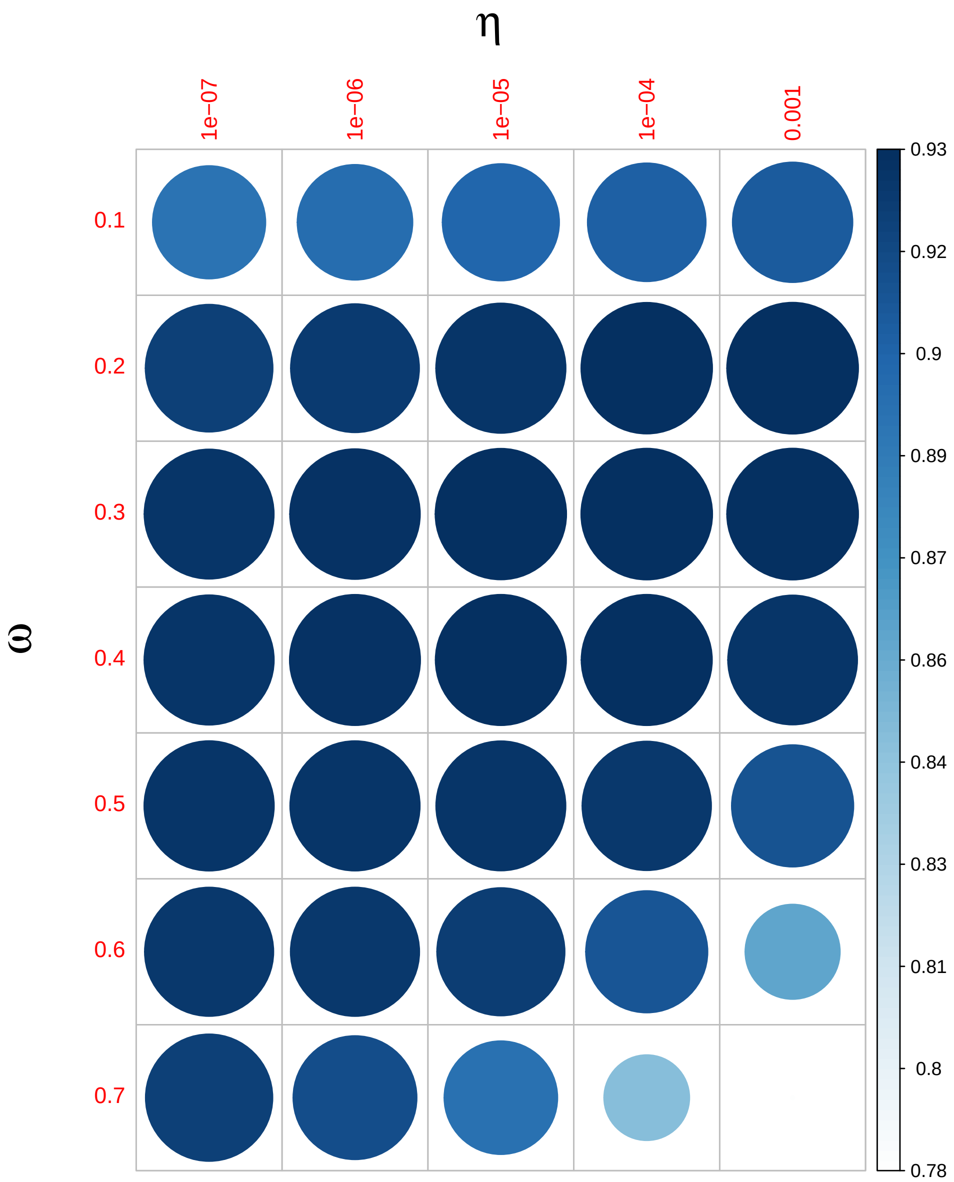

Figure 8: PoreMeth and parameter settings on synthetic data. The dotplot shows the F-score (harmonic mean of the precision and recall) obtained by PoreMeth on the analysis of synthetic data with degrees of methylation of class 2, for different combinations of ω and θ.

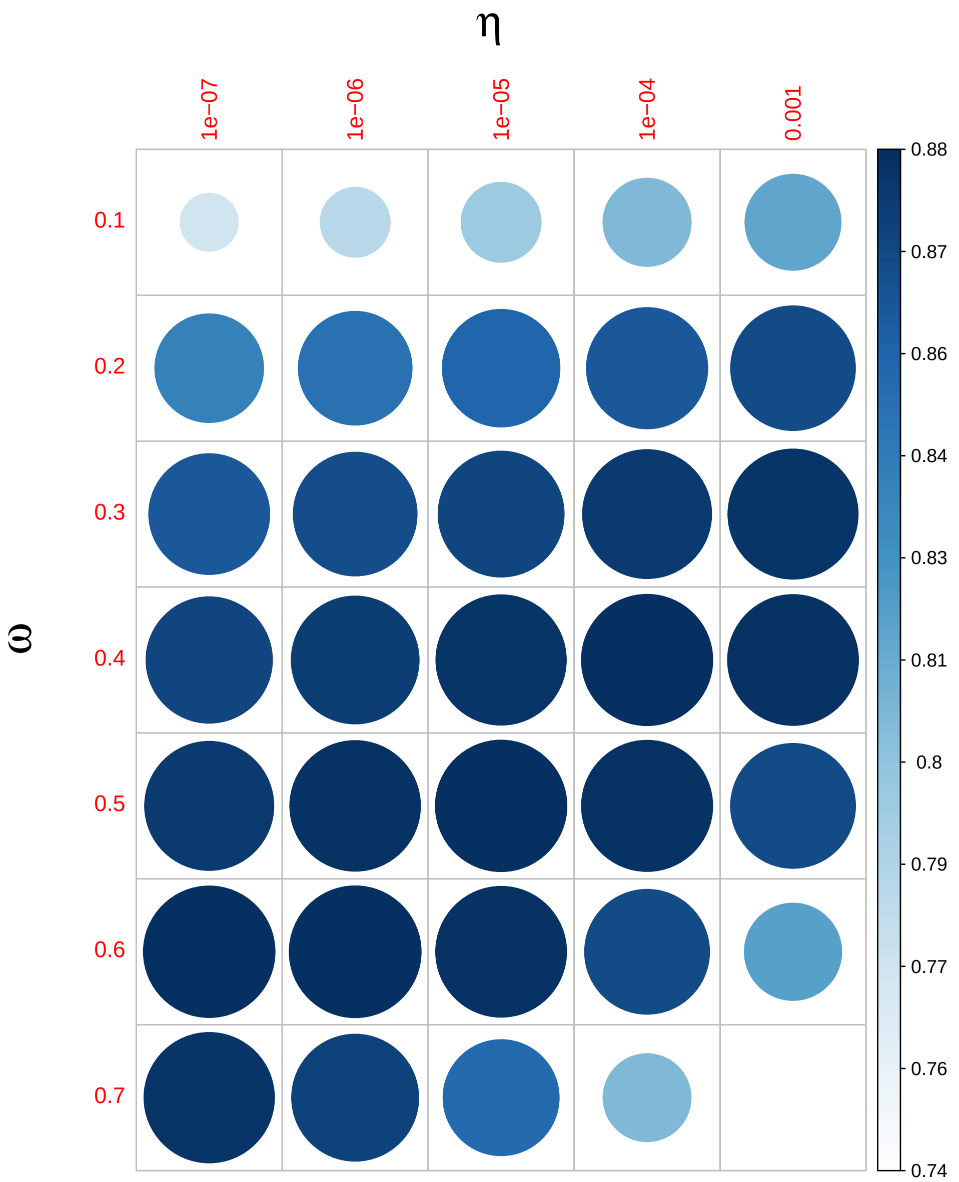

Figure 9: PoreMeth and parameter settings on synthetic data. The dotplot shows the F-score (harmonic mean of the precision and recall) obtained by PoreMeth on the analysis of synthetic data with degrees of methylation of class 3, for different combinations of ω and θ.

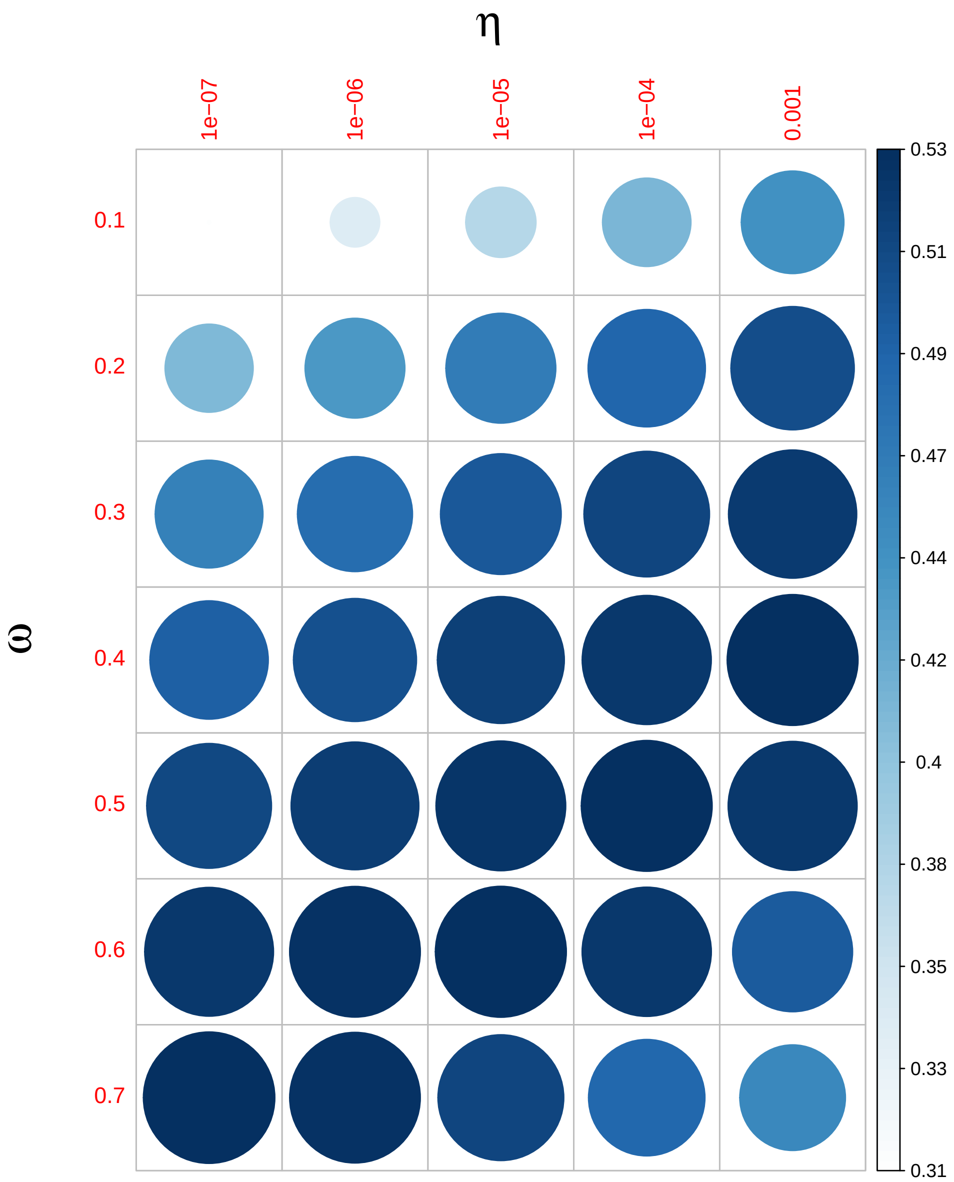

Figure 10: PoreMeth and parameter settings on synthetic data. The dotplot shows the F-score (harmonic mean of the precision and recall) obtained by PoreMeth on the analysis of synthetic data with degrees of methylation of class 4, for different combinations of ω and θ.

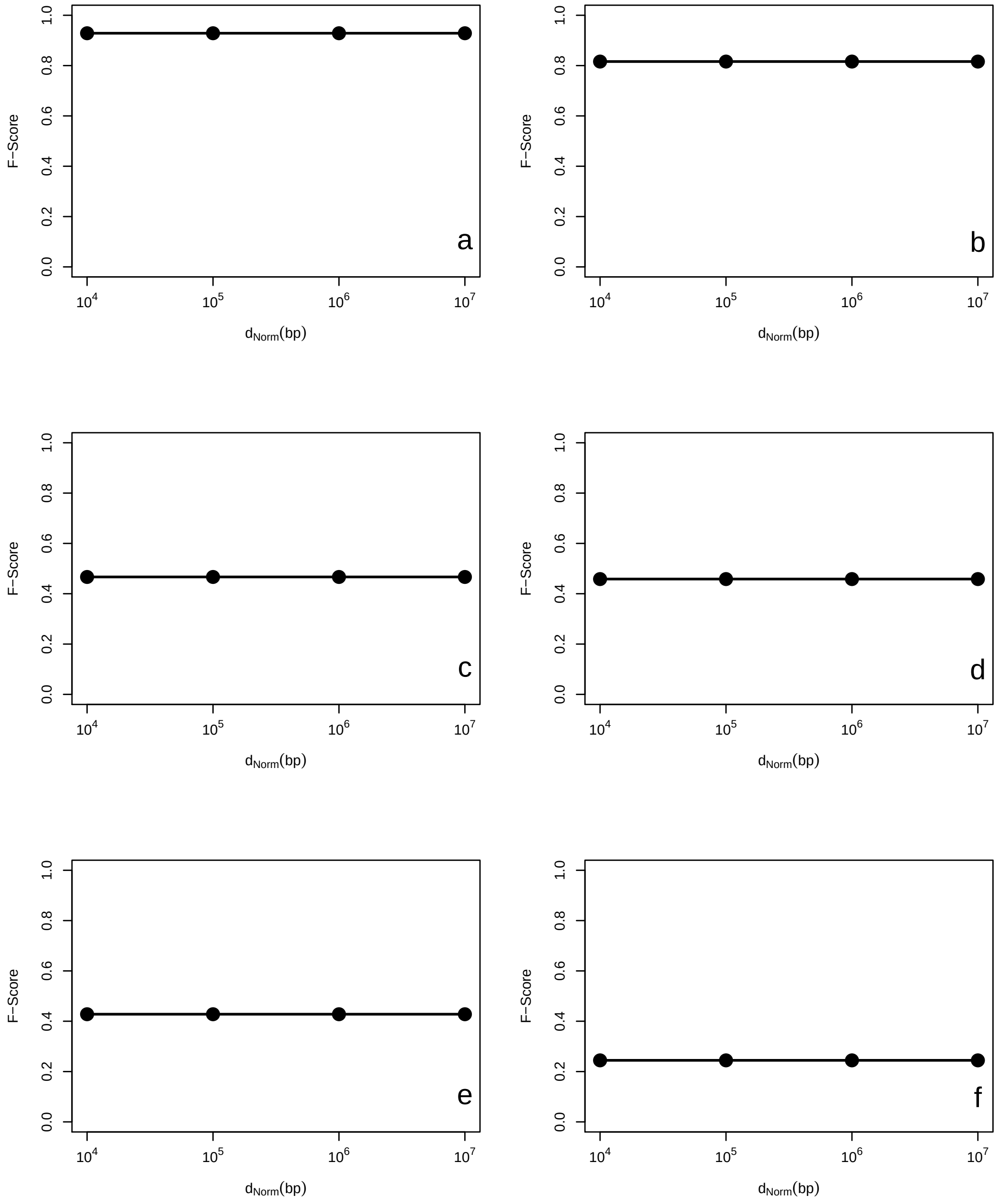

Figure 11: PoreMeth and parameter settings on synthetic data. The plot shows the F-score (harmonic mean of the precision and recall) obtained by PoreMeth on the analysis of synthetic data with background 1-2 (panels a-b) and degrees of methylation of class 1-4 (panels c-f), for different values of d_{Norm}.

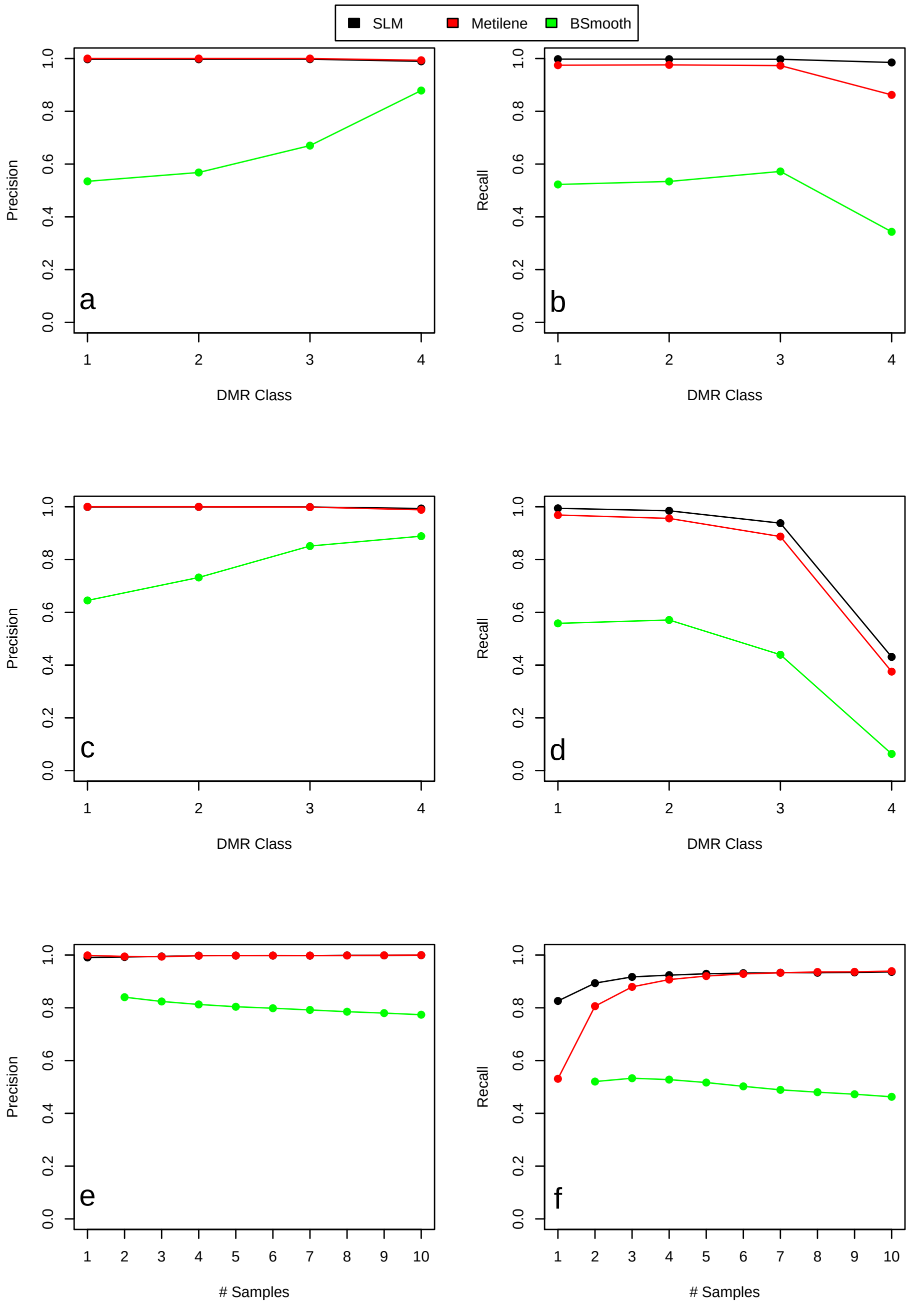

Figure 12: Comparison between PoreMeth, Metilene and BSmooth.The plot shows precision and recall obtained by the three tools as a function of differential methylation class (a-d) and as a function of analyzed samples (e-f). Panels a-b report the results for background 1 (low noise), while panels c-d for background 2 (high noise).

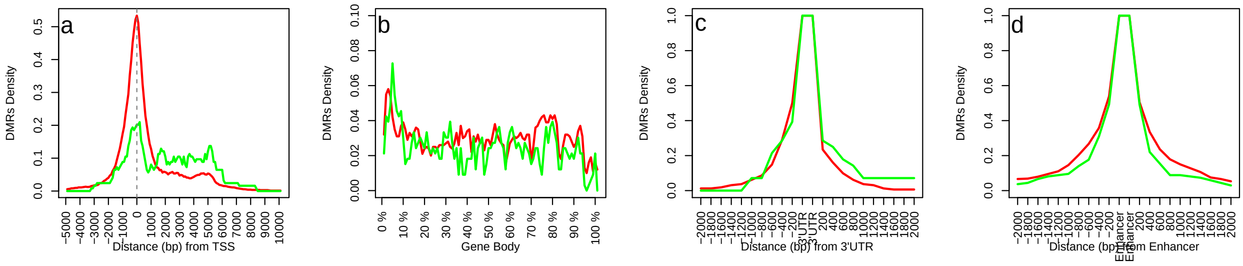

Figure 13: DMRs distribution across genomic. The four panels report the distribution of DMRs at 5' regulatory region (a), at gene-body (b), at 3'UTR (c) and at enhancers (d). Red lines for hyper-methylation and green lines for hypo-methylation.

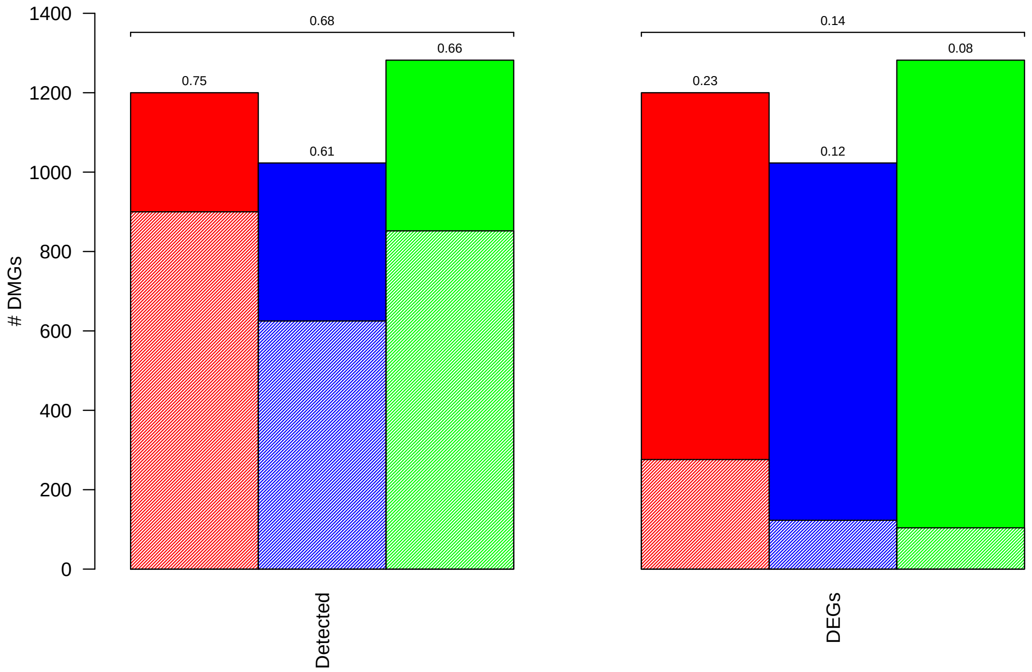

Figure 14: Differentially-methylated genes (DMGs) and RNASeq results on the three AML pairs. Left barplots report the total number of DMGs (solid bars) and the numbers of DMGs with detectable RNASeq signals (textured bars). Right barplots report the total number of DMGs (solid bars) and the number of DMGs that are also differentially expressed (textured bars, adjusted p-value< 0.05 and absolute log2FC > 0.5) in the three AML pairs. Numbers above bars show the percentage of DMGs with detectable RNASeq signals (left) and DM-DEGs (right).

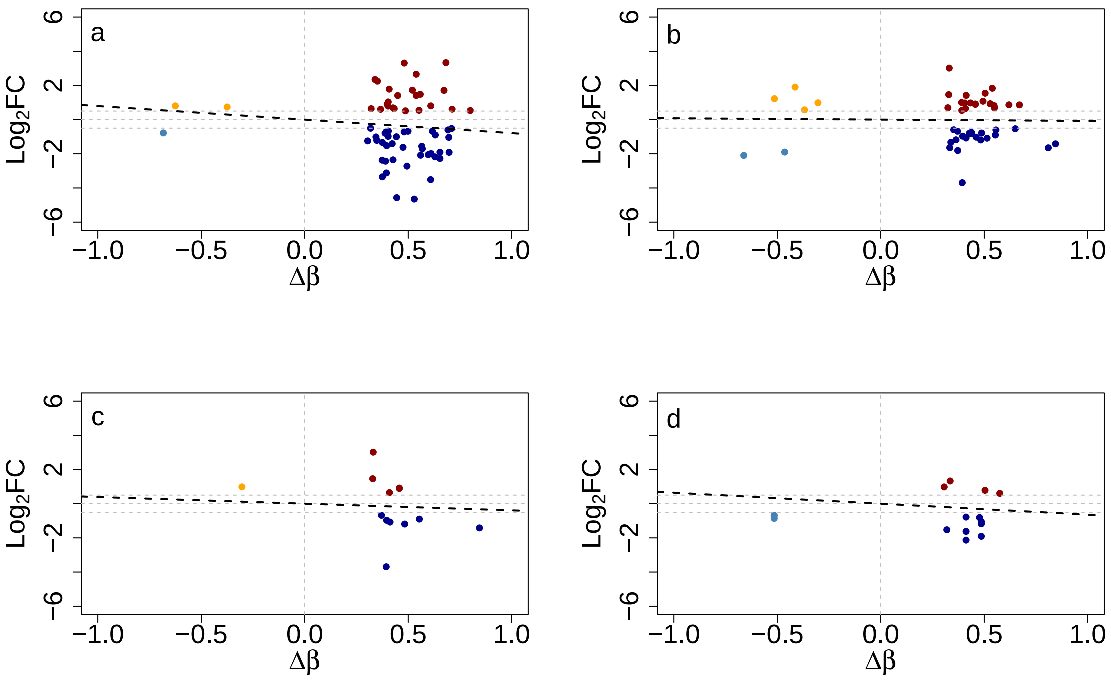

Figure 15: Correlation between Log_2FC and $\Delta\beta$ values in genomic elements that overlap CpG islands. Panels show the correlation between Log_2FC and $\Delta\beta$ values of DEG with differentially methylated regulatory element (a), gene body (b), 3'UTR (c) and enhancer (d). All the DMRs taken into consideration in this analysis overlap with a CpG island. Dotted line represent the fitted linear regression curve.

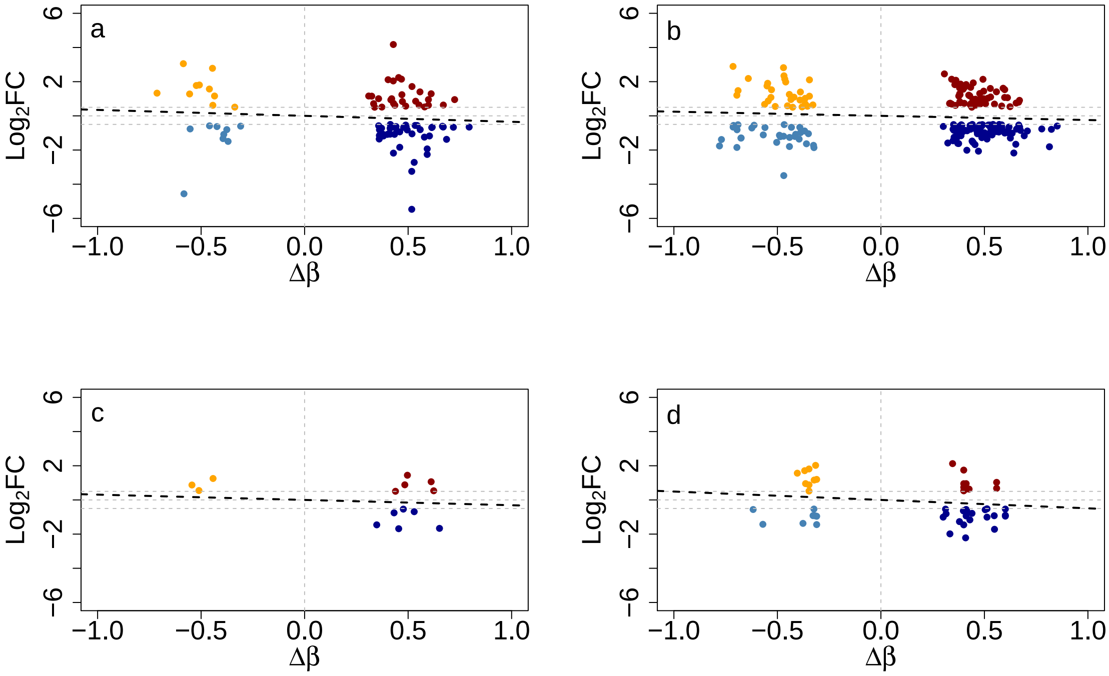

Figure 16: Correlation between Log_2FC and $\Delta\beta$ values in genomic elements that do not overlap CpG Islands. Panels show the correlation between Log_2FC and $\Delta\beta$ values of DEG with differentially methylated regulatory element (a), gene body (b), 3'UTR (c) and enhancer (d). All the DMRs taken into consideration in this analysis do not overlap with a CpG island. Dotted line represent the fitted linear regression curve.

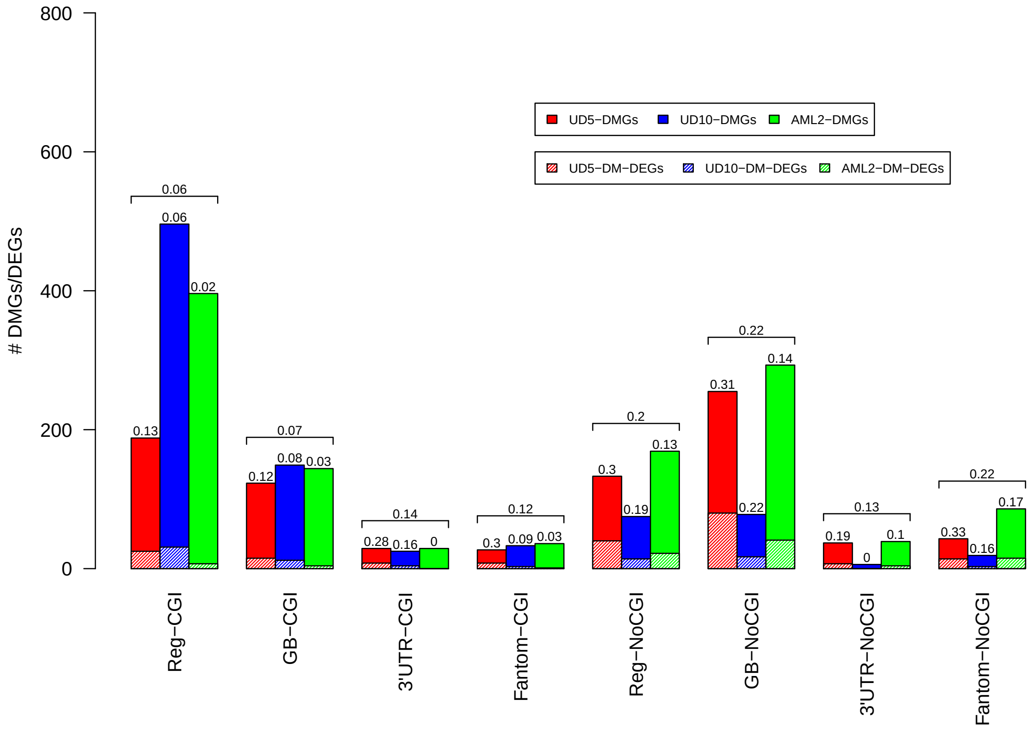

Figure 17: Hyper-methylated genes and differentially expressed genes on the three AML pairs. Barplots report the total number of genes hyper-methylated (solid bars) genes and the numbers of hyper-methylated genes that are also DEGs (textured bars) for each genic element (regulatory element (Reg), gene body (GB), 3'UTR and Fantom) in CGIs and in sparse CpG regions (No-CGI). Numbers above bars show the percentage of Hyper-methylated genes that are also DEGs. Horizontal brackets above each group of three bars summarize average percentages within the three samples.

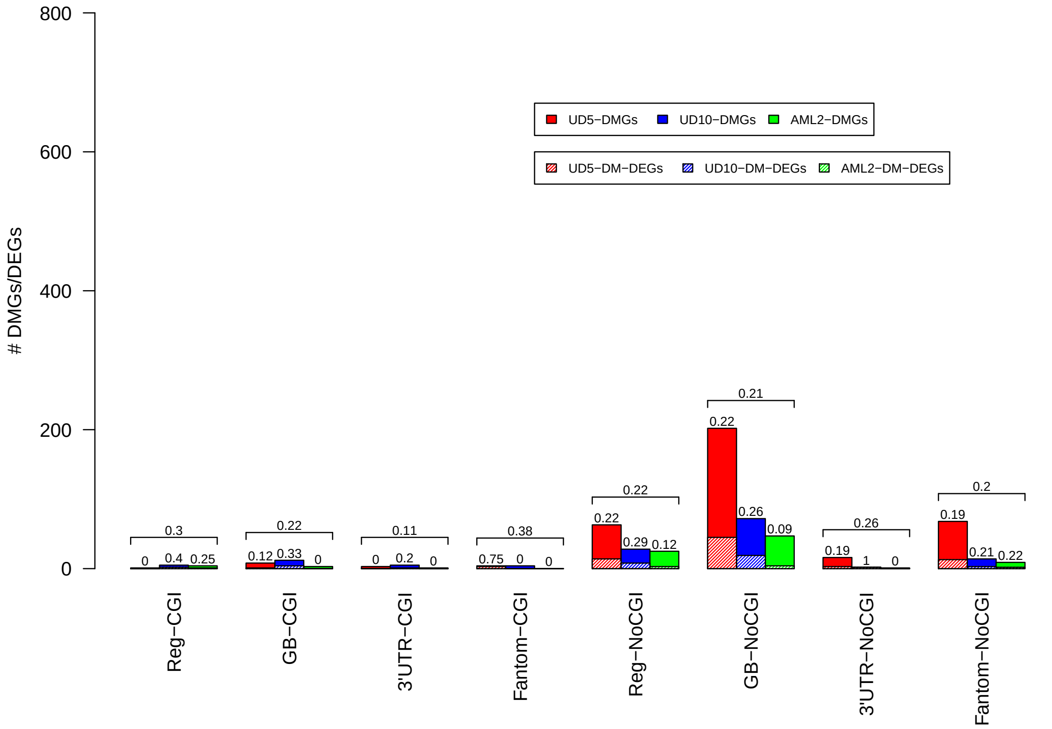

Figure 18: Hypo-methylated genes and differentially expressed genes on the three AML pairs. Barplots report the total number of hypo-methylated (solid bars) genes and the numbers of hypo-methylated genes that are also DEGs (textured bars) for each genic element (regulatory element (Reg), gene body (GB), 3'UTR and Fantom) in CGIs and in sparse CpG regions (No-CGI). Numbers above bars show the percentage of hypo-methylated genes that are also DEGs. Horizontal brackets above each group of three bars summarize average percentages within the three samples.

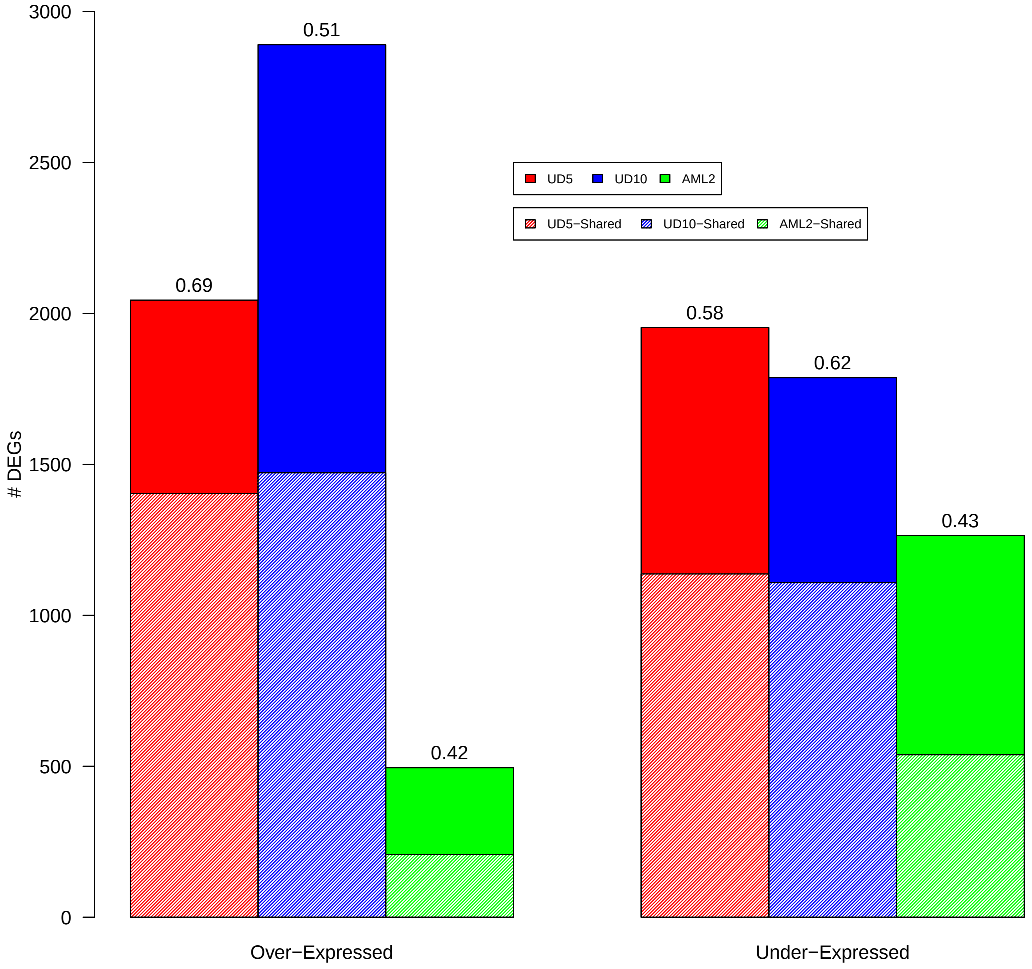

Figure 19: Differentially expressed (DEGs) identified by DESeq2 in the three AML pairs. Barplots report the total number of over-expressed (left) and under-expressed (right) genes detected by DESeq2 (adjusted p-value< 0.05 and absolute log2FC > 0.5) in the three AML pairs. Textured bars show the number of DEGs shared with the other two sample. Numbers above bars show the percentage of DEGs that are shared with the other two sample.

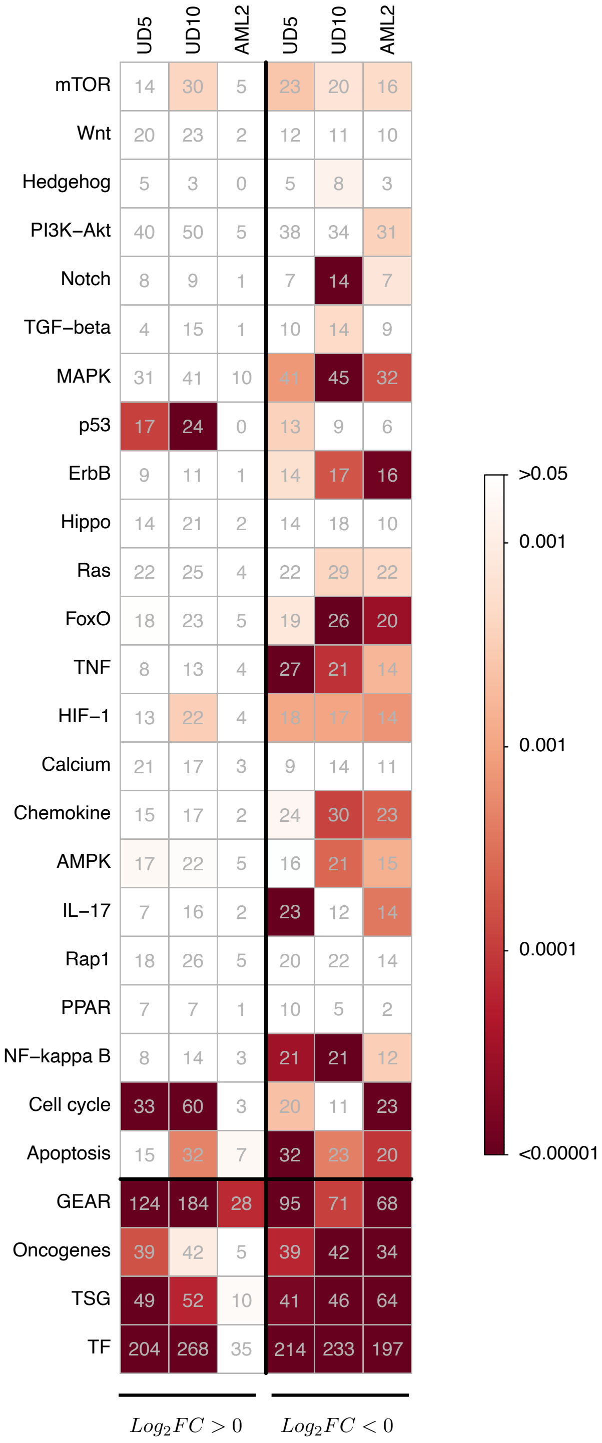

Figure 20: Over-representation analysis of DE genes. Each column of the matrix reports the results of over-representation analysis for the over- and under-expressed genes of each of the three sample. Fisher exact test is calculated for TF, COSMIC, NCG and for cancer-related pathways selected from KEGG and Reactome databases

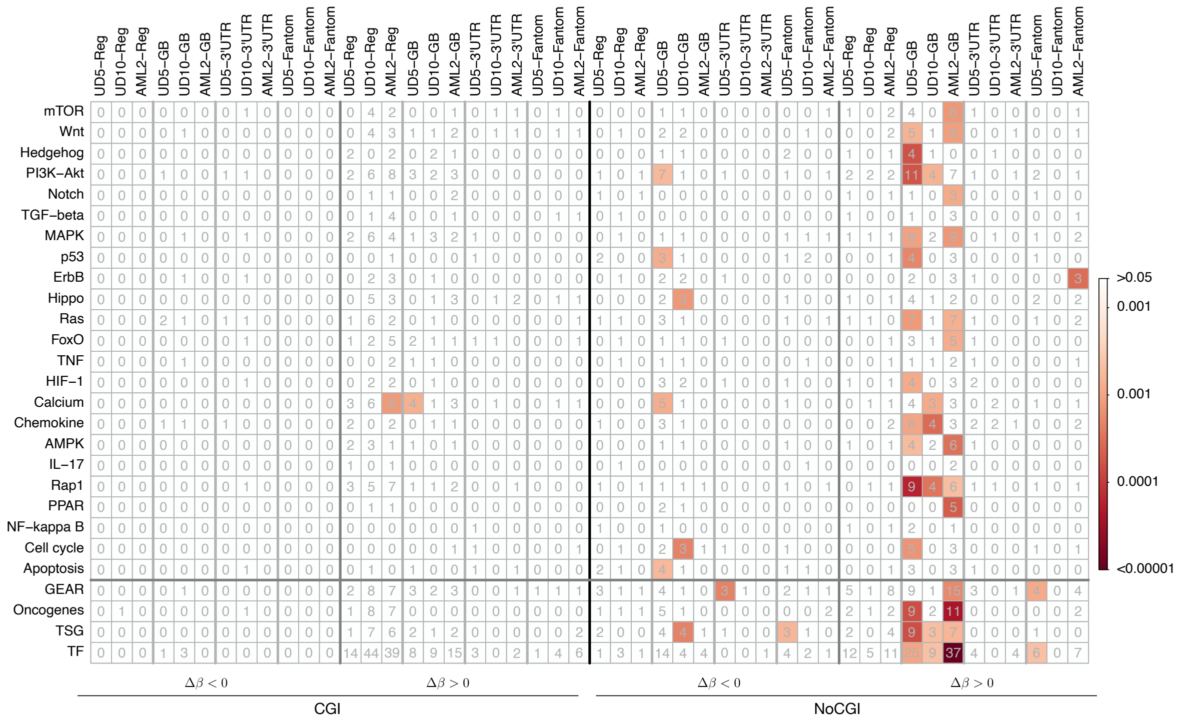

Figure 21: results of ORA of genes with DMR affecting 5' regulatory region (Reg), the gene body (GB), the 3'UTR and the enhancer region predicted by FANTOM. The analysis is performed separately for DMRs overlapping CpG islands (CGI) and outside CpG islands (NoCGI) for cancer-related pathways selected from KEGG database, TF, TSG and Oncogenes selected by COSMIC and GEAR genes. In each cell is reported the total number of DM genes for each category, while color reflect the p-value of the ORA analysis. P-values are obtained by testing (with Fisher exact test) the number of genes that overlap DMRs.

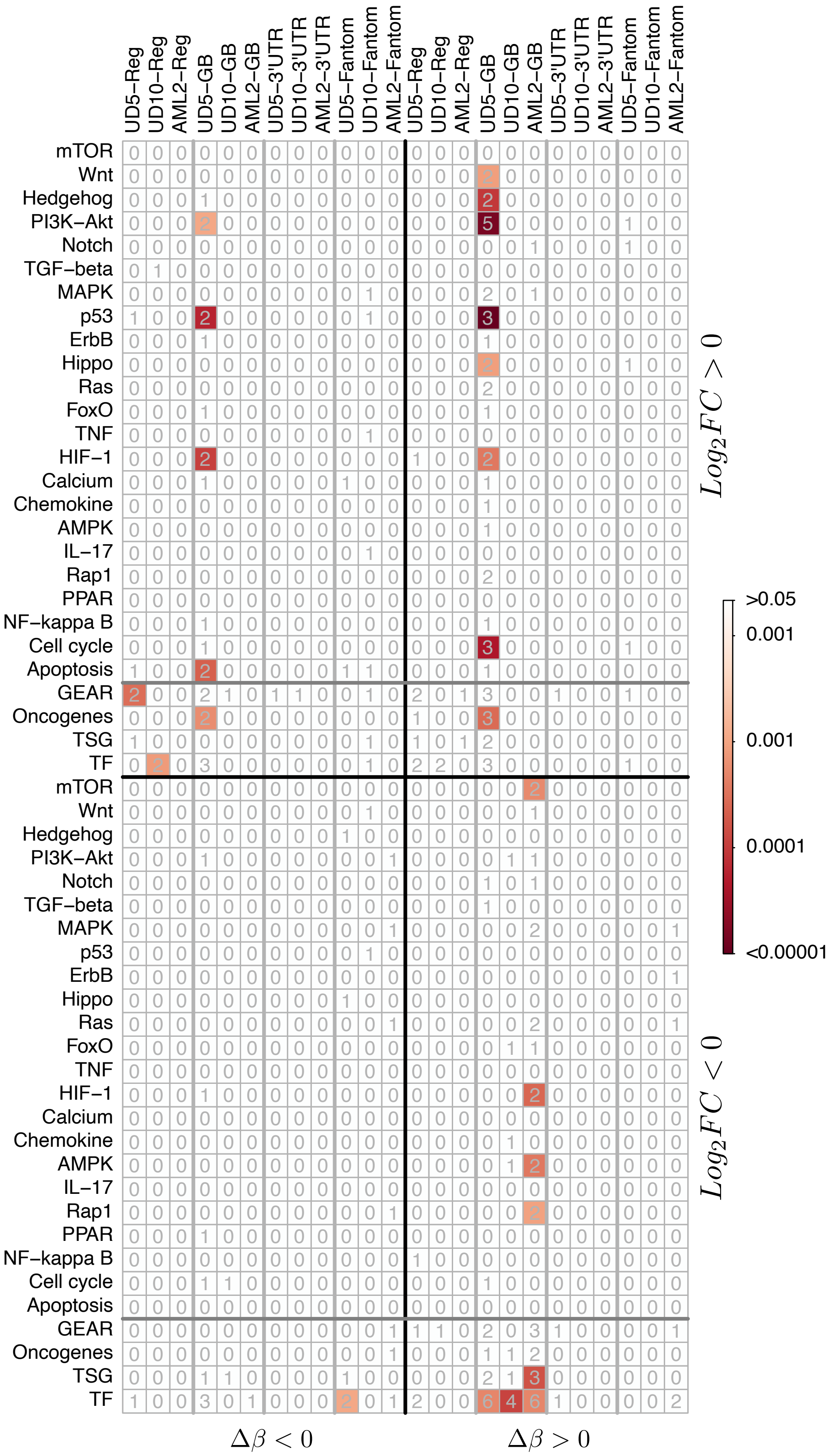

Figure 22: Over-representation analysis of DM-DE genes with DMRs overlapping sparse CpG regions at different genomic elements. Each cell of the matrix reports the results of overrepresentation analysis for all the four epigenetic regulation (hypo-methylation/over-expression, hyper-methylation/under-expression, hyper-methylation/over-expression and hypo-methylation/under-expression). The analysis was performed separately for genes affected by DMRs at the 5' regulatory region (Reg), the gene body (GB), the 3'UTR and at the enhancer region predicted by FANTOM. The numbers in each cell represent the total number of genes for each category, while the color intensity reflects statistical significance according to colorbar. Fisher exact test and number of genes were calculated for TF, TSG, Oncogenes, GEAR and for cancer-related pathways selected from KEGG.

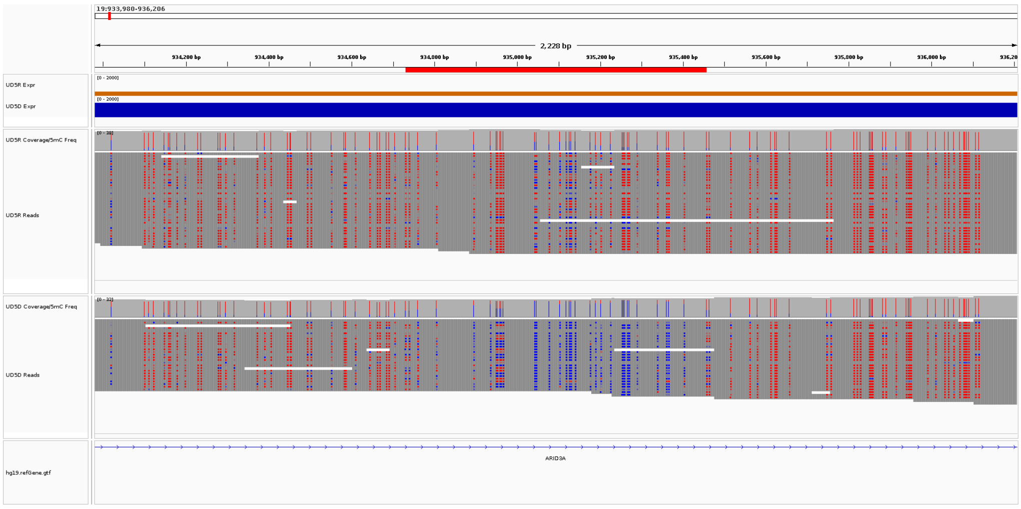

Figure 23: IGV plot of the DMR identified by PoreMeth on the gene-body of ARID3A gene in UD5 sample. The horizontal red bar indicates the start and the end of the DMR detected by PoreMeth. The height of orange (for Relapse) and blue (for Diagnosis) bars show the expression levels according to DESeq2 normalization procedure. Red vertical lines in the the coverage bars show the methylation frequency β for each CpG site. Gray horizontal bars represent aligned reads, red markers represent methylated CpG sites while blue markers represents unmethylated CpG sites.

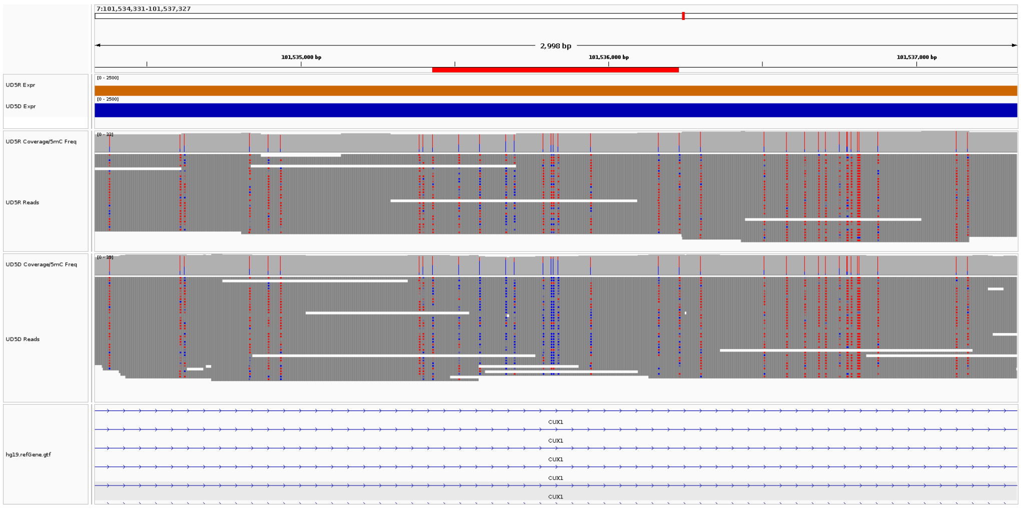

Figure 24: IGV plot of the DMR identified by PoreMeth on the gene-body of CUX1 gene in UD5 sample. The horizontal red bar indicates the start and the end of the DMR detected by PoreMeth. The height of orange (for Relapse) and blue (for Diagnosis) bars show the expression levels according to DESeq2 normalization procedure. Red vertical lines in the the coverage bars show the methylation frequency β for each CpG site. Gray horizontal bars represent aligned reads and red markers represent methylated CpG sites while blue markers represents unmethylated CpG sites.

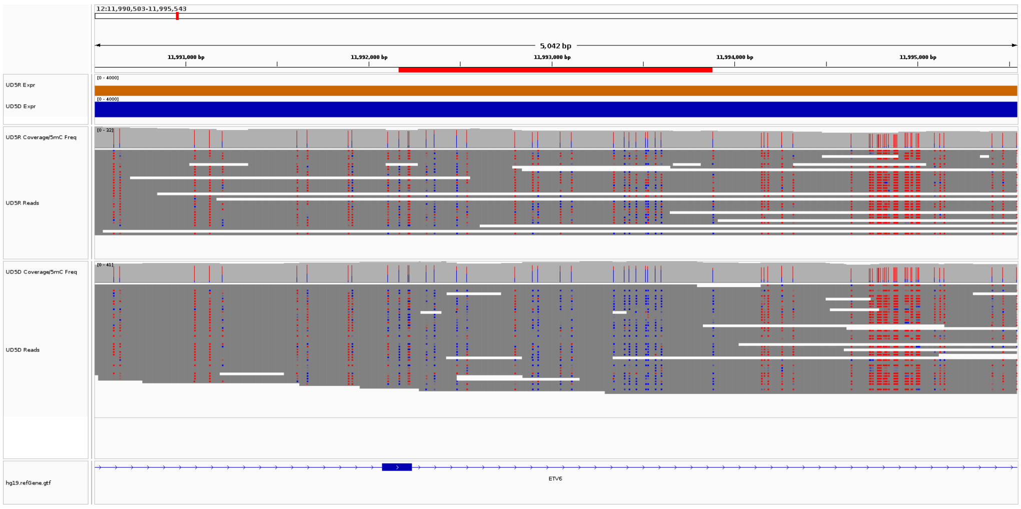

Figure 25: IGV plot of the DMR identified by PoreMeth on the gene-body of ETV6 gene in UD5 sample. The horizontal red bar indicates the start and the end of the DMR detected by PoreMeth. The height of orange (for Relapse) and blue (for Diagnosis) bars show the expression levels according to DESeq2 normalization procedure. Red vertical lines in the the coverage bars show the methylation frequency β for each CpG site. Gray horizontal bars represent aligned reads and red markers represent methylated CpG sites while blue markers represents unmethylated CpG sites.

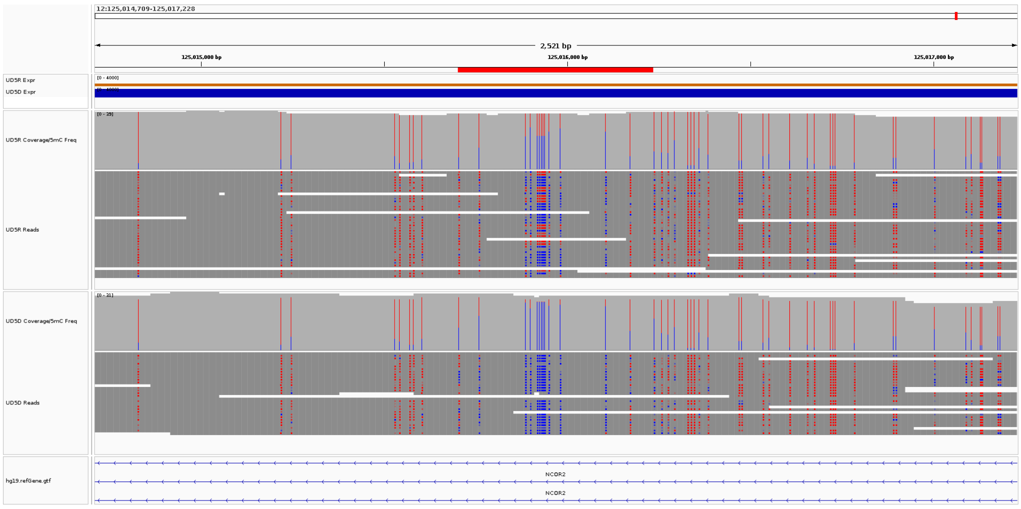

Figure 26: IGV plot of the DMR identified by PoreMeth on the gene-body of NCOR2 gene in UD5 sample. The horizontal red bar indicates the start and the end of the DMR detected by PoreMeth. The height of orange (for Relapse) and blue (for Diagnosis) bars show the expression levels according to DESeq2 normalization procedure. Red vertical lines in the the coverage bars show the methylation frequency β for each CpG site. Gray horizontal bars represent aligned reads and red markers represent methylated CpG sites while blue markers represents unmethylated CpG sites.

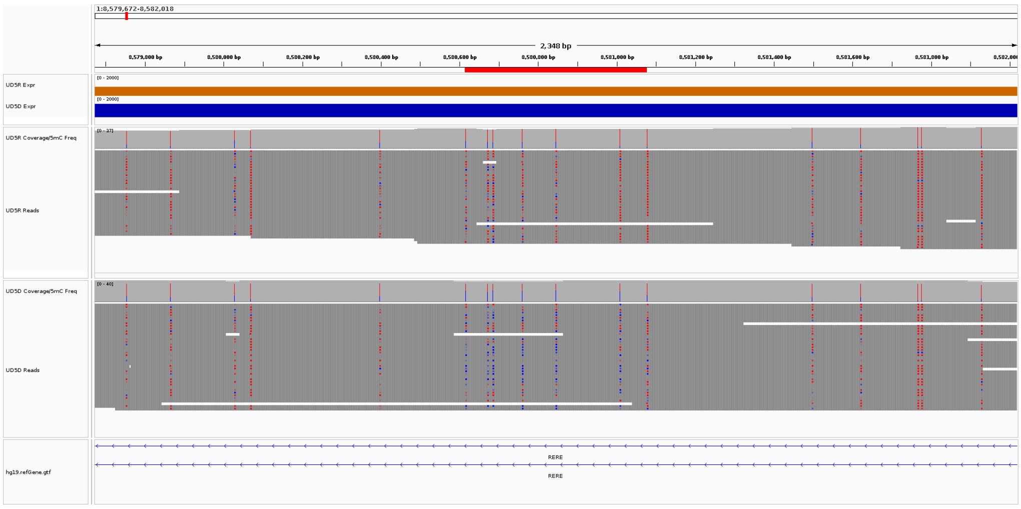

Figure 27: IGV plot of the DMR identified by PoreMeth on the gene-body of RERE gene in UD5 sample. The horizontal red bar indicates the start and the end of the DMR detected by PoreMeth. The height of orange (for Relapse) and blue (for Diagnosis) bars show the expression levels according to DESeq2 normalization procedure. Red vertical lines in the the coverage bars show the methylation frequency β for each CpG site. Gray horizontal bars represent aligned reads and red markers represent methylated CpG sites while blue markers represents unmethylated CpG sites.

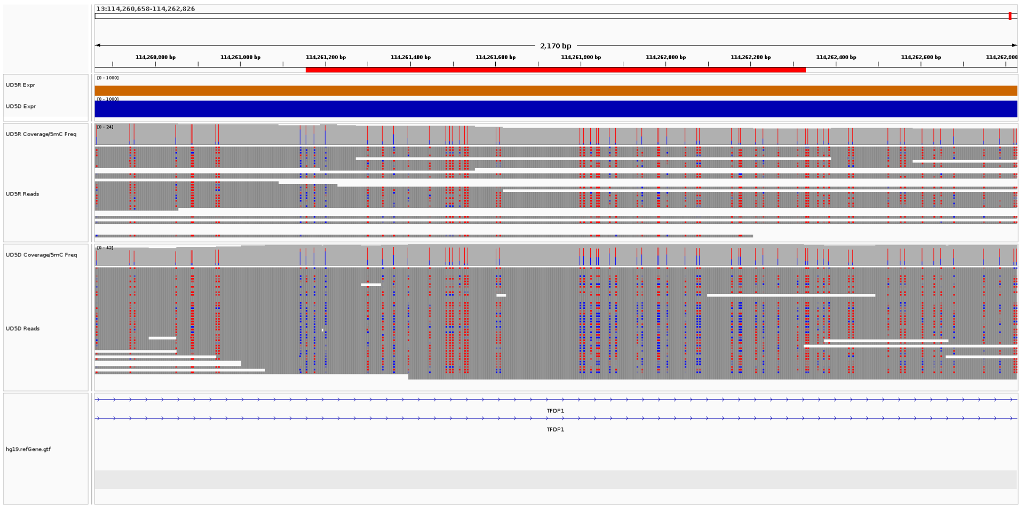

Figure 28: IGV plot of the DMR identified by PoreMeth on the gene-body of TFDP1 gene in UD5 sample. The horizontal red bar indicates the start and the end of the DMR detected by PoreMeth. The height of orange (for Relapse) and blue (for Diagnosis) bars show the expression levels according to DESeq2 normalization procedure. Red vertical lines in the the coverage bars show the methylation frequency β for each CpG site. Gray horizontal bars represent aligned reads and red markers represent methylated CpG sites while blue markers represents unmethylated CpG sites.

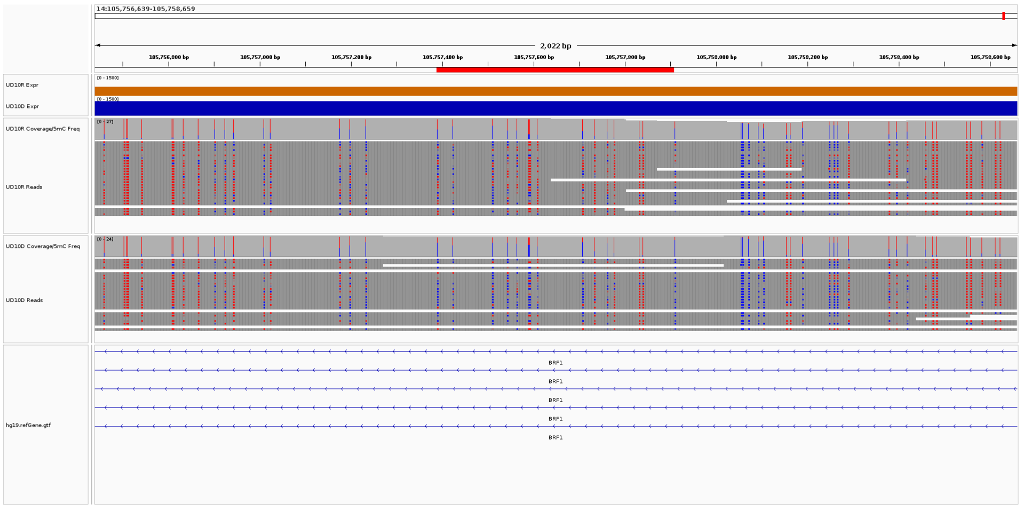

Figure 29: IGV plot of the DMR identified by PoreMeth on the gene-body of BRF1 gene in UD10 sample. The horizontal red bar indicates the start and the end of the DMR detected by PoreMeth. The height of orange (for Relapse) and blue (for Diagnosis) bars show the expression levels according to DESeq2 normalization procedure. Red vertical lines in the the coverage bars show the methylation frequency β for each CpG site. Gray horizontal bars represent aligned reads and red markers represent methylated CpG sites while blue markers represents unmethylated CpG sites.

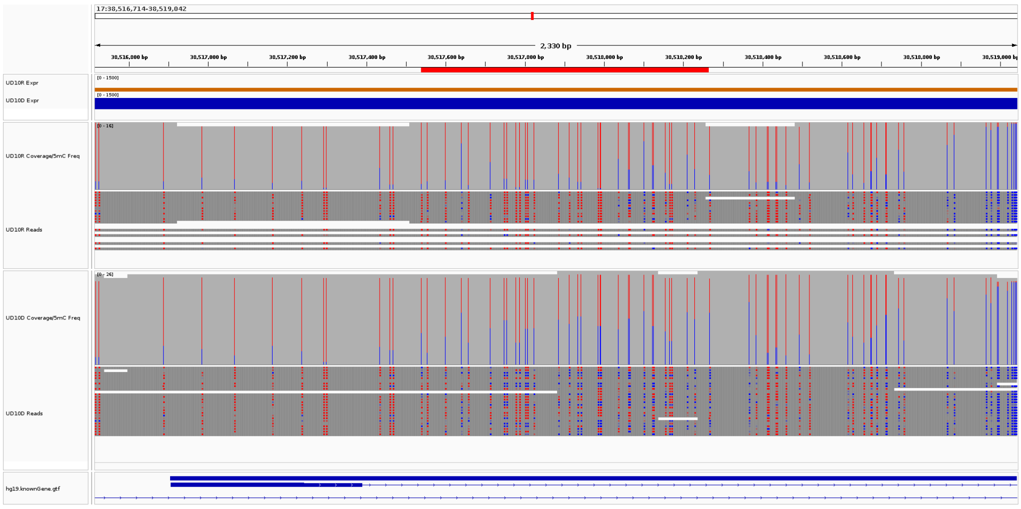

Figure 30: IGV plot of the DMR identified by PoreMeth on the gene-body of RARA gene in UD10 sample. The horizontal red bar indicates the start and the end of the DMR detected by PoreMeth. The height of orange (for Relapse) and blue (for Diagnosis) bars show the expression levels according to DESeq2 normalization procedure. Red vertical lines in the the coverage bars show the methylation frequency β for each CpG site. Gray horizontal bars represent aligned reads and red markers represent methylated CpG sites while blue markers represents unmethylated CpG sites.

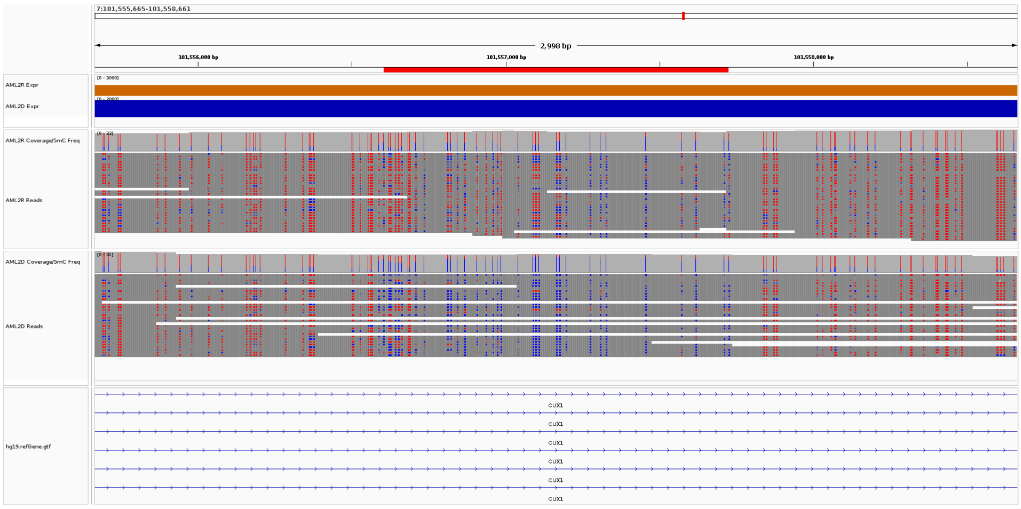

Figure 31: IGV plot of the DMR identified by PoreMeth on the gene-body of CUX1 gene in UD5 sample. The horizontal red bar indicates the start and the end of the DMR detected by PoreMeth. The height of orange (for Relapse) and blue (for Diagnosis) bars show the expression levels according to DESeq2 normalization procedure. Red vertical lines in the the coverage bars show the methylation frequency β for each CpG site. Gray horizontal bars represent aligned reads and red markers represent methylated CpG sites while blue markers represents unmethylated CpG sites.

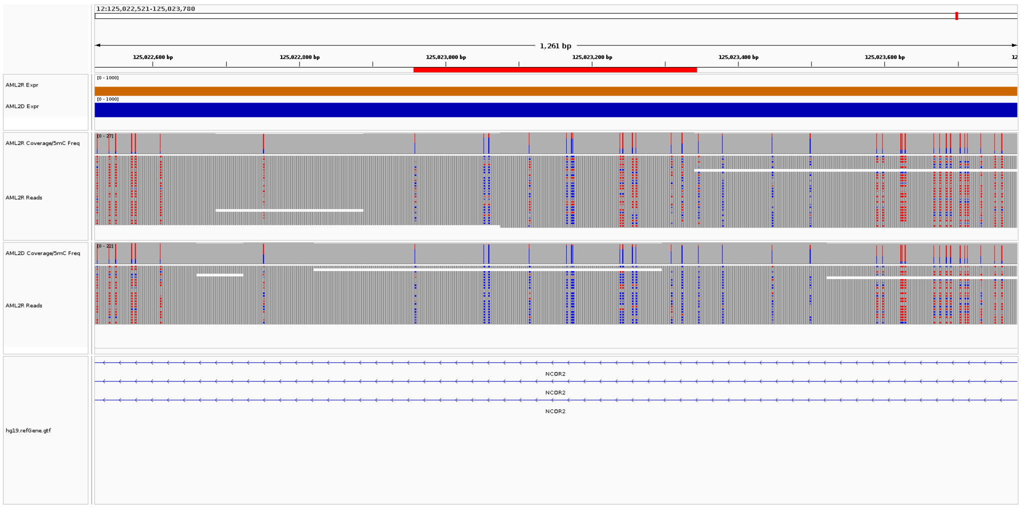

Figure 32: IGV plot of the DMR identified by PoreMeth on the gene-body of NCOR2 gene in AML2 sample. The horizontal red bar indicates the start and the end of the DMR detected by PoreMeth. The height of orange (for Relapse) and blue (for Diagnosis) bars show the expression levels according to DESeq2 normalization procedure. Red vertical lines in the the coverage bars show the methylation frequency β for each CpG site. Gray horizontal bars represent aligned reads and red markers represent methylated CpG sites while blue markers represents unmethylated CpG sites.

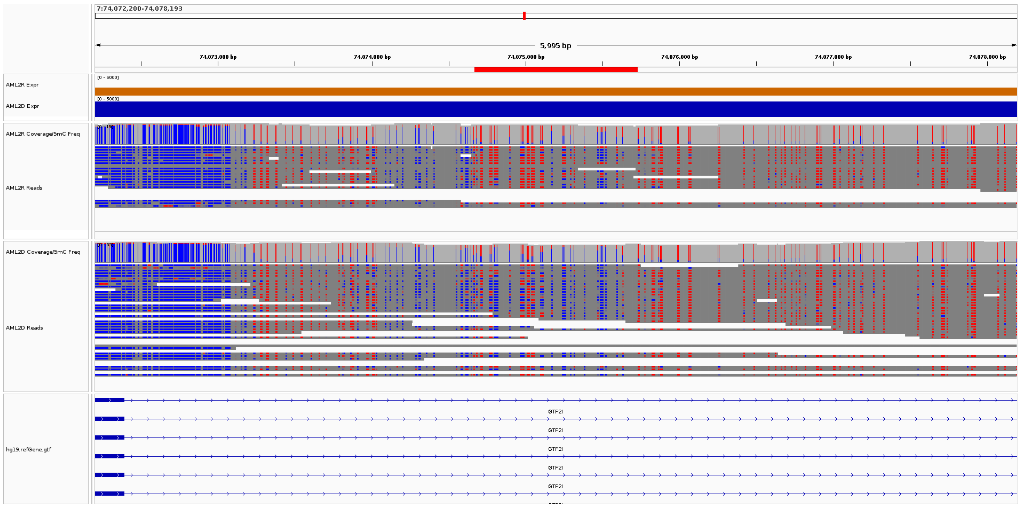

Figure 33: IGV plot of the DMR identified by PoreMeth on the gene-body of GTF2I gene in AML2 sample. The horizontal red bar indicates the start and the end of the DMR detected by PoreMeth. The height of orange (for Relapse) and blue (for Diagnosis) bars show the expression levels according to DESeq2 normalization procedure. Red vertical lines in the the coverage bars show the methylation frequency β for each CpG site. Gray horizontal bars represent aligned reads and red markers represent methylated CpG sites while blue markers represents unmethylated CpG sites.

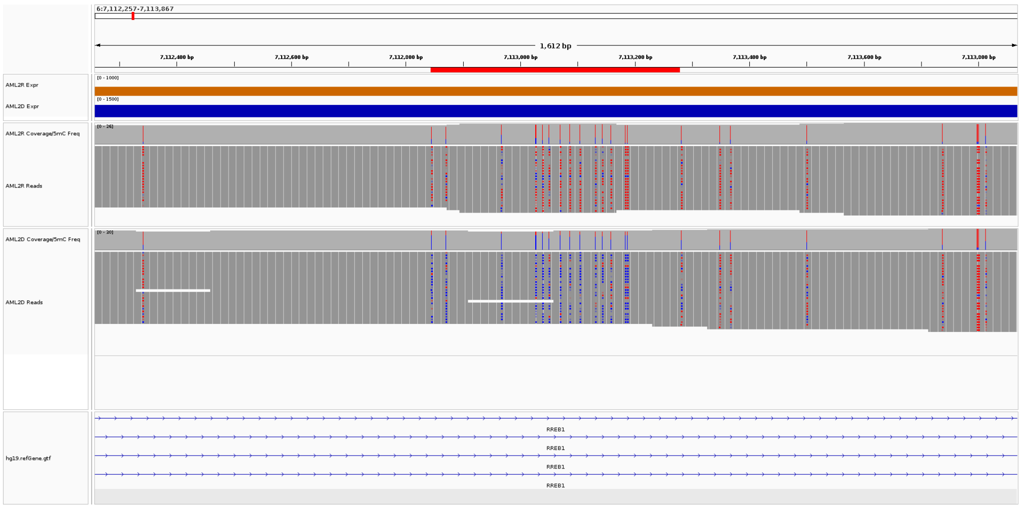

Figure 34: IGV plot of the DMR identified by PoreMeth on the gene-body of RREB1 gene in AML2 sample. The horizontal red bar indicates the start and the end of the DMR detected by PoreMeth. The height of orange (for Relapse) and blue (for Diagnosis) bars show the expression levels according to DESeq2 normalization procedure. Red vertical lines in the the coverage bars show the methylation frequency β for each CpG site. Gray horizontal bars represent aligned reads and red markers represent methylated CpG sites while blue markers represents unmethylated CpG sites.

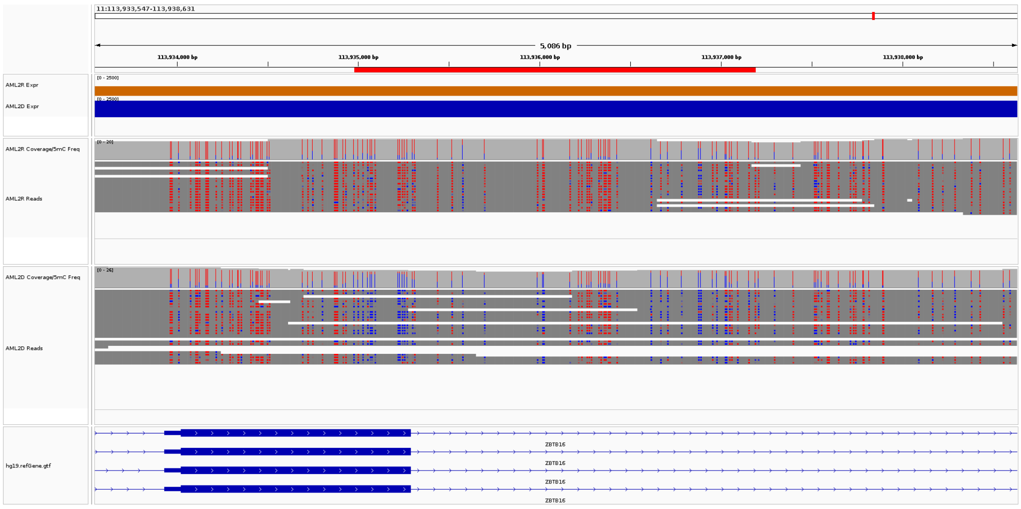

Figure 35: IGV plot of the DMR identified by PoreMeth on the gene-body of ZBTB16 gene in AML2 sample. The horizontal red bar indicates the start and the end of the DMR detected by PoreMeth. The height of orange (for Relapse) and blue (for Diagnosis) bars show the expression levels according to DESeq2 normalization procedure. Red vertical lines in the the coverage bars show the methylation frequency β for each CpG site. Gray horizontal bars represent aligned reads and red markers represent methylated CpG sites while blue markers represents unmethylated CpG sites.

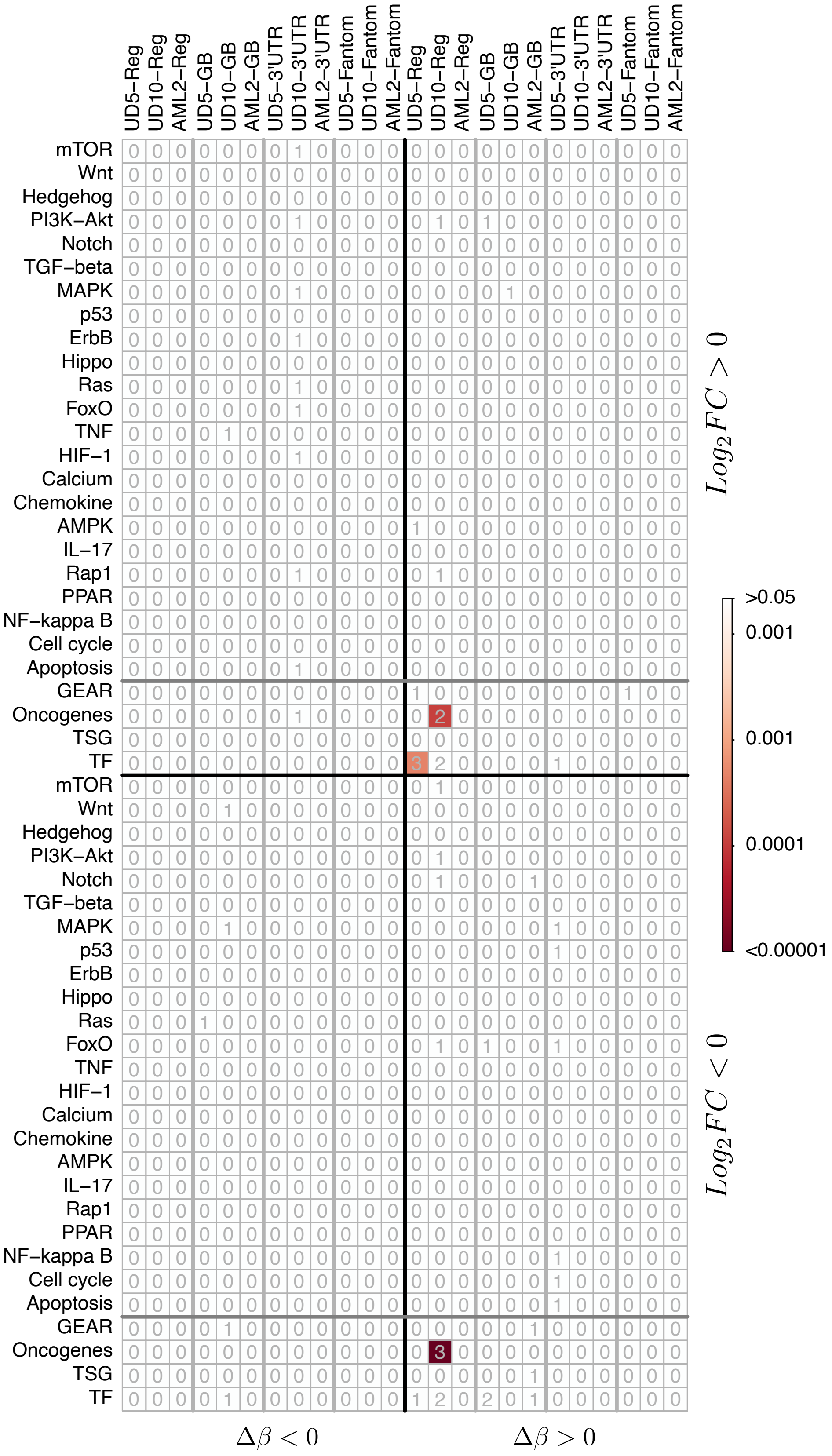

Figure 36: Over-representation analysis of DM-DE genes with DMRs overlapping CGIs at different genomic elements. Each cell of the matrix reports the results of overrepresentation analysis for all the four epigenetic regulation (hypo-methylation/over-expression, hyper-methylation/under-expression, hyper-methylation/over-expression and hypo-methylation/under-expression). The analysis was performed separately for genes affected by DMRs at the 5' regulatory region (Reg), the gene body (GB), the 3'UTR and at the enhancer region predicted by FANTOM. The numbers in each cell represent the total number of genes for each category, while the color intensity reflects statistical significance according to colorbar. Fisher exact test and number of genes were calculated for TF, TSG, Oncogenes, GEAR and for cancer-related pathways selected from KEGG.

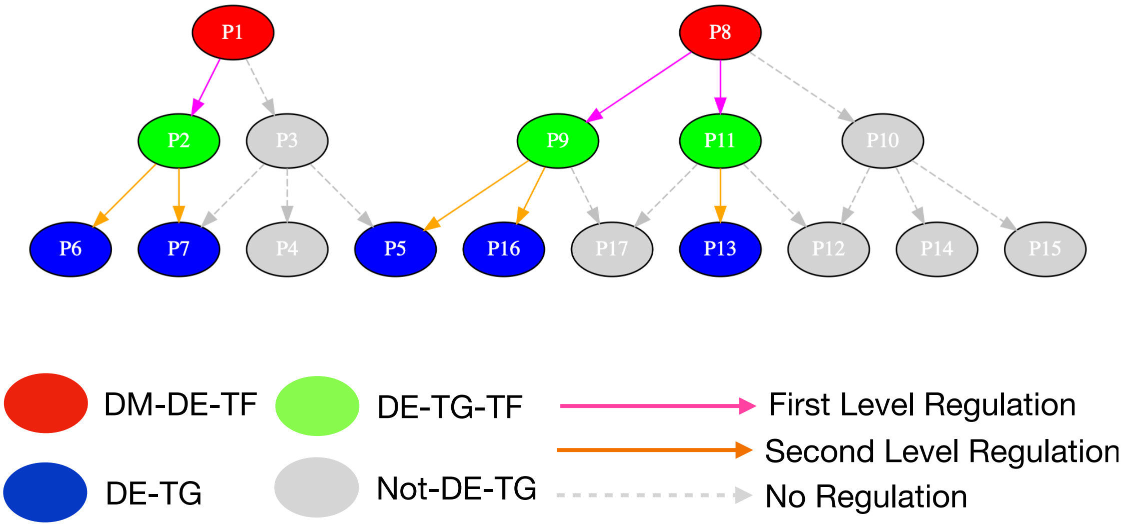

Figure 37: Figure shows the scheme we used to study the effect of DMRs on gene regulatory network cascade. Red nodes represent differentially methylated and differentially expressed TFs (DM-DE-TF). Green nodes are differentially expressed TFs regulated by DM-DE-TF (DE-TG-TF). Blue nodes are differentially expressed genes regulated by DM-DE-TF or by DE-TG-TF. Grey nodes are target genes that are not differentially expressed (Not-DE-TG). Magenta arrows indicate a first level regulation (DM-DE-TF regulate a DE target gene). Orange arrow indicate a second level regulation (DE-TG-TF regulate a DE target gene). Grey arrows indicate no regulation.

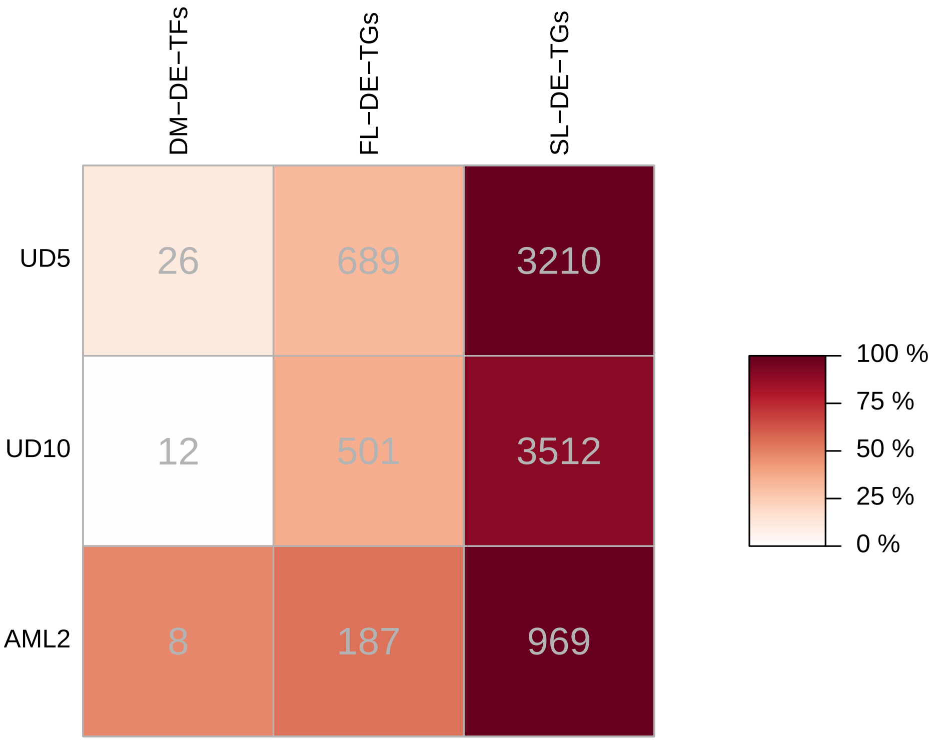

Figure 38: The table summarizes the total number of DEG in each level of the regulatory cascade induced by DMRs. In each cell of the first column is reported the total number of differentially methylated and differentially expressed TFs (DM-DE-TS). In the second column the total number of first-level target genes (FL-DE-TGs) and in the third column the total number of second-level target genes (SL-DE-TGs). For each AML sample the color intensity in each cell shows the proportion of genes shared with the other two samples.

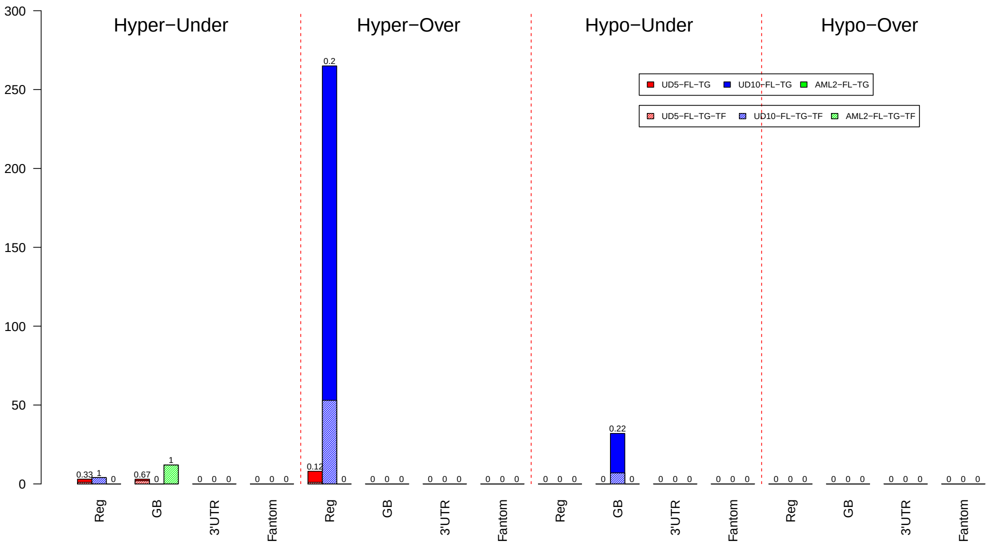

Figure 39: The barplot reports the total number of first-level target genes (FL-DE-TGs) potentially regulated by DM-DE-TFs with DMRs in CGIs. Each bar reports the number of FL-DE-TGs regulated by under- or over-expressed DM-DE-TFs with hyper- or hypo-methylated DMRs at 5' regulatory-elements, gene bodies, 3'UTRs and enhancers. For each category, textured bars show the number of FL-DE-TGs shared with the other two samples. Numbers above bars show the percentage of DEGs that are shared with the other two sample.

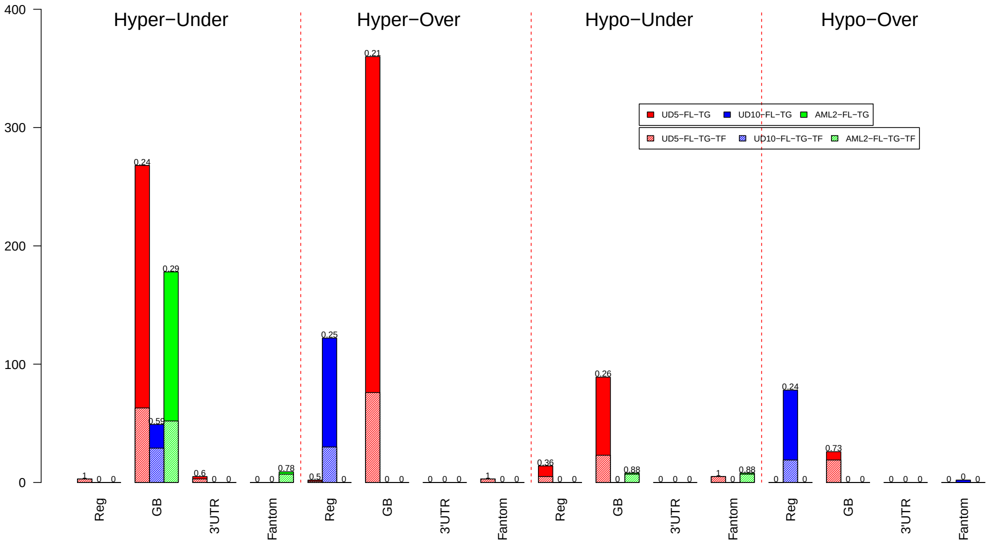

Figure 40: The barplot reports the total number of first-level target genes (FL-DE-TGs) potentially regulated by DM-DE-TFs with DMRs in sparse CpG regions. Each bar reports the number of FL-DE-TGs regulated by under- or over-expressed DM-DE-TFs with hyper- or hypo-methylated DMRs at 5' regulatory-elements, gene bodies, 3'UTRs and enhancers. For each category, textured bars show the number of FL-DE-TGs shared with the other two samples. Numbers above bars show the percentage of DEGs that are shared with the other two sample.

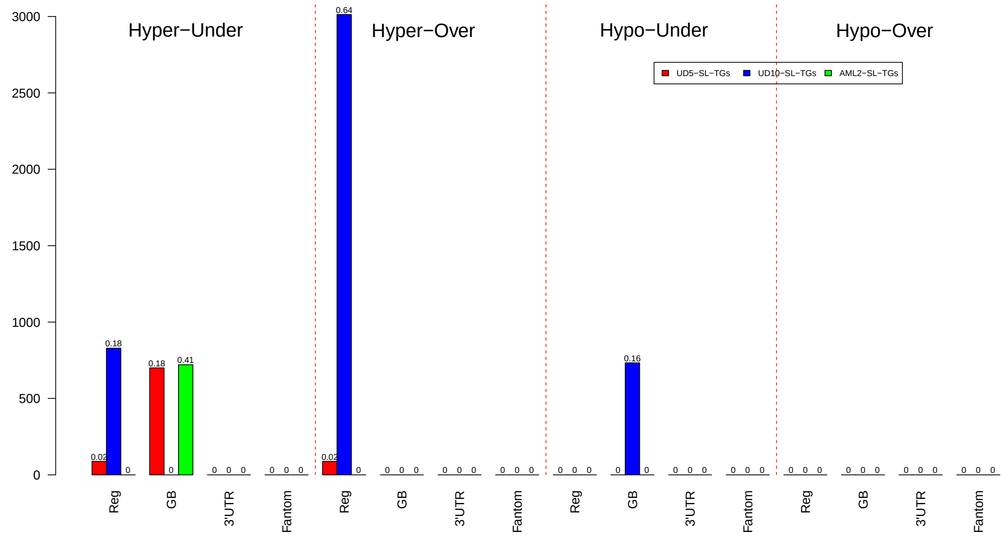

Figure 41: The barplot reports the total number of first- and second-level target genes (FL-DE-TGs) potentially regulated by DM-DE-TFs with DMRs in CGIs. Each bar reports the number of FL-and SL-DE-TGs regulated by under- or over-expressed DM-DE-TFs with hyper- or hypo-methylated DMRs at 5' regulatory-elements, gene bodies, 3'UTRs and enhancers. For each category, numbers above bars show the fraction of FL-and SL-DE-TGs with respect to all under- or over-expressed genes for each sample.

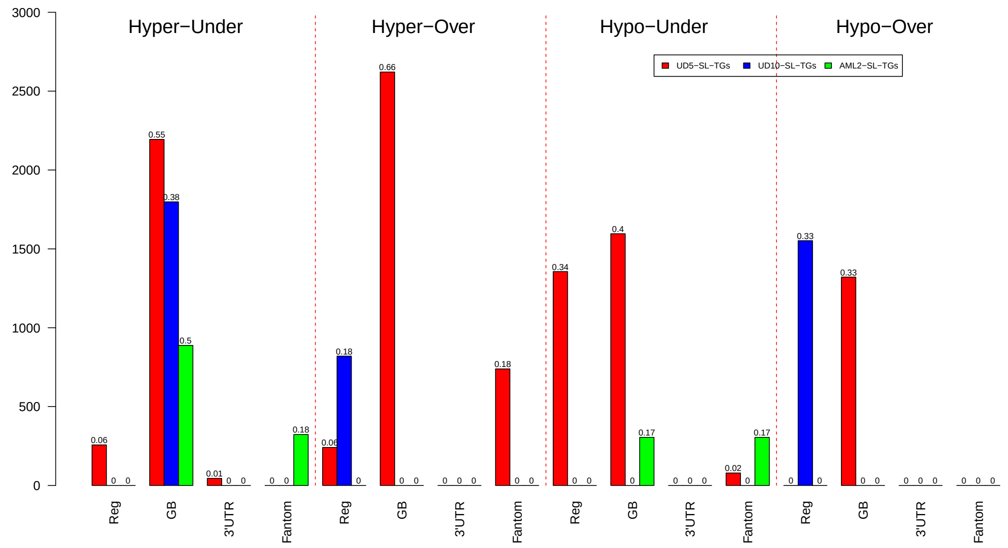

Figure 42: The barplot reports the total number of first- and second-level target genes (FL-DE-TGs) potentially regulated by DM-DE-TFs with DMRs in sparse CpG regions. Each bar reports the number of FL- and SL-DE-TGs regulated by under- or over-expressed DM-DE-TFs with hyper- or hypo-methylated DMRs at 5' regulatory-elements, gene bodies, 3'UTRs and enhancers. For each category, numbers above bars show the fraction of FL-and SL-DE-TGs with respect to all under- or over-expressed genes for each sample.

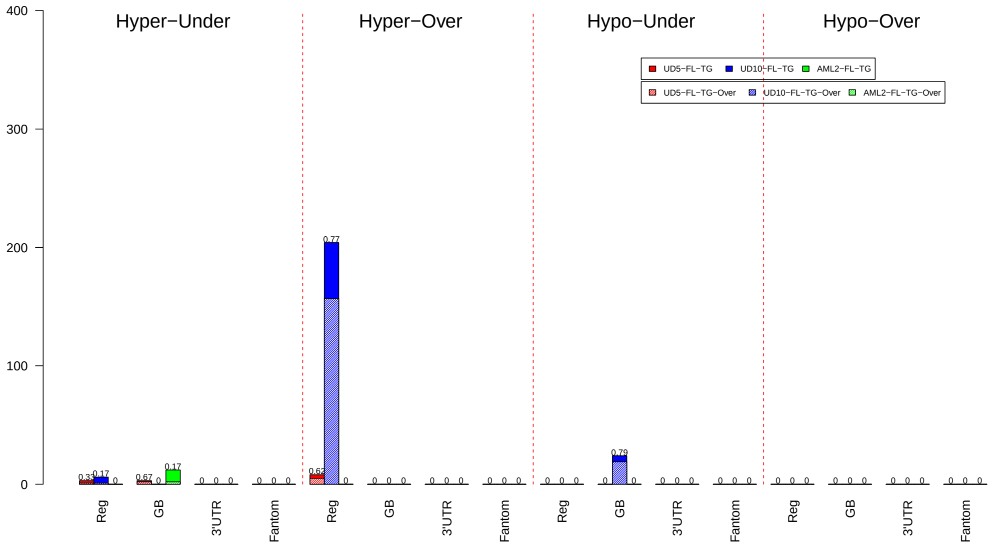

Figure 43: The barplot reports the total number of over- and under-expressed first-level target genes (FL-DE-TGs) potentially regulated by DM-DE-TFs with DMRs in CGIs. Each bar reports the number of FL-DE-TGs regulated by under- or over-expressed DM-DE-TFs with hyper- or hypo-methylated DMRs at 5' regulatory-elements, gene bodies, 3'UTRs and enhancers. For each category, textured bars show the number of over-expressed FL-DE-TGs while solid bars the number of under-expressed FL-DE-TGs. Numbers above bars show the percentage of over-expressed FL-DE-TGs.

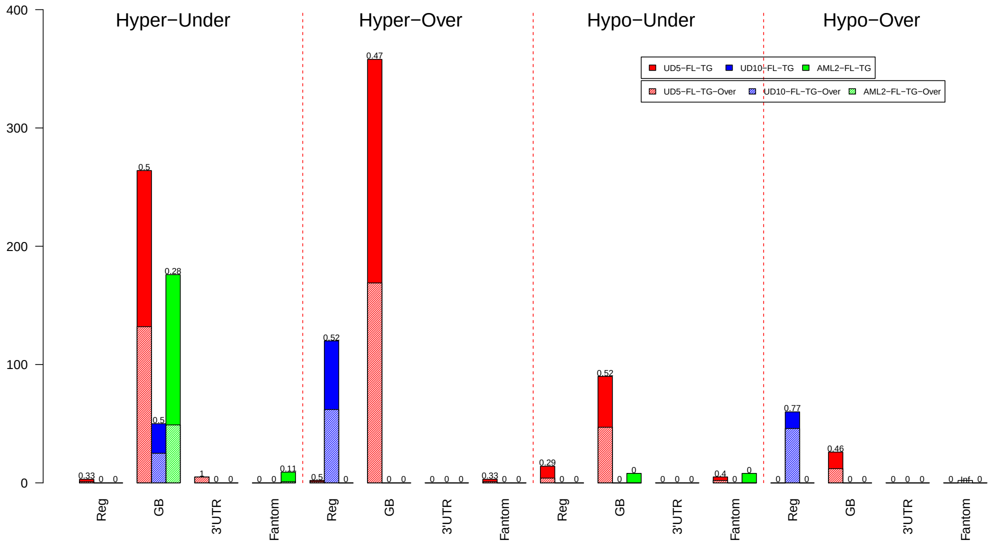

Figure 44: The barplot reports the total number of over- and under-expressed second-level target genes (FL-DE-TGs) potentially regulated by DM-DE-TFs with DMRs in sparse CpG regions. Each bar reports the number of FL-DE-TGs regulated by under- or over-expressed DM-DE-TFs with hyper- or hypo-methylated DMRs at 5' regulatory-elements, gene bodies, 3'UTRs and enhancers. For each category, textured bars show the number of over-expressed FL-DE-TGs while solid bars the number of under-expressed FL-DE-TGs. Numbers above bars show the percentage of over-expressed FL-DE-TGs.

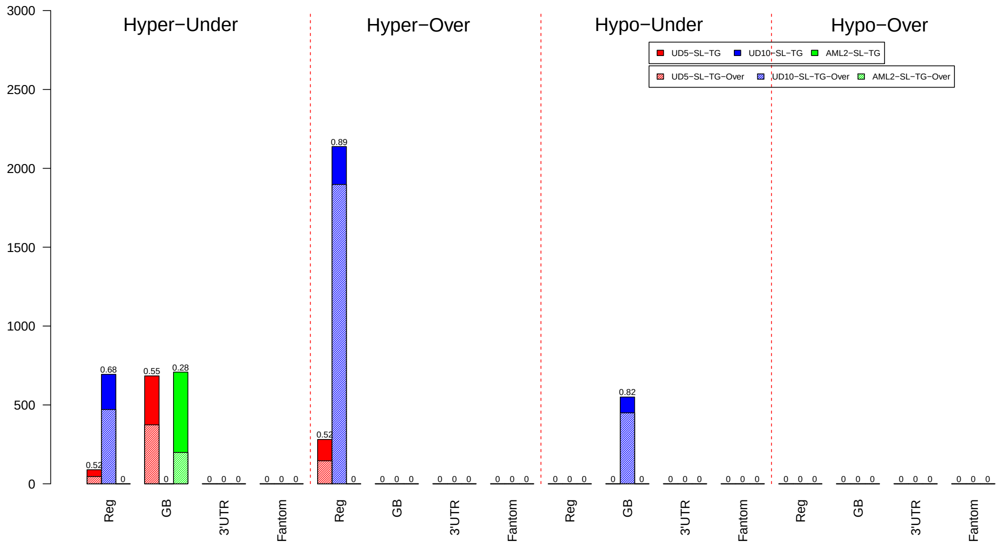

Figure 45: The barplot reports the total number of over- and under-expressed second-level target genes (SL-DE-TGs) potentially regulated by DM-DE-TFs with DMRs in CGIs. Each bar reports the number of SL-DE-TGs regulated by under- or over-expressed DM-DE-TFs with hyper- or hypo-methylated DMRs at 5' regulatory-elements, gene bodies, 3'UTRs and enhancers. For each category, textured bars show the number of over-expressed SL-DE-TGs while solid bars the number of under-expressed SL-DE-TGs. Numbers above bars show the percentage of over-expressed SL-DE-TGs.

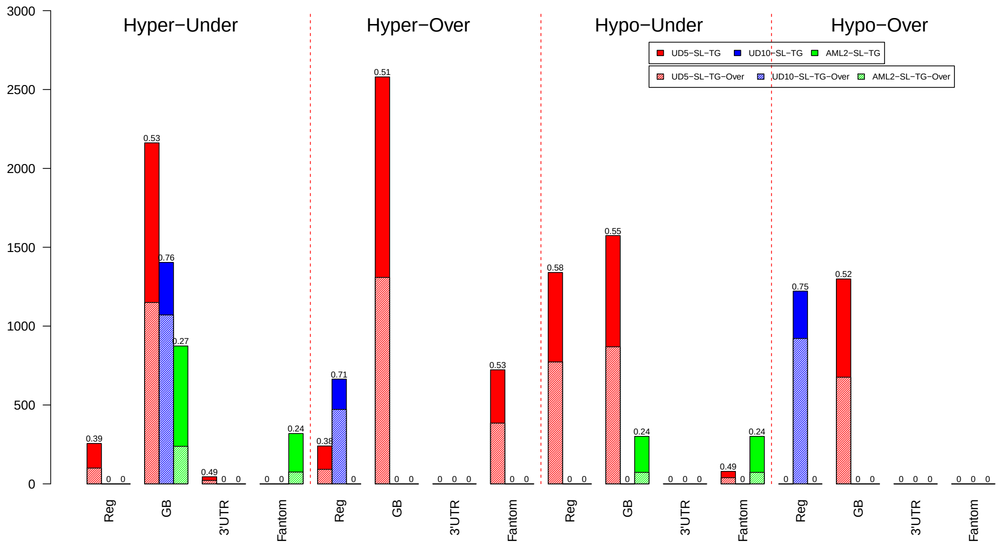

Figure 46: The barplot reports the total number of over- and under-expressed second-level target genes (SL-DE-TGs) potentially regulated by DM-DE-TFs with DMRs in sparse CpG regions. Each bar reports the number of SL-DE-TGs regulated by under- or over-expressed DM-DE-TFs with hyper- or hypo-methylated DMRs at 5' regulatory-elements, gene bodies, 3'UTRs and enhancers. For each category, textured bars show the number of over-expressed SL-DE-TGs while solid bars the number of under-expressed SL-DE-TGs. Numbers above bars show the percentage of over-expressed SL-DE-TGs.

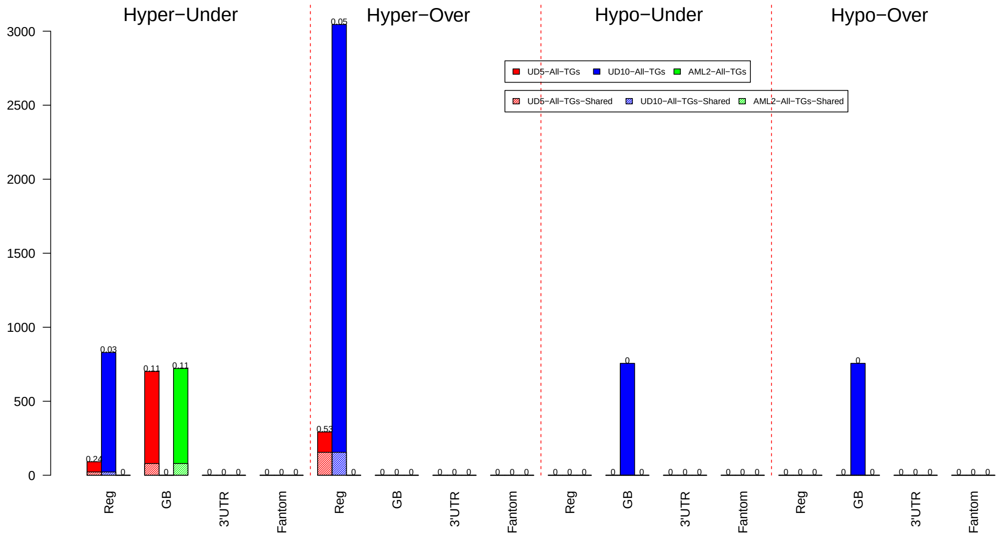

Figure 47: The barplot reports the total number of first- and second-level target genes (FL-DE-TGs) potentially regulated by DM-DE-TFs with DMRs in CGIs. Each bar reports the number of FL- and SL-DE-TGs regulated by under- or over-expressed DM-DE-TFs with hyper- or hypo-methylated DMRs at 5' regulatory-elements, gene bodies, 3'UTRs and enhancers. For each category, textured bars show the number of FL- and SL-DE-TGs shared with the other two samples. Numbers above bars show the percentage of DEGs that are shared with the other two sample.

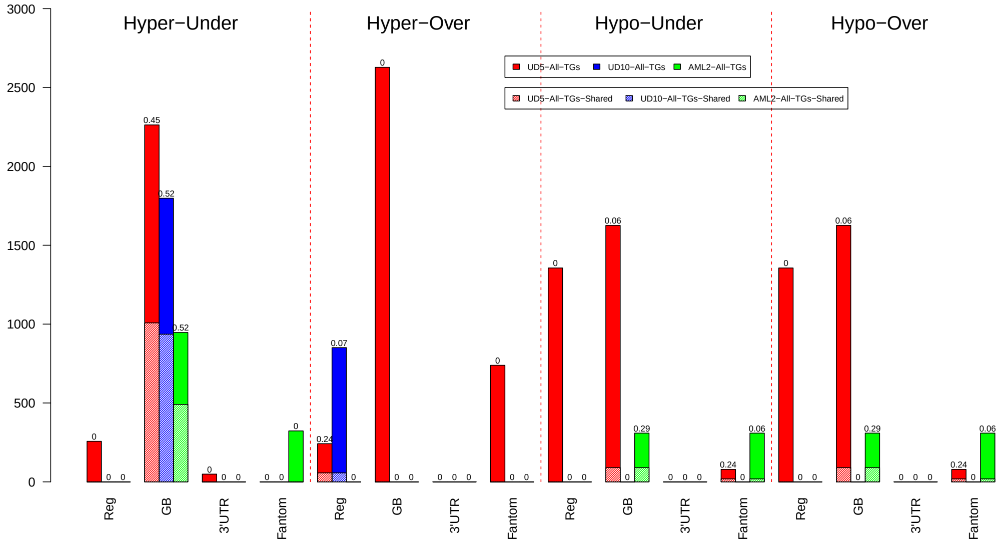

Figure 48: The barplot reports the total number of first- and second-level target genes (FL-DE-TGs) potentially regulated by DM-DE-TFs with DMRs in sparse CpG regions. Each bar reports the number of FL- and SL-DE-TGs regulated by under- or over-expressed DM-DE-TFs with hyper- or hypo-methylated DMRs at 5' regulatory-elements, gene bodies, 3'UTRs and enhancers. For each category, textured bars show the number of FL- and SL-DE-TGs shared with the other two samples. Numbers above bars show the percentage of DEGs that are shared with the other two sample.

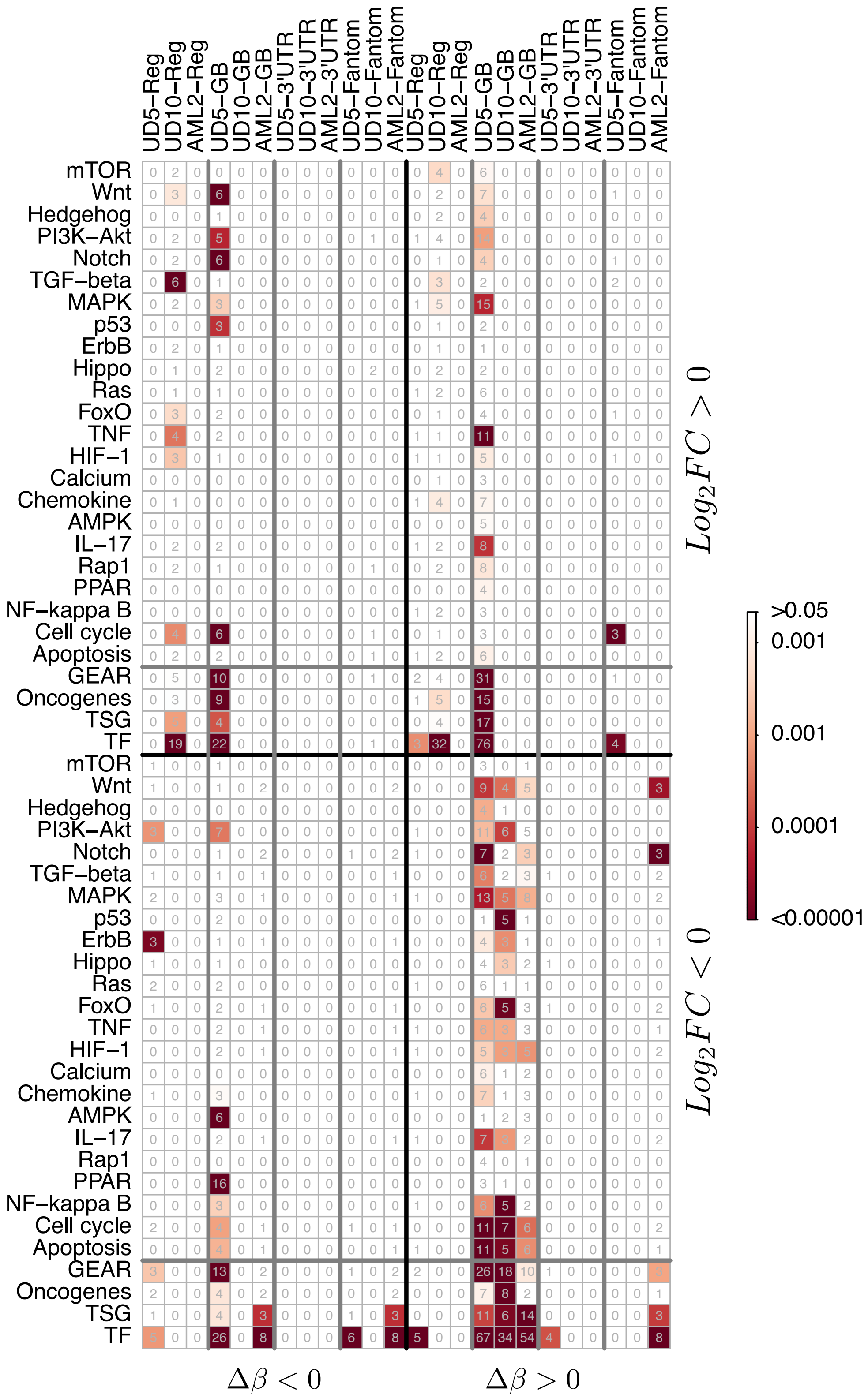

Figure 49: Figure reports the results of ORA on the first-level regulatory network cascade induced by DMRs at sparse CpG regions. Each cell of the matrix reports the results of overrepresentation analysis for all the four epigenetic regulation (hypo-methylation/over-expression, hyper-methylation/under-expression, hyper-methylation/over-expression and hypo-methylation/under-expression). The analysis was performed separately for genes affected by DMRs at the 5' regulatory region (Reg), the gene body (GB), the 3'UTR and at the enhancer region predicted by FANTOM. The numbers in each cell represent the total number of genes for each category, while the color intensity reflects statistical significance according to colorbar. Fisher exact test and number of genes were calculated for cancer-related pathways selected from KEGG database, TFs, TSG and Oncogenes selected by COSMIC and GEAR genes.

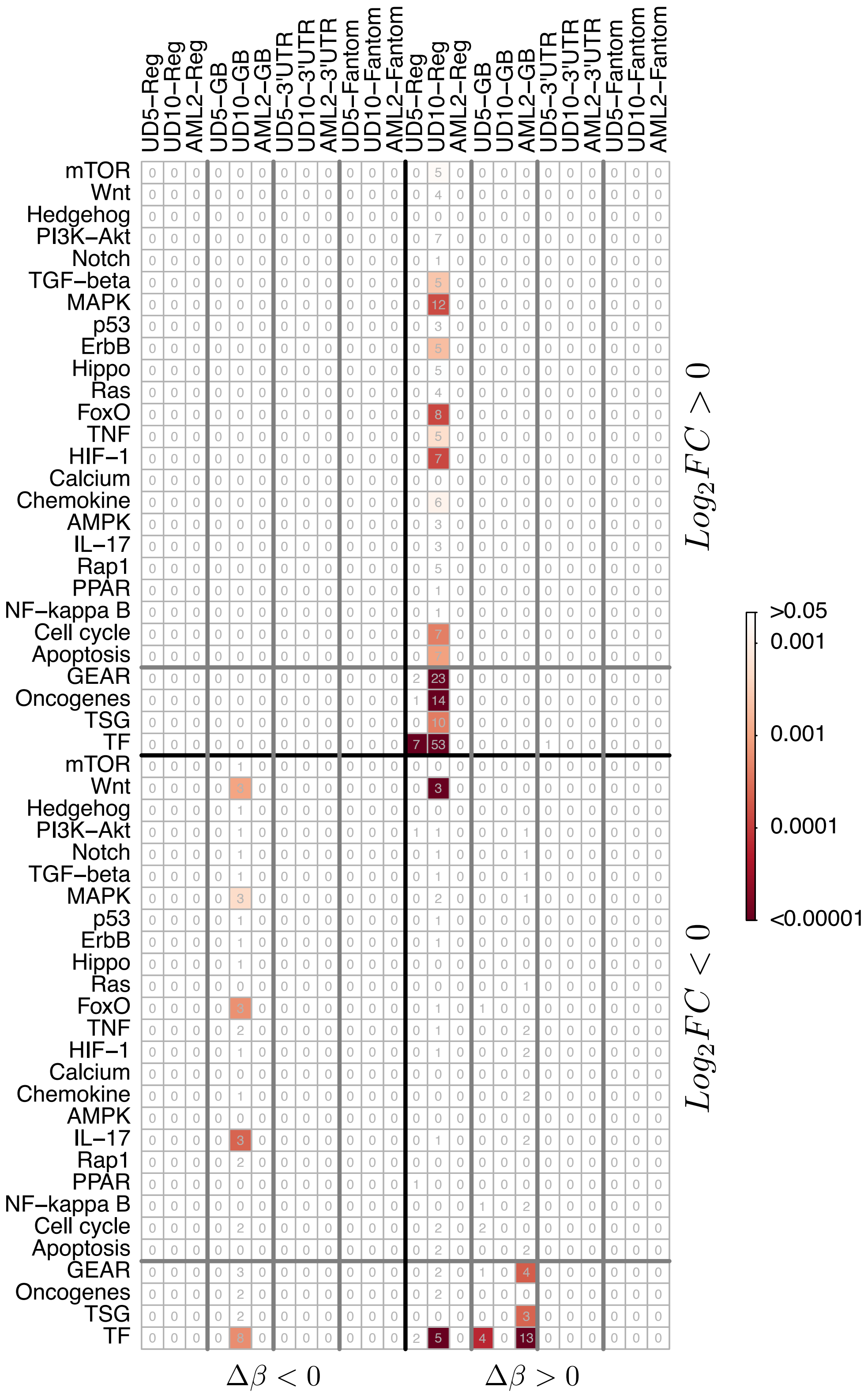

Figure 50: Figure reports the results of ORA on the first-level regulatory network cascade induced by DMRs at CGIs. Each cell of the matrix reports the results of overrepresentation analysis for all the four epigenetic regulation (hypo-methylation/over-expression, hyper-methylation/under-expression, hyper-methylation/over-expression and hypo-methylation/under-expression). The analysis was performed separately for genes affected by DMRs at the 5' regulatory region (Reg), the gene body (GB), the 3'UTR and at the enhancer region predicted by FANTOM. The numbers in each cell represent the total number of genes for each category, while the color intensity reflects statistical significance according to colorbar. Fisher exact test and number of genes were calculated for cancer-related pathways selected from KEGG database, TFs, TSG and Oncogenes selected by COSMIC and GEAR genes.

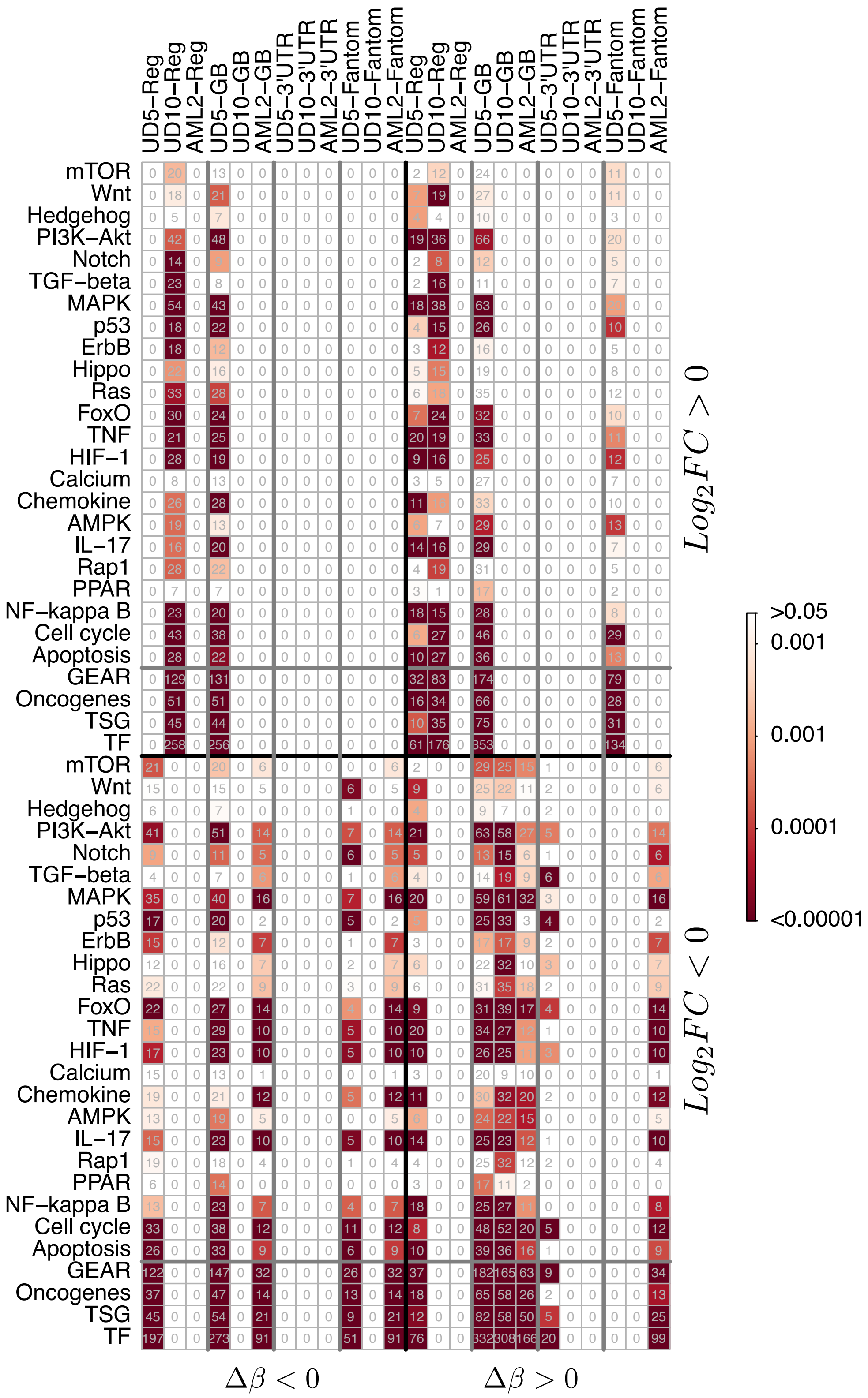

Figure 51: Figure reports the results of ORA on the second-level regulatory network cascade induced by DMRs at sparse CpG regions. Each cell of the matrix reports the results of overrepresentation analysis for all the four epigenetic regulation (hypo-methylation/over-expression, hyper-methylation/under-expression, hyper-methylation/over-expression and hypo-methylation/under-expression). The analysis was performed separately for genes affected by DMRs at the 5' regulatory region (Reg), the gene body (GB), the 3'UTR and at the enhancer region predicted by FANTOM. The numbers in each cell represent the total number of genes for each category, while the color intensity reflects statistical significance according to colorbar. Fisher exact test and number of genes were calculated for cancer-related pathways selected from KEGG database, TFs, TSG and Oncogenes selected by COSMIC and GEAR genes.

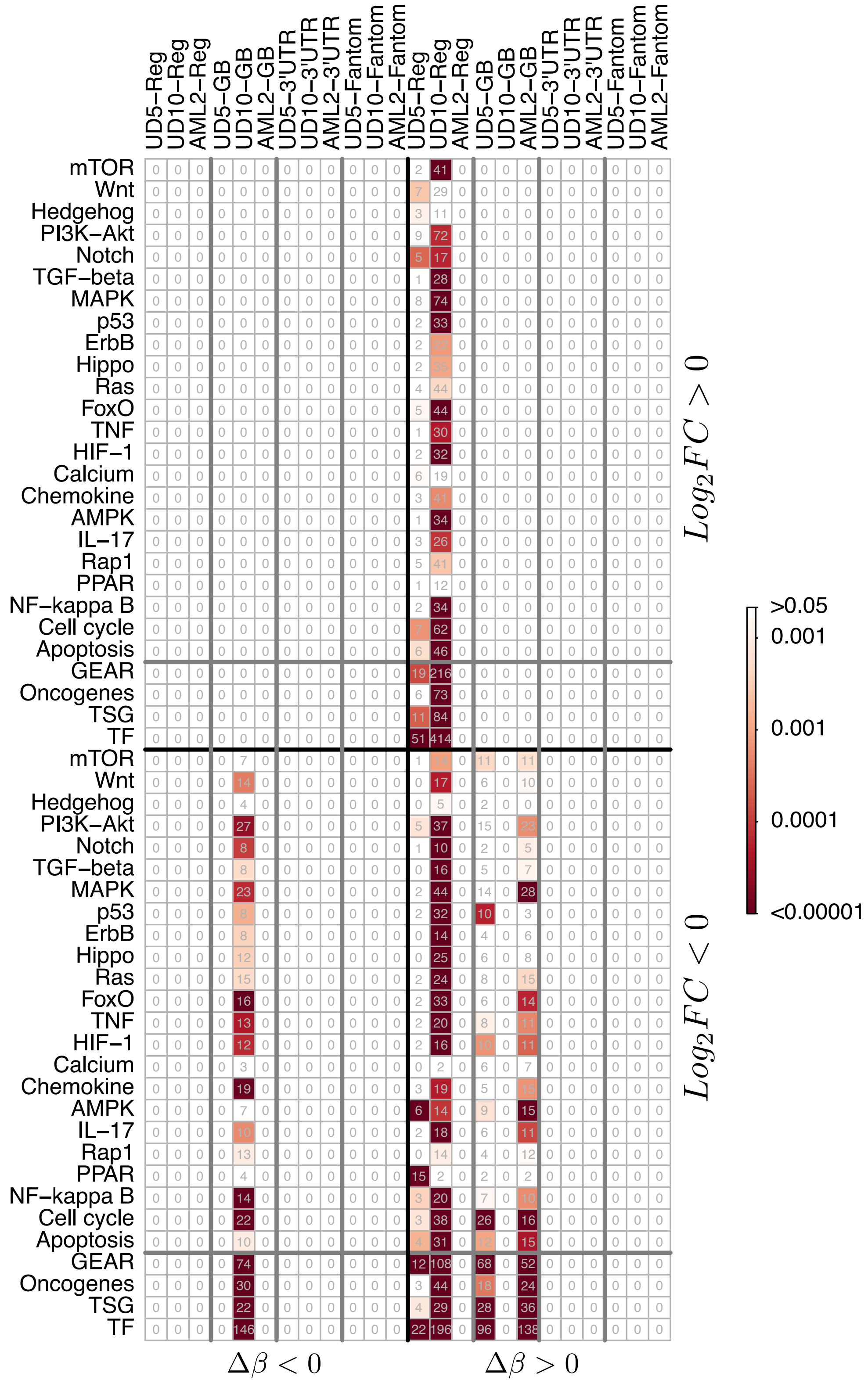

Figure 52: Figure reports the results of ORA on the second-level regulatory network cascade induced by DMRs at CGIs. Each cell of the matrix reports the results of overrepresentation analysis for all the four epigenetic regulation (hypo-methylation/over-expression, hyper-methylation/under-expression, hyper-methylation/over-expression and hypo-methylation/under-expression). The analysis was performed separately for genes affected by DMRs at the 5' regulatory region (Reg), the gene body (GB), the 3'UTR and at the enhancer region predicted by FANTOM. The numbers in each cell represent the total number of genes for each category, while the color intensity reflects statistical significance according to colorbar. Fisher exact test and number of genes were calculated for cancer-related pathways selected from KEGG database, TFs, TSG and Oncogenes selected by COSMIC and GEAR genes.

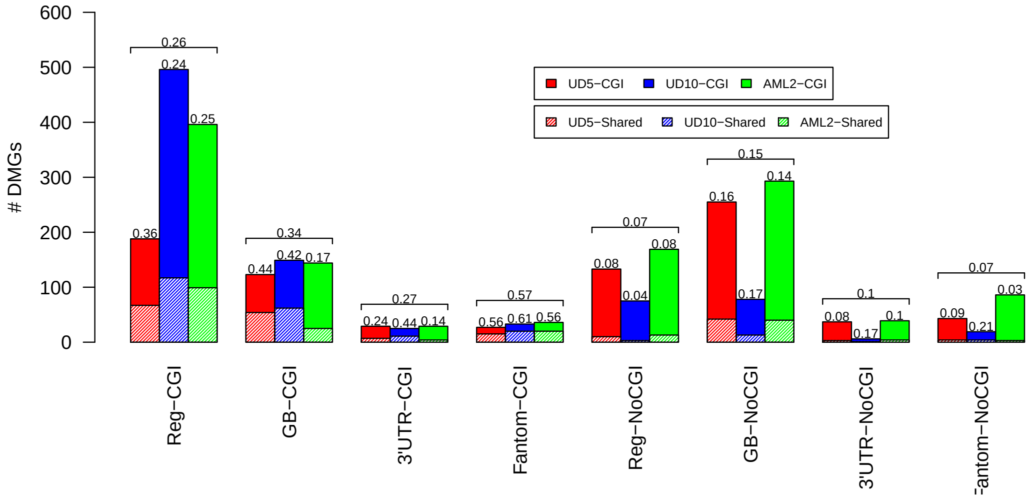

Figure 53: The barplot reports the number of hyper-methylated DMGs shared by each AML sample with the other two samples. Solid bars report the total number of hyper-methylated DMGs, while textured bars the number of shared DMGs. Numbers are reported for DMGs with DMRs at 5' regulatory-elements, gene bodies, 3'UTRs and enhancers (Fantom) overlapping CGIs and sparse CpG regions (NoCGI). Numbers above bars show the percentage of DMGs that are shared with the other two sample. Horizontal brackets above each group of three bars summarize average percentages within the three samples.

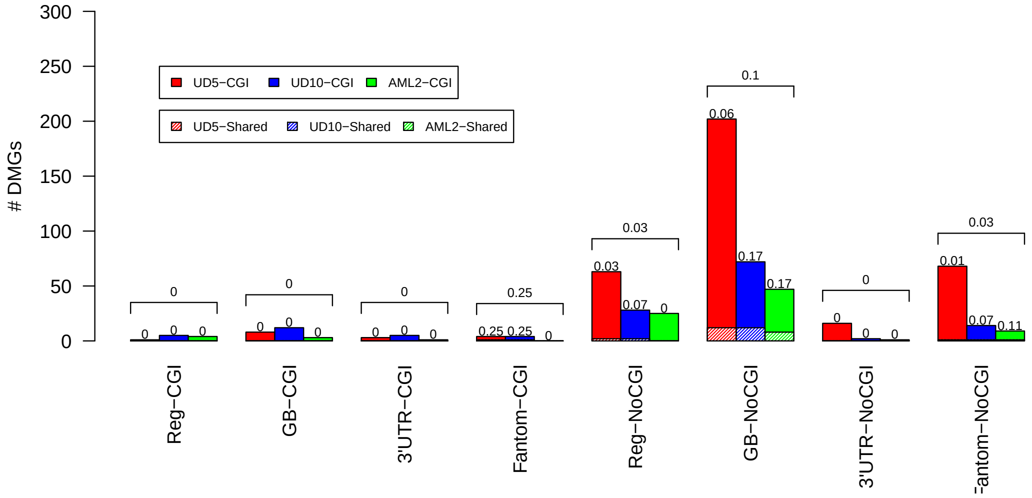

Figure 54: The barplot reports the number of hypo-methylated DMGs shared by each AML sample with the other two samples. Solid bars report the total number of hypo-methylated DMGs, while textured bars the number of shared DMGs. Numbers are reported for DMGs with DMRs at 5' regulatory-elements, gene bodies, 3'UTRs and enhancers (Fantom) overlapping CGIs and sparse CpG regions (NoCGI). Numbers above bars show the percentage of DMGs that are shared with the other two sample. Horizontal brackets above each group of three bars summarize average percentages within the three samples.

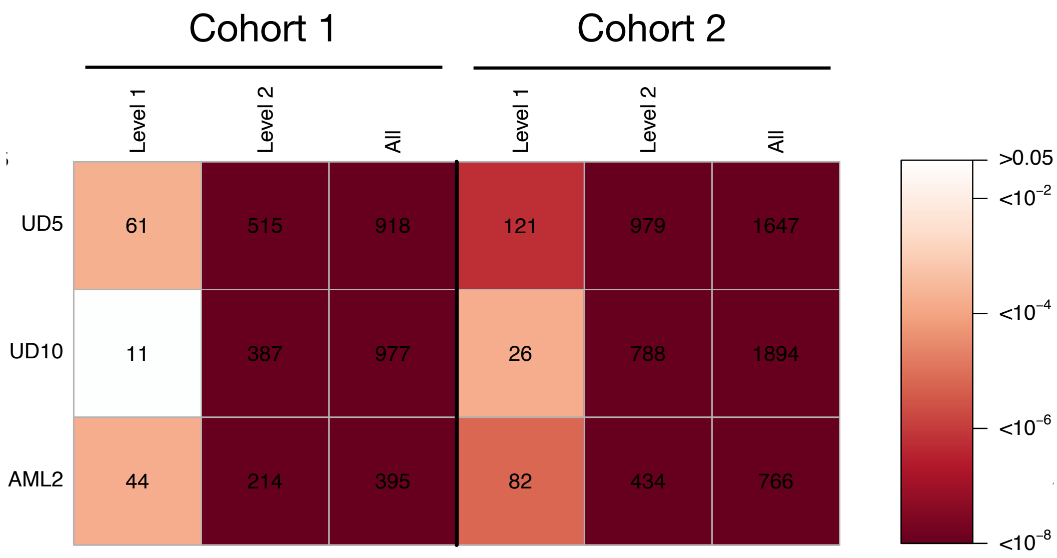

Figure 55: Figure reports the results of overlap analysis between DEGs of our AML samples and the DEGs identified in the two validation cohorts (Cohort 1 is Cocciardi et al., while Cohort 2 is Toffalori et al.). For the three AML samples, overlap analysis was performed for first-level (Level 1), second-level (Level-2) and all DEGs. The numbers in each cell represent the number of overlapped DEGs for each category, while the color intensity reflects statistical significance according to colorbar. Statistical significance was calculated by using Fisher exact test.

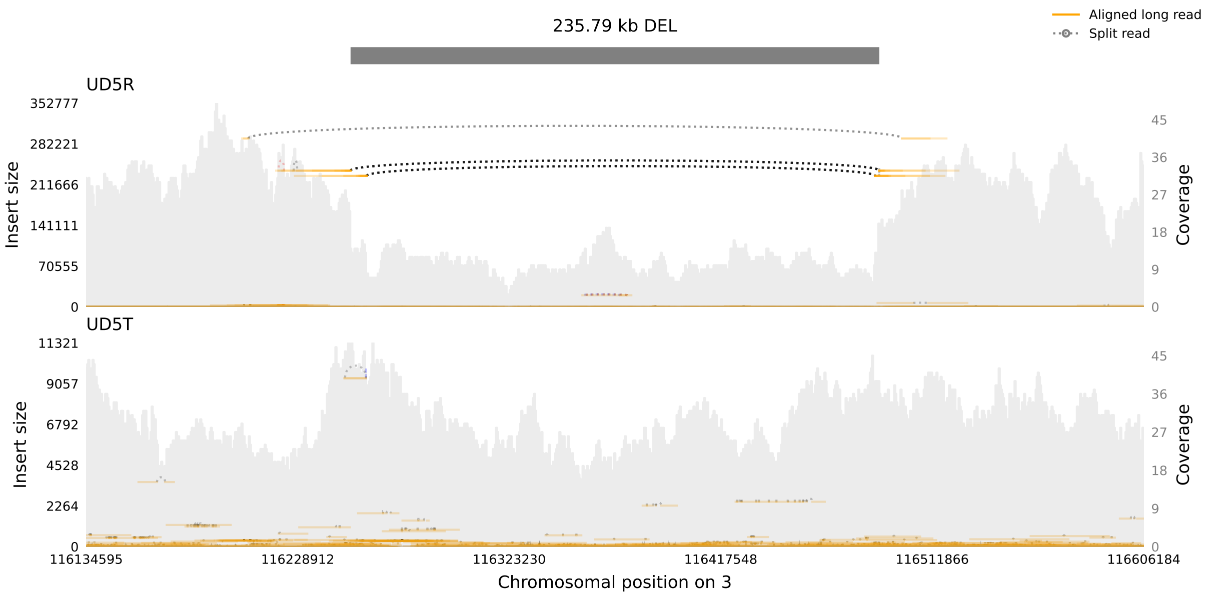

Figure 56: Samplot of the 235 kb deletion detected by NanomonSV on UD5R sample at 3:116259740-116486402. Reads are indicated by horizontal orange lines. Split alignments are indicated by color-coded dotted lines (black for deletions, red for duplications, blue for inversions) that allow to clearly and quickly distinguish between normal reads and reads supporting different SV types. The coverage for the region is shown with the gray-filled background, which is split into map quality above or below a user-defined threshold (in dark or light gray respectively). Split alignment signatures confirms the presence of the deletion in UD5R and the absence in UD5T. The variant is considered true positive.

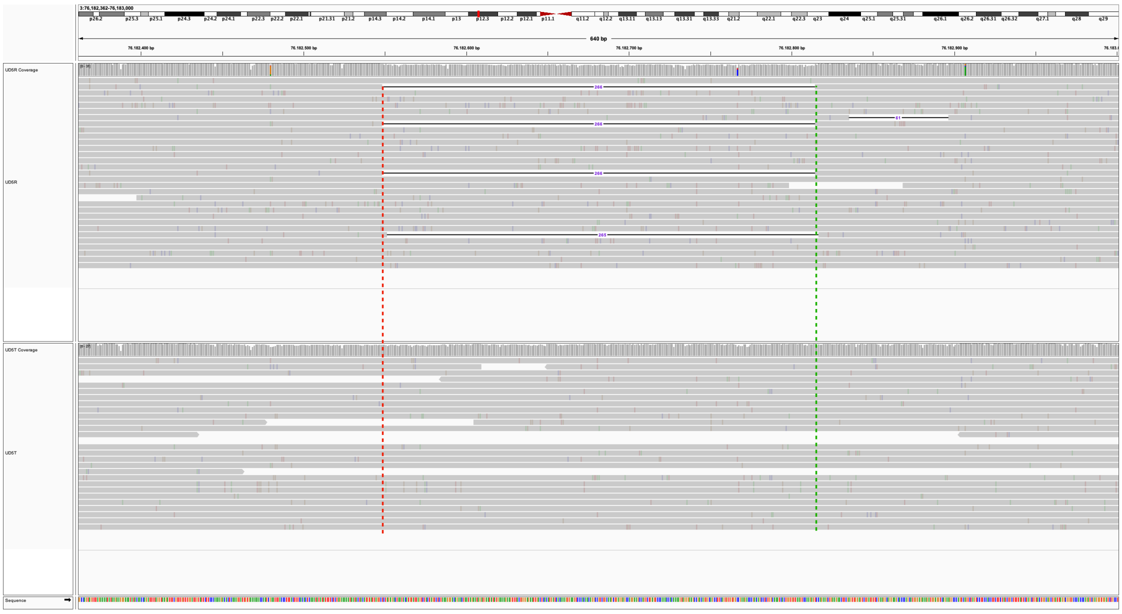

Figure 57: GV plot of the 266 bp relapse-specific deletion detected by NanomonSV on UD5R sample at 3:76182548-76182814. Gray horizontal bars represent aligned reads. Bases that do not match the reference genome are drawn with the letter of the called base (A, C, G, or T). A black line represents a deletion, and a purple box an insertion. Deletions and insertions are labeled with the event size. Vertical dotted lines indicate the start (red) and end (green) positions predicted by NanomonSV. The plot confirms the presence of few deletion signatures in UD5R and no signature in UD5T. The variant is considered true positive.

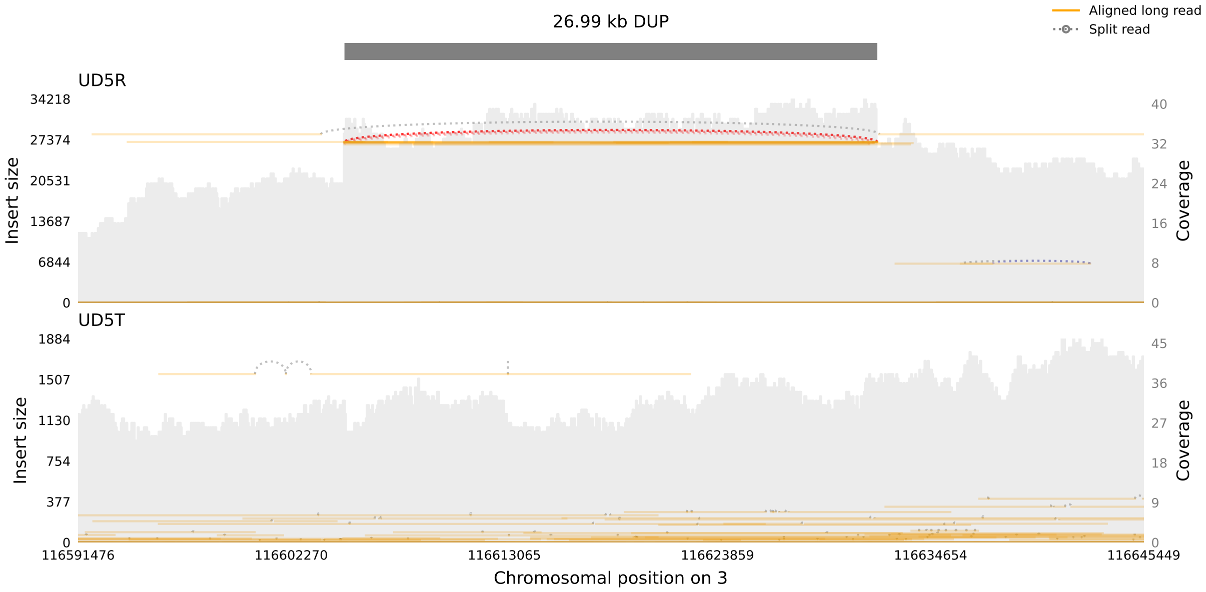

Figure 58: Samplot of the 27 kb duplication detected by NanomonSV on UD5R sample at 3:116604969-116631956. Reads are indicated by horizontal orange lines. Split alignments are indicated by color-coded dotted lines (black for deletions, red for duplications, blue for inversions) that allow to clearly and quickly distinguish between normal reads and reads supporting different SV types. The coverage for the region is shown with the gray-filled background, which is split into map quality above or below a user-defined threshold (in dark or light gray respectively). Split alignment signatures confirms the presence of the duplication in UD5R and the absence in UD5T. The variant is considered true positive.

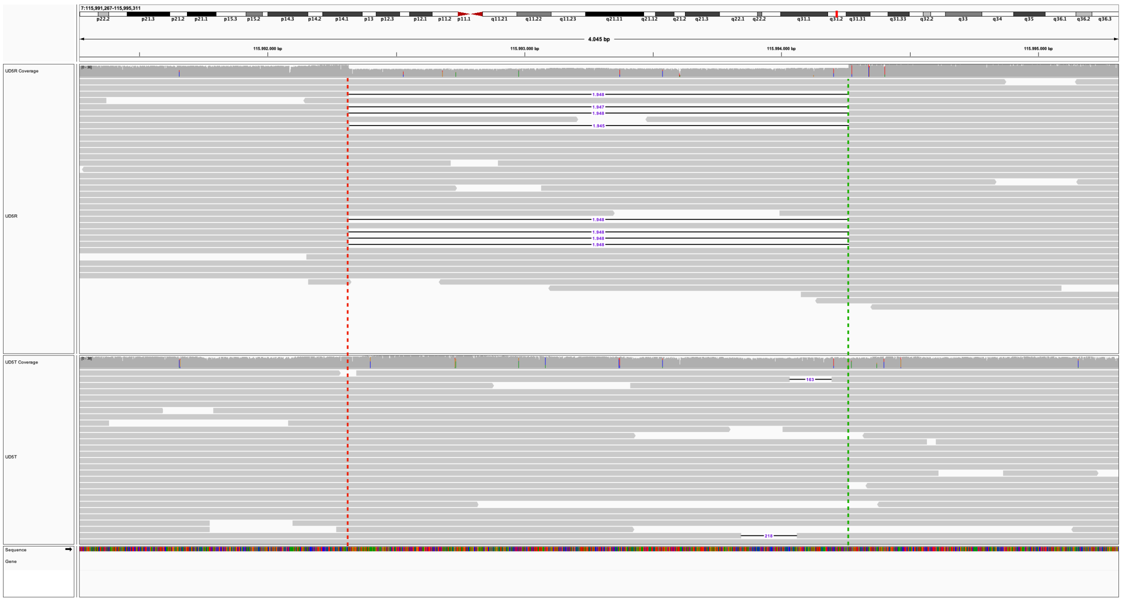

Figure 59: IGV plot of the 1949 bp relapse-specific deletion detected by NanomonSV on UD5R sample at 7:15992315-115994264. Gray horizontal bars represent aligned reads. Bases that do not match the reference genome are drawn with the letter of the called base (A, C, G, or T). A black line represents a deletion, and a purple box an insertion. Deletions and insertions are labeled with the event size. Vertical dotted lines indicate the start (red) and end (green) positions predicted by NanomonSV. The plot confirms the presence of several deletion signatures in UD5R and no signature in UD5T. The variant is considered true positive.

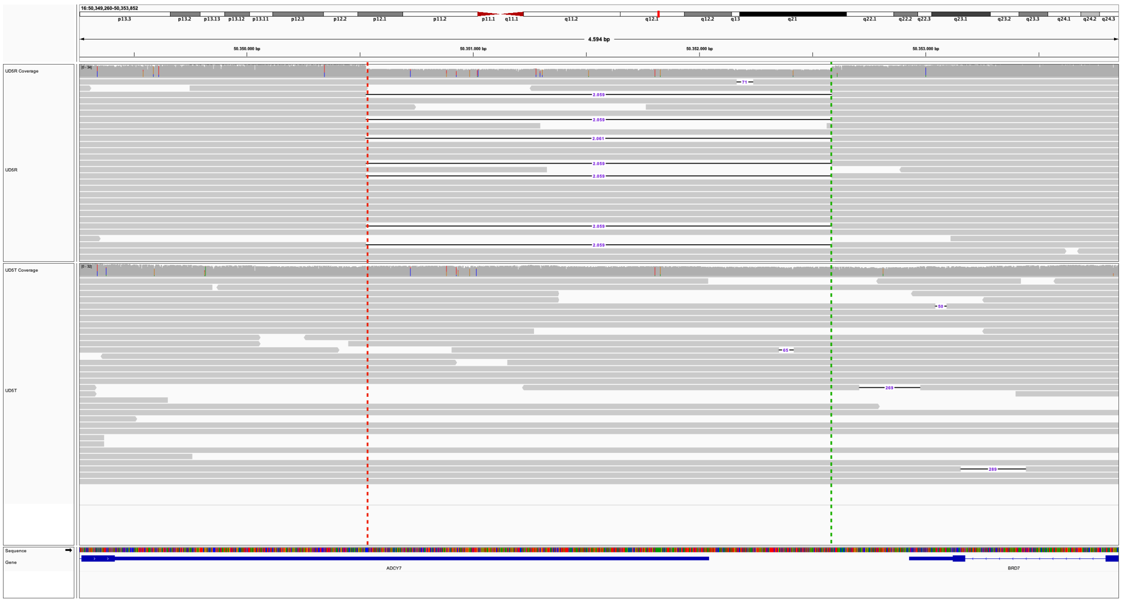

Figure 60: IGV plot of the 2060 bp relapse-specific deletion detected by NanomonSV on UD5R sample at 16:50350527-50352587. Gray horizontal bars represent aligned reads. Bases that do not match the reference genome are drawn with the letter of the called base (A, C, G, or T). A black line represents a deletion, and a purple box an insertion. Deletions and insertions are labeled with the event size. Vertical dotted lines indicate the start (red) and end (green) positions predicted by NanomonSV. The plot confirms the presence of several deletion signatures in UD5R and no signature in UD5T. The variant is considered true positive.

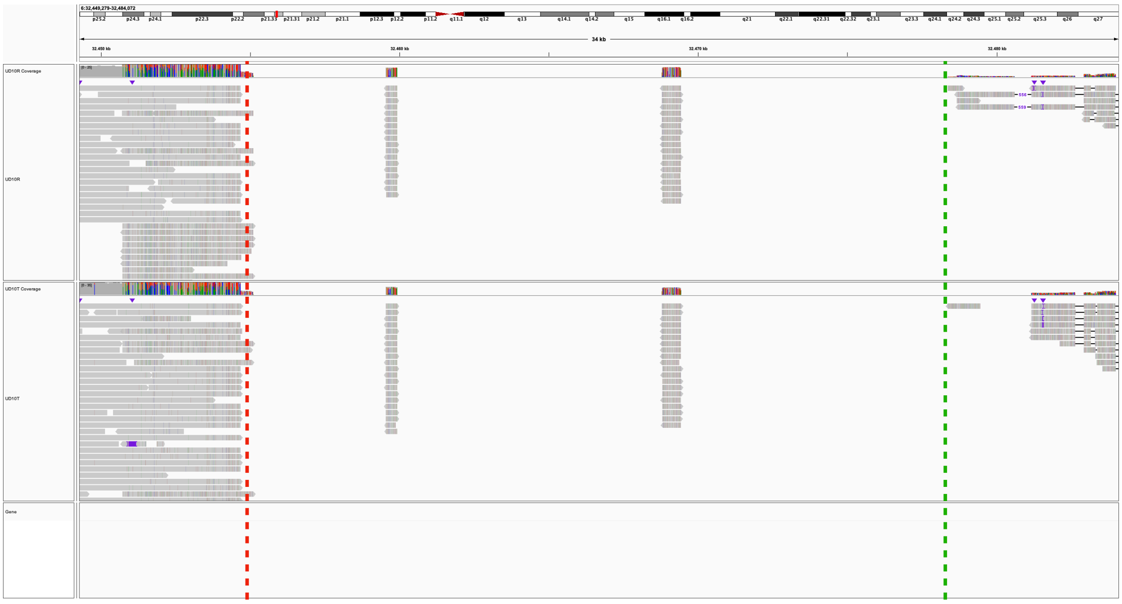

Figure 61: IGV plot of the 24 kb relapse-specific deletion detected by NanomonSV on UD10R sample at 6:32454686-32478665. Gray horizontal bars represent aligned reads. Bases that do not match the reference genome are drawn with the letter of the called base (A, C, G, or T). A black line represents a deletion, and a purple box an insertion. Deletions and insertions are labeled with the event size. Vertical dotted lines indicate the start (red) and end (green) positions predicted by NanomonSV. The plot confirms the presence of several deletion signatures in UD10R and one signature in UD10T. The variant is considered false positive.

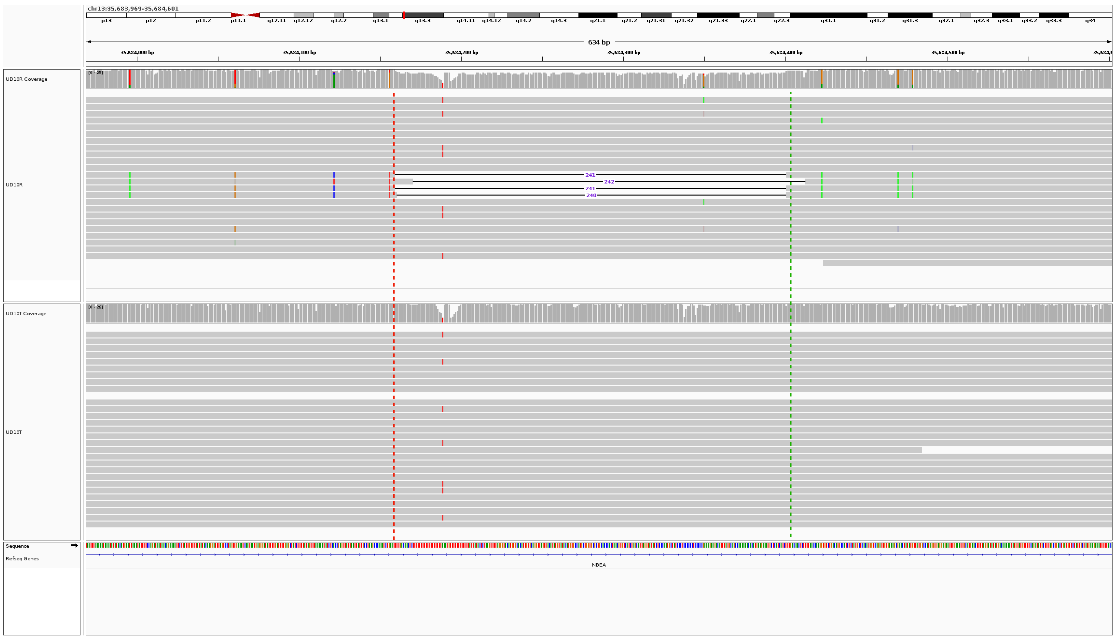

Figure 62: IGV plot of the 242 bp relapse-specific deletion detected by NanomonSV on UD10R sample at 13:35684164-35684406. Gray horizontal bars represent aligned reads. Bases that do not match the reference genome are drawn with the letter of the called base (A, C, G, or T). A black line represents a deletion, and a purple box an insertion. Deletions and insertions are labeled with the event size. Vertical dotted lines indicate the start (red) and end (green) positions predicted by NanomonSV. The plot confirms the presence of several deletion signatures in UD10R and one signature in UD10T. The variant is considered true positive.

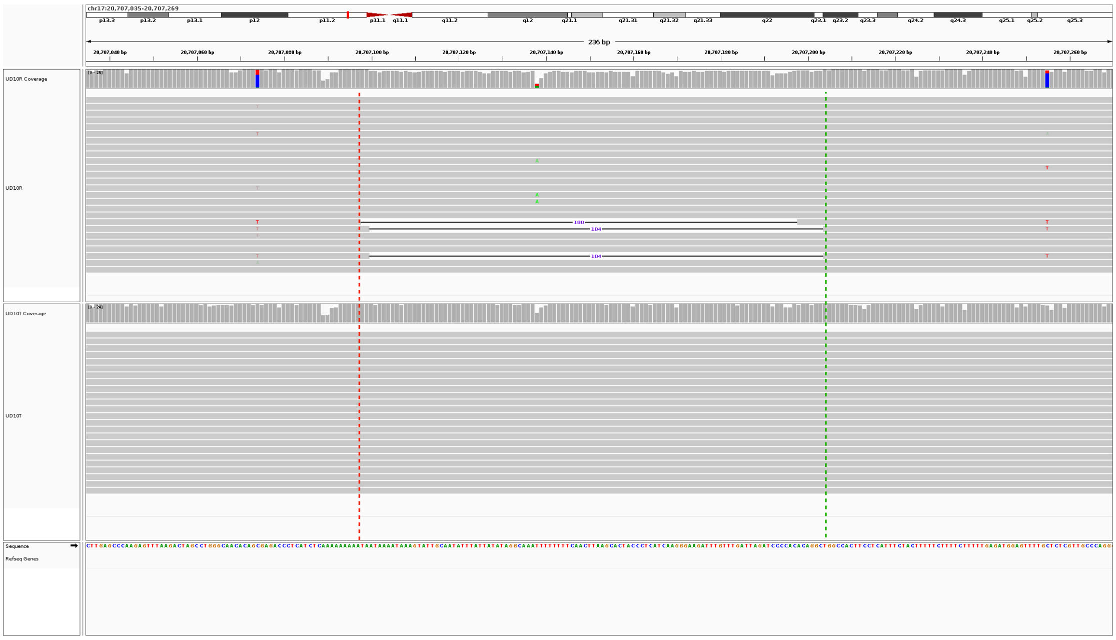

Figure 63: IGV plot of the 106 bp relapse-specific deletion detected by NanomonSV on UD10R sample at 17:20707099-20707205. Gray horizontal bars represent aligned reads. Bases that do not match the reference genome are drawn with the letter of the called base (A, C, G, or T). A black line represents a deletion, and a purple box an insertion. Deletions and insertions are labeled with the event size. Vertical dotted lines indicate the start (red) and end (green) positions predicted by NanomonSV. The plot confirms the presence of several deletion signatures in UD10R and one signature in UD10T. The variant is considered true positive.

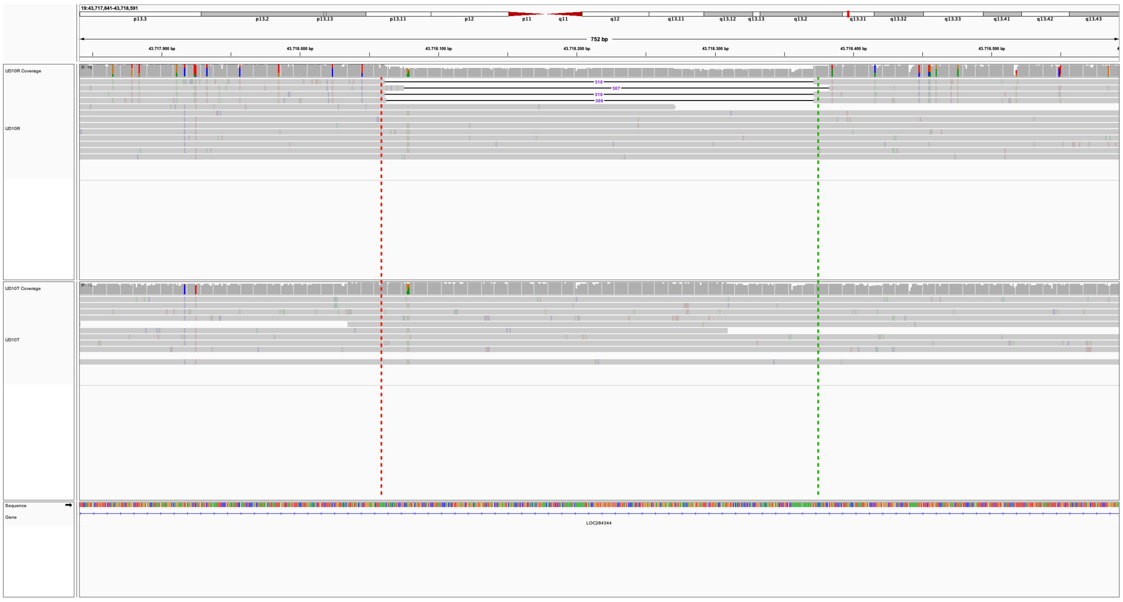

Figure 64: IGV plot of the 310 bp relapse-specific deletion detected by NanomonSV on UD10R sample at 19:43718061-43718372. Gray horizontal bars represent aligned reads. Bases that do not match the reference genome are drawn with the letter of the called base (A, C, G, or T). A black line represents a deletion, and a purple box an insertion. Deletions and insertions are labeled with the event size. Vertical dotted lines indicate the start (red) and end (green) positions predicted by NanomonSV. The plot confirms the presence of several deletion signatures in UD10R and one signature in UD10T. The variant is considered true positive.

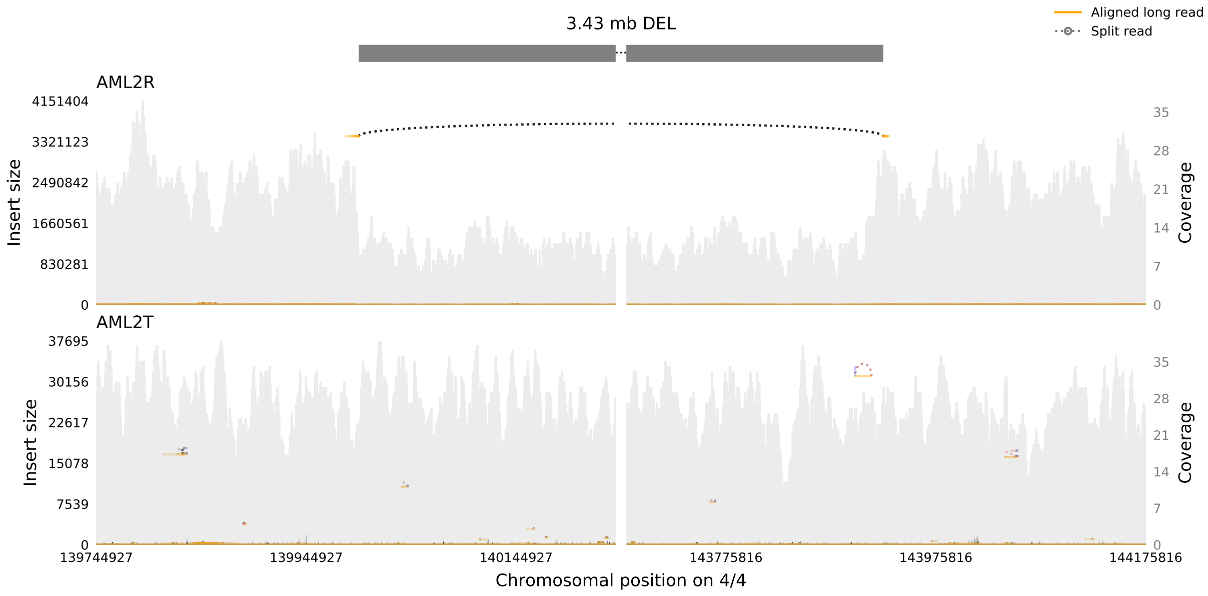

Figure 65: Samplot of the 3.43 Mb deletion detected by NanomonSV on AML2R sample at 4:140244927-143675816. Reads are indicated by horizontal orange lines. Split alignments are indicated by color-coded dotted lines (black for deletions, red for duplications, blue for inversions) that allow to clearly and quickly distinguish between normal reads and reads supporting different SV types. The coverage for the region is shown with the gray-filled background, which is split into map quality above or below a user-defined threshold (in dark or light gray respectively). Split alignment signatures confirms the presence of the deletion in AML2R and the absence in AML2T. The variant is considered true positive.

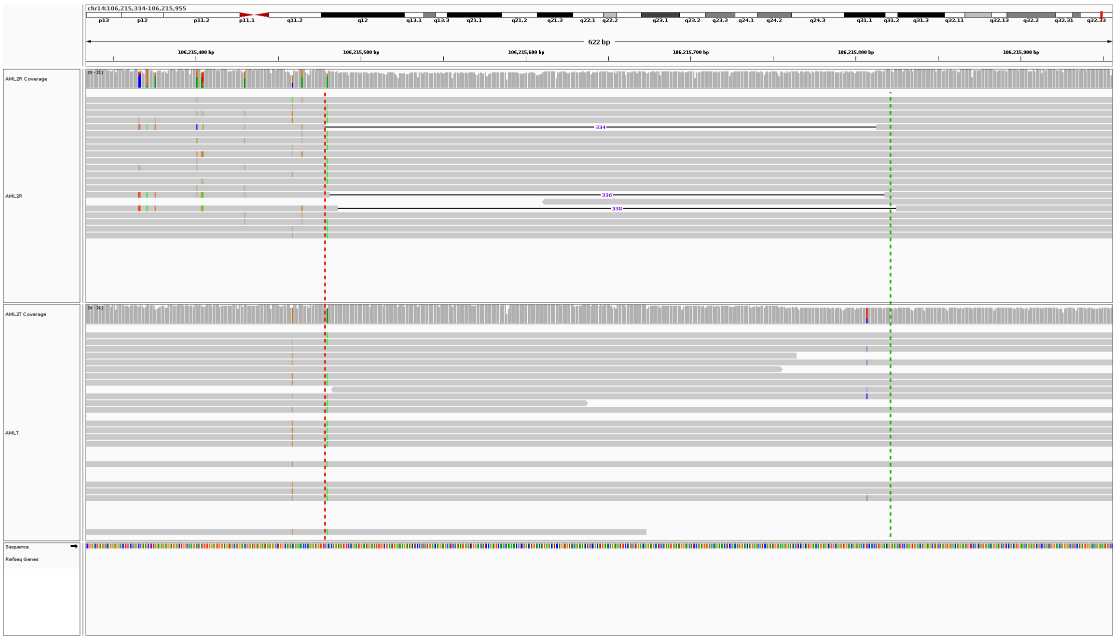

Figure 66: IGV plot of the 273 bp relapse-specific deletion detected by NanomonSV on UD10R sample at 14:106215508-106215781. Gray horizontal bars represent aligned reads. Bases that do not match the reference genome are drawn with the letter of the called base (A, C, G, or T). A black line represents a deletion, and a purple box an insertion. Deletions and insertions are labeled with the event size. Vertical dotted lines indicate the start (red) and end (green) positions predicted by NanomonSV. The plot confirms the presence of several deletion signatures in AML2R and one signature in AML2T. The variant is considered true positive.

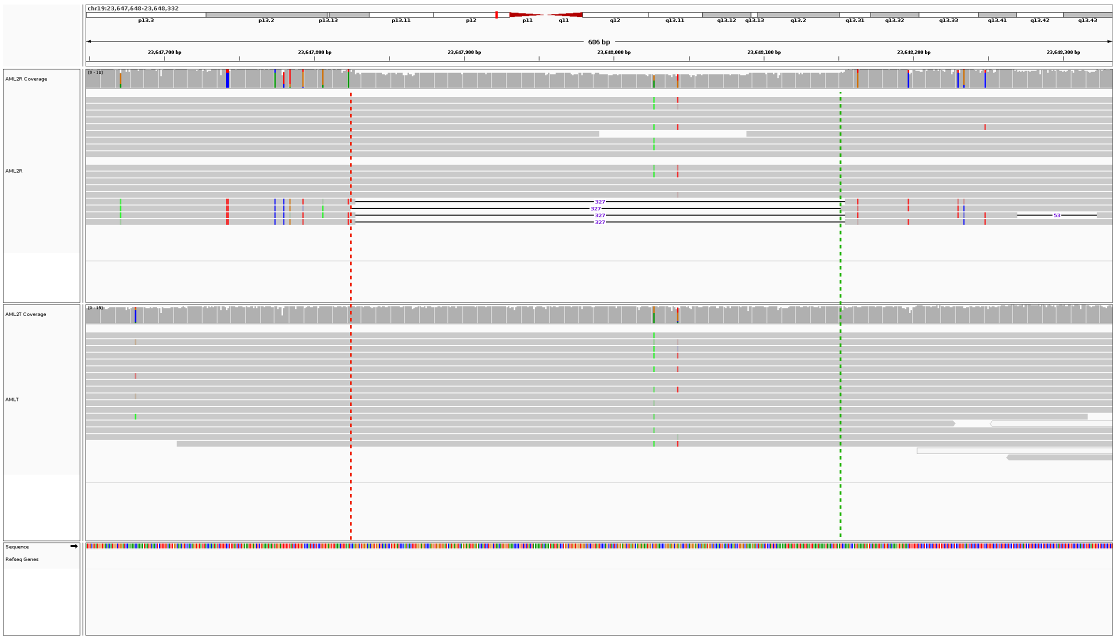

Figure 67: IGV plot of the 327 bp relapse-specific deletion detected by NanomonSV on UD10R sample at 19:23647827-23648154. Gray horizontal bars represent aligned reads. Bases that do not match the reference genome are drawn with the letter of the called base (A, C, G, or T). A black line represents a deletion, and a purple box an insertion. Deletions and insertions are labeled with the event size. Vertical dotted lines indicate the start (red) and end (green) positions predicted by NanomonSV. The plot confirms the presence of several deletion signatures in AML2R and one signature in AML2T. The variant is considered true positive.

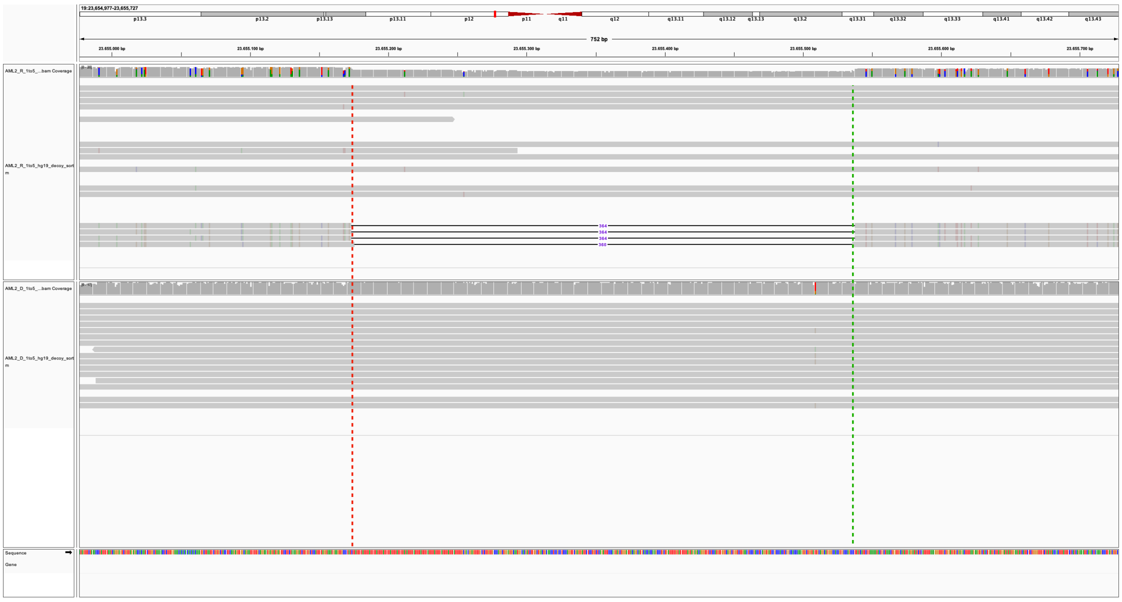

Figure 68: IGV plot of the 370 bp relapse-specific deletion detected by NanomonSV on UD10R sample at 19:23655167-23655538. Gray horizontal bars represent aligned reads. Bases that do not match the reference genome are drawn with the letter of the called base (A, C, G, or T). A black line represents a deletion, and a purple box an insertion. Deletions and insertions are labeled with the event size. Vertical dotted lines indicate the start (red) and end (green) positions predicted by NanomonSV. The plot confirms the presence of several deletion signatures in AML2R and one signature in AML2T. The variant is considered true positive.
