# Supplementary material for: High-resolution Nanopore methylome-maps reveal random hyper-methylation at CpG-poor regions as driver of chemoresistance in leukemias
Source: Commun Biol. 2023 Apr 8;6:382. doi: 10.1038/s42003-023-04756-8 (PMC10082806; doi:10.1038/s42003-023-04756-8)
Supplement: Supplementary file 3 — Description of Additional Supplementary Files [file 42003_2023_4756_MOESM3_ESM.pdf]

Description of Additional Supplementary Files

File name: Supplemental Data 1
Description: Excel file of all DMRs.

File name: Supplemental Data 2
Description: Excel file of all DEGs.

File name: Supplemental Data 3
Description: Excel file of GRNs induced by DM-DE-TF with DMRs in gene-body.
